# Glucocorticoids Acutely Increase Brown Adipose Tissue Activity in Humans, Revealing Species-Specific Differences in UCP-1 Regulation

**DOI:** 10.1016/j.cmet.2016.06.011

**Published:** 2016-07-12

**Authors:** Lynne E. Ramage, Murat Akyol, Alison M. Fletcher, John Forsythe, Mark Nixon, Roderick N. Carter, Edwin J.R. van Beek, Nicholas M. Morton, Brian R. Walker, Roland H. Stimson

**Affiliations:** 1 British Heart Foundation/University Centre for Cardiovascular Science, University of Edinburgh, Edinburgh EH16 4TJ, Scotland, UK; 2 Department of Surgery, Royal Infirmary of Edinburgh, Edinburgh EH16 4SA, Scotland, UK; 3 Clinical Research Imaging Centre, University of Edinburgh, Edinburgh EH16 4TJ, Scotland, UK

## Abstract

The discovery of brown adipose tissue (BAT) in adult humans presents a new therapeutic target for metabolic disease; however, little is known about the regulation of human BAT. Chronic glucocorticoid excess causes obesity in humans, and glucocorticoids suppress BAT activation in rodents. We tested whether glucocorticoids regulate BAT activity in humans. In vivo, the glucocorticoid prednisolone acutely increased ^18^fluorodeoxyglucose uptake by BAT (measured using PET/CT) in lean healthy men during mild cold exposure (16°C–17°C). In addition, prednisolone increased supraclavicular skin temperature (measured using infrared thermography) and energy expenditure during cold, but not warm, exposure in lean subjects. In vitro, glucocorticoids increased isoprenaline-stimulated respiration and UCP-1 in human primary brown adipocytes, but substantially decreased isoprenaline-stimulated respiration and UCP-1 in primary murine brown and beige adipocytes. The highly species-specific regulation of BAT function by glucocorticoids may have important implications for the translation of novel treatments to activate BAT to improve metabolic health.

## Highlights

• Glucocorticoids acutely increase but chronically suppress BAT activity in humans
• Glucocorticoids increase UCP-1 and respiration in human brown adipocytes
• Glucocorticoids decrease UCP-1 and respiration in murine brown and beige adipocytes
• Species-specific differences exist in the regulation of BAT activation

## Graphical Abstract

**Figure:**
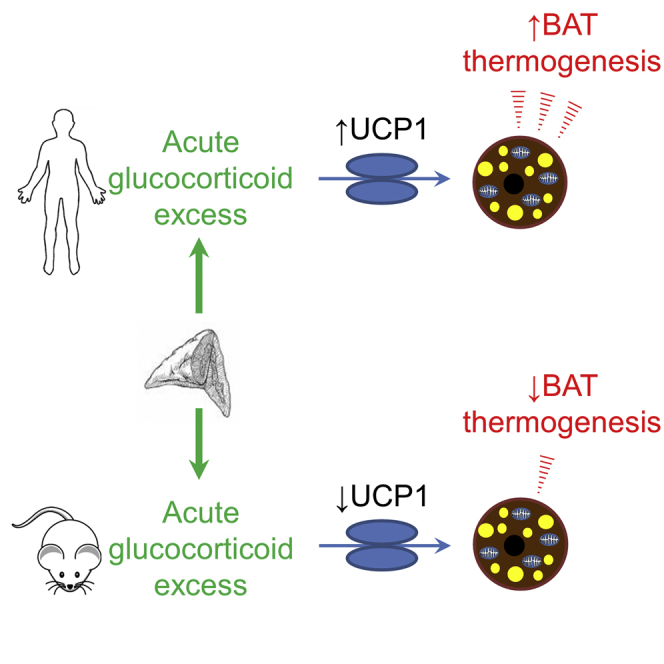

## Teaser

The regulation of brown adipose tissue (BAT) in humans is not well understood. Ramage et al. show that glucocorticoids acutely increase BAT in vivo and in vitro in humans through increasing UCP-1. However, glucocorticoids decrease UCP-1 in murine beige/ brown adipocytes, identifying species-specific differences in the regulation of BAT function.

## Introduction

The recent discovery of brown adipose tissue (BAT) in adult humans (Virtanen et al., 2009, van Marken Lichtenbelt et al., 2009, Saito et al., 2009) has revealed the exciting possibility of activating BAT and/or “browning” white adipose tissue (WAT) to enhance energy expenditure and treat obesity, diabetes, and even dyslipidemia (Sidossis and Kajimura, 2015). Numerous factors have been identified that induce browning in rodents, such as FGF21, BMP8b, and natriuretic peptides (Sidossis and Kajimura, 2015). However, very little is known about the function and regulation of human BAT. Findings to date suggest similarities between rodent and human BAT, for example, both are activated by cold exposure (Saito et al., 2009) and sympathetic activity (Cypess et al., 2015) and contribute to cold-induced thermogenesis (Ouellet et al., 2012). Recent work has focused on whether human BAT is more closely related to rodent”classic” brown or inducible “beige” fat (Jespersen et al., 2013, Cypess et al., 2013, Shinoda et al., 2015), with less emphasis on determining if there are substantial differences between species in the regulation of BAT function. Analysis of hormonal regulation of BAT in humans has been largely overlooked to date, in part because of the difficulty in obtaining samples for in vitro studies or quantifying in vivo function of human BAT.

Glucocorticoids are powerful regulators of energy metabolism, and chronic glucocorticoid excess causes obesity, type 2 diabetes mellitus, and dyslipidemia (Macfarlane et al., 2008). In rodents, it has long been known that glucocorticoids suppress UCP-1 and thermogenesis by BAT, while adrenalectomy and the glucocorticoid receptor antagonist RU38486 both increase BAT thermogenesis and UCP-1 levels (Hardwick et al., 1989, Strack et al., 1995, van den Beukel et al., 2014). In addition, glucocorticoids suppress browning of WAT in mice (Kong et al., 2015). While circulating glucocorticoid levels are regulated by the hypothalamic-pituitary-adrenal (HPA) axis, tissue glucocorticoid levels in important metabolic tissues such as liver and adipose tissue are further amplified by the enzyme 11β-hydroxysteroid dehydrogenase type 1 (11β-HSD1) (Stimson et al., 2009). In murine brown adipocytes, overexpression of 11β-HSD1 decreases BAT function, while pharmacological 11β-HSD1 inhibition or knockdown enhances BAT activation (Liu et al., 2013), highlighting that local glucocorticoid excess inhibits BAT activity in rodents. We tested the hypothesis that glucocorticoids suppress BAT activation in humans as in rodents using in vivo and in vitro approaches.

## Results

### Glucocorticoids Increase Cold-Induced ^18^FDG Uptake by Human BAT In Vivo

**Figure 1:**
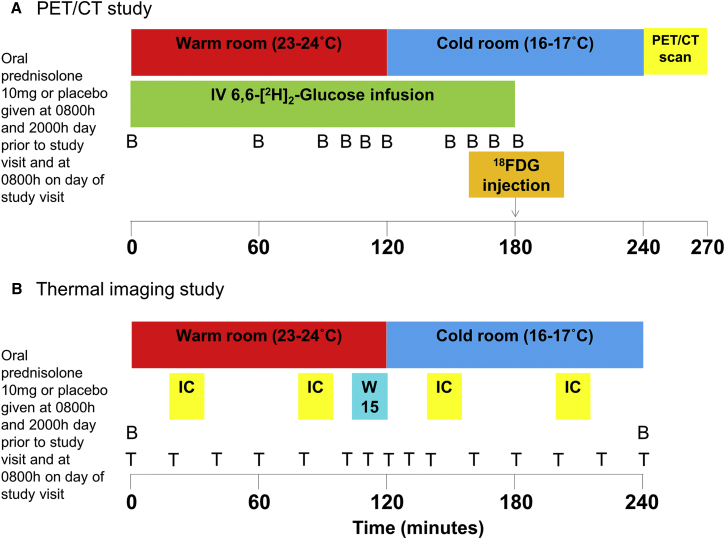
In Vivo Study Visit Protocols Prednisolone regulation of human BAT activity was measured in vivo in two separate studies using (A) PET/CT and (B) thermal imaging. In both studies, participants spent 2 hr in a warm room, then 2 hr in a cold room to activate BAT. In the PET/CT study, 1 hr into cold exposure, subjects were injected with 75 MBq of 18-fluoro-2-deoxyglucose (^18^FDG), and a PET/CT scan was performed 1 hr later. Blood (B) samples were taken at the intervals shown. In the thermal imaging study, at the end of the second hour in the warm room, subjects placed both hands in water cooled to 15°C (W15) as a mild stimulant to activate BAT. Thermal imaging was performed at the intervals shown (T). Whole-body energy expenditure was assessed each hour using indirect calorimetry (IC).

**Figure 2:**
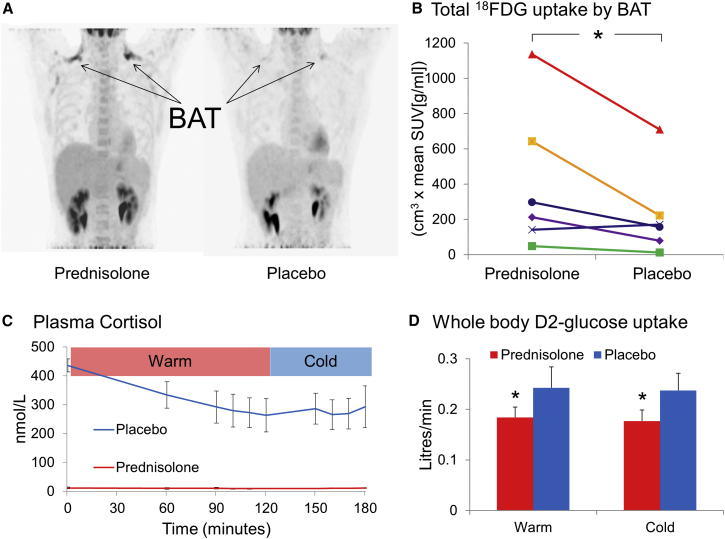
Glucocorticoids Increase Cold-Induced ^18^FDG Uptake by BAT (A) Paired PET/CT scans from a subject demonstrating increased ^18^FDG uptake in the supraclavicular BAT (arrows) during the prednisolone phase (left panel). (B) Paired total ^18^FDG uptake by BAT for all six subjects; prednisolone increased uptake by BAT compared with placebo. Data were analyzed using the Wilcoxon signed-rank test. (C) Plasma cortisol concentrations, shown as mean ± SEM (n = 6), fell following normal diurnal variation in the warm room (p < 0.05 using repeated-measures ANOVA) but did not continue to fall during cooling (t + 120 onward) during the placebo phase (blue line), and were appropriately suppressed by prednisolone (red line). (D) Prednisolone (red columns) decreased whole-body D2-glucose uptake compared with placebo (blue columns); whole-body D2-glucose uptake was not altered by cold exposure. Data were analyzed using the paired t test. ^∗^p < 0.05, prednisolone versus placebo.

Six healthy men (subject characteristics in Table 1) were recruited to a randomized double-blind, placebo-controlled crossover study (Figure 1A) to determine the effect of glucocorticoids on BAT activity. The synthetic glucocorticoid prednisolone (10 mg every 12 hr) or placebo was administered for three doses prior to each study visit, and positron emission tomography/computed tomography (PET/CT) was used to measure ^18^fluorodeoxyglucose (^18^FDG) uptake by BAT during 2 hr of mild cold exposure (16°C–17°C) (van Marken Lichtenbelt et al., 2009). Study visits took place between March and September in Edinburgh. Average local environmental temperatures over that period are detailed in Table S1, available online. No subject had any symptoms or signs of shivering during cold exposure. Prednisolone increased fasting plasma glucose, insulin, and non-esterified fatty acids (NEFAs) and decreased adrenocorticotrophic hormone (ACTH) and cortisol concentrations compared with placebo, but did not alter noradrenaline concentrations (Table 1). All six subjects had detectable ^18^FDG uptake by BAT. Prednisolone increased cold-induced ^18^FDG uptake by BAT (median total standardized uptake value 254 [interquartile range, IQR 159, 556] versus 164 [98, 209] cm^3^ × g/mL, p = 0.02) (Figures 2A and 2B). In addition, prednisolone tended to increase the volume of active BAT (71 ± 25 versus 50 ± 25 cm^3^, p = 0.07).

During the placebo phase, plasma cortisol concentrations fell following normal diurnal variation while subjects were in a warm environment, but did not fall further during cold exposure (Figure 2C). Despite increasing glucose uptake by BAT, prednisolone substantially decreased whole-body 6,6-[^2^H]_2_-glucose (D2-glucose) uptake during both warm and cold conditions, while whole-body D2-glucose uptake was unchanged by cooling on either placebo or prednisolone phases (Figure 2D).

### Glucocorticoids Increase Energy Expenditure and Supraclavicular Temperature during Cold Exposure

**Figure 3:**
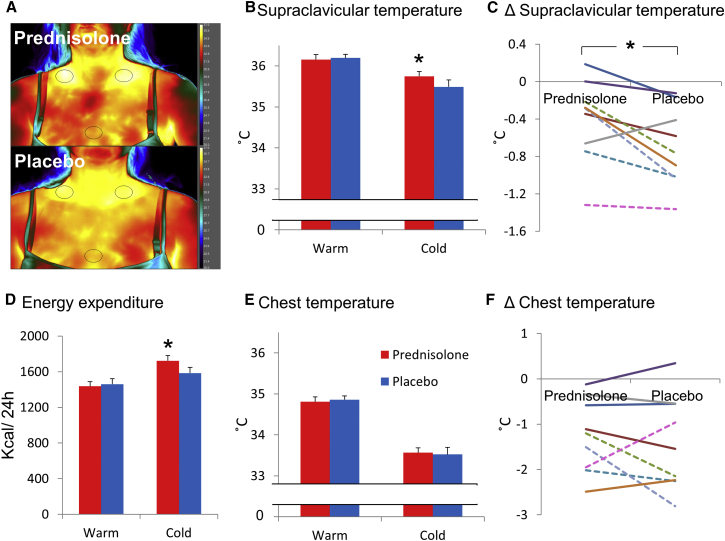
Glucocorticoids Increase Supraclavicular Skin Temperature and Cold-Induced Thermogenesis (A) Paired thermal images obtained from a subject on prednisolone (top panel) and placebo (lower panel) phases during cold exposure, showing increased skin temperature in the supraclavicular regions of interest (black ovals) on the prednisolone phase. (B) Prednisolone (red columns) increased supraclavicular skin temperature compared with placebo (blue columns) during cold exposure only; shown as mean ± SEM (n = 9). Data are analyzed by paired t tests. (C) The reduction in mean supraclavicular skin temperature in male (solid lines) and female (dotted lines) subjects, expressed as the absolute difference between warm and cold environment, induced by cold exposure, was attenuated by prednisolone treatment. (D) Prednisolone increased energy expenditure during cold, but not warm, exposure. (E and F) Prednisolone did not alter (E) chest temperature during warm or cold exposure and did not affect (F) the reduction in mean chest temperature induced by cold compared to warm exposure. ^∗^p < 0.05 versus placebo.

The PET study showed, surprisingly, that glucocorticoids increased cold-induced ^18^FDG uptake by BAT, suggesting that glucocorticoids may increase BAT thermogenesis in humans, unlike in rodents. To test the effect of prednisolone on cold-induced thermogenesis (using indirect calorimetry) and heat production by BAT (using thermal imaging; Lee et al., 2011), we performed a similar placebo-controlled, randomized crossover study (Figure 1B) using the same dosing structure. Subject characteristics are detailed in Table 1. Study visits took place between January and May in the same research facility. Average local environmental temperatures over that period are detailed in Table S1. No subject had any symptoms or signs of shivering during cold exposure. As in the PET study, prednisolone increased fasting plasma glucose and insulin and suppressed ACTH (Table 1) and cortisol (10 ± 3 versus 417 ± 22 nmol/L) concentrations. Prednisolone did not alter resting energy expenditure in a warm environment, but increased energy expenditure during cold exposure (Figure 3D). Prednisolone substantially increased cold-induced thermogenesis compared with placebo (286 ± 68 versus 125 ± 37 kcal/24 hr, p = 0.01).

Prednisolone did not alter skin temperature in the supraclavicular region (BAT) or anterior chest (control) regions in warm conditions (Figures 3B and 3E). In addition, supraclavicular skin temperature did not significantly change following hand immersion in 15°C water for 10 min during either prednisolone or placebo phases (+0.0°C ± 0.1°C and +0.1°C ± 0.1°C, respectively). However, during cold exposure prednisolone increased supraclavicular skin temperature compared with placebo (Figures 3A–3C) but did not alter anterior chest (Figures 3E and 3F) or peripheral (hand) skin temperature (26.1°C ± 0.7°C versus 25.5°C ± 0.7°C).

### Distinct Regulation of Human BAT Activity by Glucocorticoids In Vitro

#### BAT Biopsy and Culture from Human Deep Supraclavicular Adipose Depots

A significant challenge to investigating BAT regulation in humans has been the shortage of reliable sources of tissue from which to culture brown adipocytes. To investigate the mechanism whereby glucocorticoids enhance human BAT activation, we collected adipose tissue samples from euthyroid patients undergoing elective thyroid or parathyroid surgery. Characteristics of the participants are detailed in Table S2.

**Figure 4:**
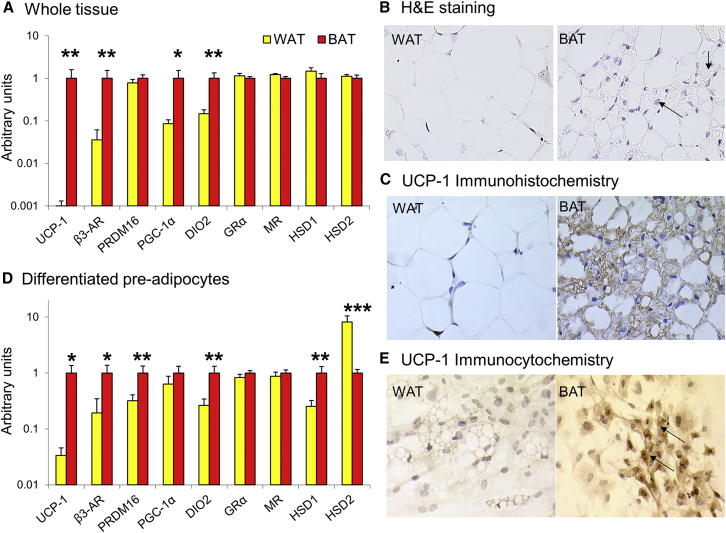
Human BAT Biopsy and Cell Culture (A) mRNA transcripts are shown as mean ± SEM for n = 9 for whole adipose tissue collected from the superficial (WAT, yellow columns) and deeper (BAT, red columns) neck depots. (B) Images obtained using 40× magnification show that the superficial depot only contained adipocytes with the typical appearance of white adipocytes, while the deeper depot also contained smaller cells with multilocular lipid droplets, more rounded nuclei, and basophilic cytoplasm (arrows), which are the typical features of brown adipocytes. (C) Immunohistochemistry (40× magnification) showing that only the brown adipocyte-like cells contained UCP-1 in their cytoplasm (brown staining). (D) mRNA transcripts are shown as mean ± SEM for n = 11 for differentiated pre-adipocytes cultured from the superficial (WAT) and the deeper (BAT) depots, showing that the BAT cells maintained a similar gene expression pattern to the whole tissue. (E) Immunohistochemistry (40× magnification) showing that only the cultured cells from the BAT depot contained UCP-1 (brown staining, black arrows) in their cytoplasm. Data were analyzed using paired t tests. ^∗^p < 0.05, ^∗∗^p < 0.01, ^∗∗∗^p < 0.001 versus WAT.

Initial work focused on obtaining human BAT without prior PET/CT scanning. Paired samples were obtained from the superficial WAT and adipose tissue depots posterior to the thyroid (Figure S1), the typical location of human BAT (Cypess et al., 2013). The deeper tissue had substantially higher transcript levels of the typical BAT genes UCP-1 (∼1,000-fold), β3-adrenoreceptor (β3-AR), PGC-1α, and deiodinase type 2 (DIO2) (Figure 4A). Furthermore, although both depots consisted predominantly of white adipocytes, only tissue from the deeper depot also contained clusters of cells with the typical appearance of brown adipocytes, and immunohistochemistry confirmed only these cells contained UCP-1 (Figures 4B and 4C), proving that BAT could be successfully obtained from humans without the need for prior PET/CT scanning. mRNA levels of the glucocorticoid receptor-α (GRα), mineralocorticoid receptor (MR), cortisol-regenerating enzyme 11β-hydroxysteroid dehydrogenase type 1 (11β-HSD1), and cortisol-inactivating enzyme 11β-HSD2 were similarly expressed in BAT and WAT, indicating that human BAT is likely to be a glucocorticoid-responsive tissue (Figure 4A). To determine whether obesity was associated with dysregulated glucocorticoid signaling in BAT, correlations between body mass index (BMI) and GRα, MR, 11β-HSD1, and 11β-HSD2 mRNA levels were tested in an expanded dataset (n = 21). 11β-HSD2 mRNA levels positively correlated with BMI in BAT (r^2^ = 0.31, p < 0.05), but not WAT (r^2^ = 0.00), while no other significant associations were found.

In a separate patient cohort, we cultured the stromal vascular fraction from the superficial and deeper supraclavicular adipose tissue. Differentiated pre-adipocytes from the deeper depot (hereafter termed brown adipocytes) retained high expression of the typical BAT genes UCP-1, β3-AR, PRDM16, and DIO2 compared with differentiated pre-adipocytes from the superficial depot (termed white adipocytes) (Figure 4D). Furthermore, immunocytochemistry revealed that only the cultured brown adipocytes contained UCP-1 (Figure 4E). The brown adipocytes had substantially higher expression of 11β-HSD1 and lower expression of 11β-HSD2, while GRα and MR transcript levels were similar between cell types (Figure 4D).

Recent conflicting reports suggest human BAT is more akin either to rodent “beige” or to interscapular “classic” BAT (Shinoda et al., 2015, Cypess et al., 2013, Jespersen et al., 2013). We measured transcripts of recently published classic brown and beige genes, and determined that human brown adipocytes have high expression of the beige genes TMEM26 and Tbx1, but also high expression of the typical classic brown genes ZIC1 and Ebf3 (Figure S2). In addition, mRNA levels of the recently described brown and beige adipocyte marker Ebf2 (Wang et al., 2014) were higher in the brown than white adipocytes (0.89 ± 0.17 versus 0.62 ± 0.13 a.u., p < 0.04). This confirms previous work suggesting the molecular signature partially overlaps (Cypess et al., 2013, Jespersen et al., 2013), although this may vary between different depots of human BAT.

#### Glucocorticoid Effects on Cultured Human White and Brown Adipocytes

**Figure 5:**
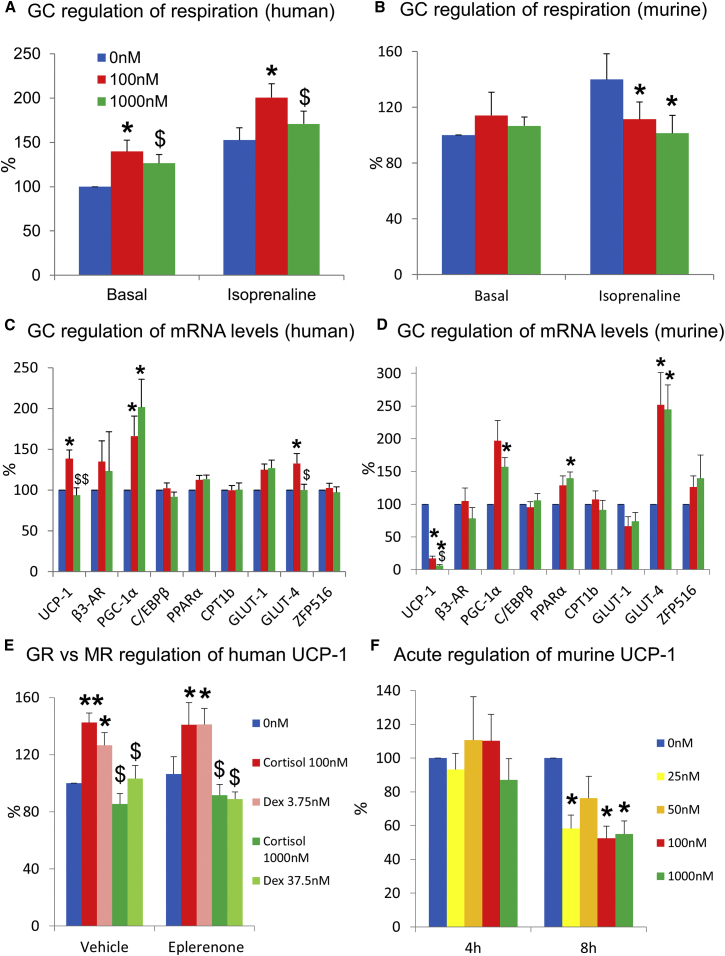
Glucocorticoid Regulation of Human and Murine Brown Adipocytes In Vitro (A and B) Data are mean ± SEM for paired (A) human brown adipocytes (n = 6) or (B) murine inguinal adipocytes (n = 7) cultured for 24 hr in either 0 (blue columns), 100 (red columns), or 1,000 nM (green columns) cortisol. Cortisol (100 nM) increased basal and isoprenaline-stimulated oxygen consumption compared with 0 and 1,000 nM in the human brown adipocytes, but 100 and 1,000 nM cortisol decreased isoprenaline-stimulated oxygen consumption in the murine adipocytes, with basal 0 nM normalized to 100%. (C and D) mRNA levels (with 0 nM normalized to 100%) from paired (C) human brown adipocytes (n = 8) or (D) inguinal beige adipocytes (n = 6) following 24 hr incubation with cortisol at 0, 100, and 1,000 nM. Cortisol (100 nM) increased UCP-1 levels in human brown adipocytes but decreased UCP-1 in murine adipocytes. (E) UCP-1 mRNA levels from paired primary human brown adipocytes (n = 7) following 24 hr incubation with cortisol at 0, 100, and 1,000 nM or equivalent concentrations (3.75 [pale red columns] and 37.5 nM [pale green columns]) of the selective glucocorticoid (GC) receptor (GR) agonist dexamethasone (Dex) ± the mineralocorticoid receptor (MR) antagonist eplerenone (10 μM). Low-dose cortisol and dexamethasone increased UCP-1 mRNA levels, while eplerenone did not alter UCP-1. (F) UCP-1 mRNA levels from paired interscapular murine brown adipocytes (n = 6) following 4 and 8 hr incubation with 0, 25 (yellow columns), 50 (orange columns), 100, and 1,000 nM cortisol. Low- and high-dose cortisol suppressed UCP-1 following 8 hr incubation. Data were analyzed by repeated-measures ANOVA with post hoc Bonferroni testing. ^∗^p < 0.05, ^∗∗^p < 0.01 versus 0 nM; $p < 0.05, $$p < 0.01 versus 100 nM cortisol.

To determine whether glucocorticoids enhance oxygen consumption by brown adipocytes in vitro, human brown and white adipocytes were cultured for 24 hr in 0, 100, or 1,000 nM cortisol. Oxygen consumption was measured on a Seahorse XFe24 analyzer. Cortisol at 100 nM increased basal and isoprenaline-stimulated respiration by brown adipocytes compared with 0 and 1000 nM cortisol (Figure 5A). Cortisol did not alter either basal or stimulated respiration in the white adipocytes (Figure S3A). Glucocorticoids did not alter the extracellular acidification rate in either brown or white adipocytes (data not shown). These results, in addition to confirming the in vivo findings, revealed that glucocorticoids enhance brown adipocyte activity directly.

To determine whether glucocorticoids enhance BAT activity by regulating UCP-1, human primary brown adipocytes were cultured for 24 hr in 0, 100, or 1,000 nM cortisol, as above. As before, UCP-1 levels were substantially increased in the brown adipocytes compared with the white adipocytes (>25-fold; data not shown). The 100 nM cortisol concentration stimulated UCP-1 mRNA levels compared with both 0 and 1,000 nM cortisol (Figure 5C). The effect of glucocorticoids on other mechanisms that could contribute to BAT activation was also examined. In parallel with the increased respiration and UCP-1 mRNA levels, 100 nM cortisol increased GLUT-4 mRNA levels in brown adipocytes (Figure 5C) but did not alter β3-AR, C/EBPβ, PPARα, CPT-1b, GLUT-1, or ZFP516 (the recently described upstream regulator of UCP-1; Dempersmier et al., 2015). High-dose cortisol (1,000 nM) increased PGC-1α mRNA levels in both brown and white adipocytes. In white adipocytes, cortisol did not alter mRNA levels of the other above genes (Figure S3B). Glucocorticoids did not alter mRNA levels of the mitochondrial markers cytochrome *c* and F1-ATPase or markers of adipocyte differentiation in brown adipocytes, indicating that glucocorticoids were not simply enhancing mitochondrial number and activity (Figures S3C and S3D). In addition, markers of adipocyte differentiation were similar between white and brown adipocytes (PPARγ 0.80 ± 0.14 [WAT] versus 0.96 ± 0.11 [BAT] a.u., FABP4 1.14 ± 0.32 [WAT] versus 0.87 ± 0.13 [BAT] a.u.).

To determine whether glucocorticoids increase UCP-1 via the glucocorticoid receptor (GR), human primary brown adipocytes were initially incubated with the GR antagonist RU38486. However, RU38486 substantially increased UCP-1 mRNA levels in glucocorticoid-free medium (1.6 ± 0.8 versus 0.4 ± 0.2 a.u., p < 0.05), as shown previously in murine brown adipocytes (Rodríguez and Palou, 2004), meaning RU38486 could not be used for this purpose. Therefore, human primary brown adipocytes were incubated for 24 hr with 0, 100, or 1,000 nM cortisol or equivalent doses of dexamethasone (a selective GR agonist ∼27-fold more potent than cortisol) in the presence or absence of the mineralocorticoid receptor antagonist eplerenone. Both 100 nM cortisol and 3.75 nM dexamethasone increased UCP-1 mRNA levels by ∼40% (Figure 5E), which was not reversed by eplerenone. These results confirmed that high physiological, but not supraphysiological, glucocorticoid concentrations increase UCP-1 and determined that this effect is most likely mediated through GR.

#### Contrasting Effects of Glucocorticoids in Human and Murine Brown and Beige Adipocytes

These findings that glucocorticoids enhance BAT activity in humans both in vivo and in vitro, most likely by increasing UCP-1, are at odds with consistent previous reports that glucocorticoids suppress BAT activation in rodents. Most of these studies have used supraphysiological concentrations of glucocorticoids for longer duration than in the human experiments above, and have usually examined only interscapular classic BAT, which may not be analogous with human BAT. To compare glucocorticoid regulation of BAT in humans and mice, we obtained the epididymal and inguinal WAT and interscapular BAT from the 129 mouse strain (which readily develop browning of their inguinal adipose depot; Wu et al., 2012) and cultured and differentiated the stromal vascular fractions as in the human cells. To induce browning of the inguinal adipocytes, following differentiation half of these cells were treated with the β3-adrenoreceptor agonist CL316,243 for 5 days (Figure S4A). Differentiated adipocytes were then incubated with 0, 100, or 1,000 nM cortisol for 24 hr, as above. In marked contrast to the human brown adipocytes, 100 and 1,000 nM cortisol suppressed UCP-1 mRNA levels by ∼80%–85% in inguinal (Figure 5D), CL316,243-treated inguinal, and interscapular brown adipocytes (Figure S4A–S4C). UCP-1 levels were extremely low in the epididymal WAT and were unchanged by cortisol. Cortisol increased PGC-1α and GLUT-4 mRNA levels in the inguinal adipocytes (Figure 5D) as in human brown adipocytes and decreased β3-AR, C/EBPβ, and GLUT-1 mRNA levels in the brown adipocytes (Figure S4C). Cortisol similarly induced classic glucocorticoid-regulated genes such as HSL, ATGL, and PER1 in both human and murine white and brown adipocytes (Figures S3C, S3D, and S4D–S4F), highlighting that cortisol was activating GR in both species. To determine whether glucocorticoids also reduced respiration in murine cells, basal and isoprenaline-stimulated oxygen consumption was measured in the murine beige and brown adipocytes incubated with cortisol for 24 hr as above. In contrast to the human brown adipocytes, 100 and 1,000 nM cortisol strongly suppressed isoprenaline-stimulated respiration in both inguinal (Figure 5B), CL316,243-treated inguinal, and brown adipocytes (Figures S5A and S5B).These results strongly suggested that glucocorticoid regulation of UCP-1 was species specific; however, to determine whether lower cortisol concentrations with shorter incubation periods could stimulate UCP-1, we incubated murine inguinal (unstimulated and CL316,243 treated) and brown adipocytes with either 0, 25, 50, 100, or 1,000 nM cortisol for both 4 and 8 hr. None of the cortisol concentrations increased UCP-1 mRNA levels in either the brown (Figure 5F) or beige adipocytes (Figures S4G and S4H) following 4 hr incubation, and by 8 hr even the lowest cortisol concentration suppressed UCP-1 in the brown adipocytes (Figure 5F).

### Chronic Glucocorticoid Excess Suppresses In Vivo BAT Activity in Humans

**Figure 6:**
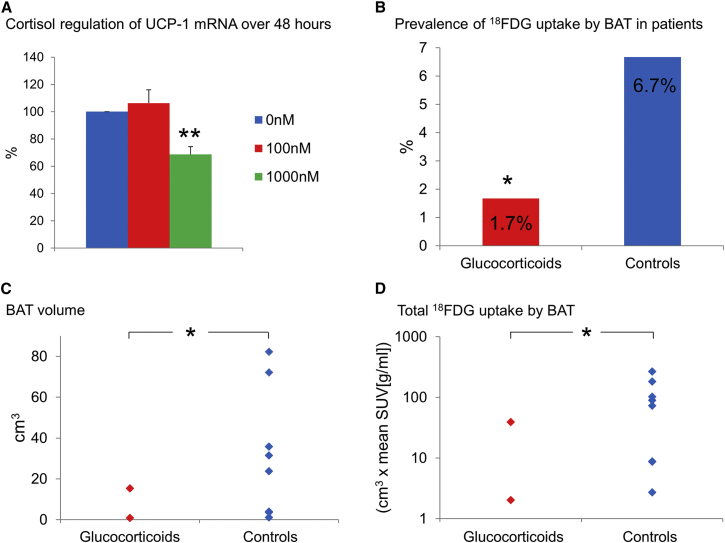
Chronic Glucocorticoid Use Suppresses BAT Activation in Humans (A) UCP-1 mRNA levels from paired human brown adipocytes (n = 9) following 48 hr incubation with either 0 (blue columns), 100 (red columns), or 1,000 nM (green columns) cortisol with 0 nM normalized to 100%. Data were analyzed by repeated-measures ANOVA with post hoc Bonferroni testing. Data are mean ± SEM. (B) The prevalence of ^18^FDG uptake by BAT (PET/CT scanning performed 1 hr following a 400 MBq ^18^FDG injection at room temperature [20°C–21°C]) in patients prescribed oral glucocorticoids (red column) or in matched controls (blue column). Data were analyzed by chi-square test. (C and D) (C) The volume of active BAT (^18^FDG uptake with SUV ≥ 2.0) and (D) the total ^18^FDG uptake by BAT in glucocorticoid treated (red diamonds) and control (blue diamonds) patients with detectable ^18^FDG uptake by BAT. BAT volume and total ^18^FDG uptake by BAT were reduced in glucocorticoid-treated patients. Data were analyzed using the Mann-Whitney U test. ^∗^p < 0.05, ^∗∗^p < 0.01 versus control/0 nM.

Chronic glucocorticoid excess causes obesity, so to determine whether this acute stimulatory effect on BAT activity was maintained over the longer term, primary human brown adipocytes were cultured in 0, 100, and 1,000 nM cortisol for 48 hr. The 100 nM cortisol concentration failed to increase UCP-1 following 48 hr incubation, while 1,000 nM cortisol substantially suppressed UCP-1 (Figure 6A). This suggested that the acute stimulatory effect on BAT activation was unlikely to be maintained more chronically. To test this further, we performed a retrospective analysis of all patients who had undergone PET/CT scanning in the Royal Infirmary of Edinburgh over the past 2 years. We identified 129 patients who had been taking oral glucocorticoids for at least 2 weeks at the time of their scan and 120 age-, sex-, BMI-, glucose-, and disease-matched controls with no history of any glucocorticoid use over the preceding year (Table S3). Ten of the 249 patients (4.0%) had detectable ^18^FDG uptake by BAT; these “BAT-positive” patients were younger (54.4 ± 6.2 versus 64.4 ± 1.0 years, p < 0.05) and had lower BMI (20.6 ± 0.7 versus 25.3 ± 0.3 kg/m^2^, p < 0.01) than the “BAT-negative” patients, in agreement with previous data (Saito et al., 2009, Cypess et al., 2009). Significantly fewer glucocorticoid-treated patients had detectable BAT compared to controls (Figure 6B). The controls had greater BAT volume and total ^18^FDG uptake by BAT than glucocorticoid-treated patients (Figures 6C and 6D). In contrast to acute glucocorticoid treatment, this shows that chronic glucocorticoid treatment suppresses BAT activation in humans.

## Discussion

This work shows that glucocorticoids acutely increase BAT activity in humans. In vivo, the glucocorticoid prednisolone increased ^18^FDG uptake by BAT, supraclavicular temperature, and cold-induced thermogenesis during cold exposure in healthy volunteers. Furthermore, at physiological concentrations the endogenous glucocorticoid cortisol increased basal and isoprenaline-stimulated respiration in human brown, but not white, adipocytes and increased UCP-1 mRNA levels, an effect most likely mediated through the glucocorticoid receptor. This was in marked contrast to rodent brown and beige adipocytes, where cortisol substantially suppressed isoprenaline-stimulated respiration and UCP-1. Lower dose and shorter cortisol incubations also failed to induce UCP-1 in the murine adipocytes, confirming the species-specific regulation of UCP-1 by glucocorticoids. These findings highlight the challenges of translating rodent findings into humans, particularly with regard to the new factors discovered to activate BAT or induce “browning” in rodents, which are being proposed as possible new therapeutic targets for human obesity (Sidossis and Kajimura, 2015). In addition, these findings highlight the importance of studying human BAT both in vivo and in vitro and suggest, at least in relation to regulation by glucocorticoids, that the difference between species is greater than any differences between beige and classic BAT. These findings also highlight the complex tissue-specific actions of glucocorticoids, decreasing whole-body glucose uptake (by inducing insulin resistance in tissues such as muscle) but increasing glucose uptake by BAT. Whole-body glucose uptake was not increased during cold exposure, signifying that the increased glucose uptake by BAT was insufficient to impact on whole-body glucose utilization at least during the first hour of cold exposure.

Only one previous study has examined regulation of human BAT by glucocorticoids (Barclay et al., 2015), demonstrating only in vitro that the potent glucocorticoid dexamethasone suppressed isoprenaline-stimulated UCP-1 levels and oxygen consumption. However, the glucocorticoid concentrations used were 30- and 300-fold higher in potency than those in the current in vitro experiments. Since we observed that 100 nM, but not 1,000 nM, cortisol stimulated BAT activity, it is possible that even higher glucocorticoid concentrations would have suppressed BAT activity in vitro. Endogenous cortisol levels in adipose tissue are approximately 25 nM (Hughes et al., 2010), so levels of 100 nM in adipose tissue are likely to be physiologically relevant during stress. In addition, 20 mg of prednisolone per day (which is ∼4-fold more potent than cortisol), as used in our in vivo studies, would increase glucocorticoid exposure ∼4- to 5-fold higher than in normal health (Kraan et al., 1998), which is equivalent to cortisol production during severe stress (Boonen et al., 2013). Therefore, high physiological glucocorticoid levels for 24 hr both in vivo and in vitro induce BAT activation.

It appears that glucocorticoid-induced thermogenesis represents a physiological response in humans. While cortisol concentrations during placebo administration did not rise during cold exposure in this study or others (Ouellet et al., 2012), cortisol did not continue to fall, as would be expected from normal diurnal variation. Severe cold exposure sufficient to induce shivering does increase cortisol secretion (Wilson et al., 1970). We can speculate that during severe cold exposure (or, indeed, in combination with enhanced adrenaline secretion in the fight/flight response), the increased cortisol secretion enhances BAT thermogenesis to protect core body temperature. Furthermore, recent work has determined that there is a circadian rhythm to BAT thermogenesis in humans with elevated BAT function coinciding with peak cortisol secretion early in the morning, so it is plausible that cortisol may in part drive this circadian rhythm (Lee et al., 2016).

The value of infrared thermography to measure BAT activity is debated due to potential confounders such as heat production from other tissues and changes in overlying skin blood flow; however, our results using this technique were similar to those obtained using PET/CT, suggesting that infrared thermography can be used to detect cold-induced BAT activation. Importantly, we minimized potential confounders by ensuring that subjects wore identical clothing and that room temperatures were similar between visits. Furthermore, it is unclear whether infrared thermography data should be presented as absolute supraclavicular skin temperatures or as the temperature gradient between the supraclavicular skin and anterior chest (Jang et al., 2014). However, our results were similar when calculated using the temperature gradient (data not shown) because the chest temperature did not change between phases.

In our study, the increased levels of UCP-1 were not maintained following 48 hr of glucocorticoid treatment, while our chronic PET/CT data reveal that chronic glucocorticoid excess in fact suppresses BAT activation. While acute elevations of cortisol represent an adaptive stress response, chronic glucocorticoid excess is maladaptive and results in weight gain, insulin resistance, and dyslipidemia (Macfarlane et al., 2008). Therefore, suppression of BAT activation potentially contributes to these adverse metabolic consequences of chronic glucocorticoid treatment. It is also interesting to speculate how our findings relate to the reduced BAT mass/activation observed in obesity (van Marken Lichtenbelt et al., 2009). The majority of studies find peak plasma cortisol levels to be reduced in obesity (Rask et al., 2002, Strain et al., 1982, Phillips et al., 2000), likely due to increased cortisol clearance by the hepatic A-ring reductases (Andrew et al., 1998), which could result in reduced BAT cortisol concentrations, leading to reduced thermogenesis. In addition, we found a positive correlation between the cortisol-inactivating enzyme 11β-HSD2 and BMI in human BAT; however, 11β-HSD2 levels in both WAT and BAT are low, and it is unclear whether this would substantially reduce cortisol concentrations in BAT in vivo. An unexpected finding in this study was the significantly elevated 11β-HSD1 and reduced 11β-HSD2 mRNA levels seen in the brown adipocytes compared with the white adipocytes. These differences were not observed in the whole tissue, so this may be caused by increased sensitivity in the brown adipocytes to unknown factors in the culture medium, such as macronutrients that can induce 11β-HSD1 (Stimson et al., 2014) or glucocorticoids that can suppress 11β-HSD2 (Lee et al., 2008).

We examined several genes known to be important in BAT function to determine the likely cause of the increased thermogenesis and the species-specific regulation by glucocorticoids. While glucocorticoids increased PGC-1α and GLUT-4 levels in human brown adipocytes, these were also induced in murine inguinal adipocytes and so are not responsible for these differences in BAT function. Mitochondrial markers were not increased, indicating that glucocorticoids likely do not improve general mitochondrial activity. However, the clear differential regulation of the key thermogenic protein UCP-1 between human and murine cells that importantly paralleled the differences in isoprenaline-induced respiration likely explains the clear differences between species, although the reason for this differential regulation is unclear. Well-known glucocorticoid-regulated genes such as HSL, ATGL, and PER1 were induced in both murine and human adipocytes, meaning that glucocorticoids were activating the GR signaling pathway in both species. GR binding in BAT has not been examined to date, so it is unclear if GR binds directly to the UCP-1 promoter region to enhance transcription. It appears unlikely that this effect is mediated through ZFP516, as this was unaltered in both species. In addition, prednisolone increased NEFA concentrations, which may indirectly enhance UCP-1 activation in BAT (Shabalina et al., 2004); however, ATGL and HSL (key genes in lipolysis) were increased in both species, while in vitro only 1,000 nM cortisol increased glycerol release (a marker of lipolysis) in the medium (data not shown) without increasing isoprenaline-stimulated oxygen consumption, so it is unlikely that this mechanism drives the increased BAT activation. Although the reason for the differential regulation of UCP-1 between species is unclear, ACTH (which rises during normal physiological stress) increases BAT activation in rodents (van den Beukel et al., 2014). This may partially counteract the suppressive effect of glucocorticoids, while acute stress enhances BAT thermogenesis in rats (Kataoka et al., 2014).

To conclude, we have shown that glucocorticoids acutely increase BAT activity in humans at least in part by activating UCP-1. This study highlights important species-specific differences in the regulation of BAT activation that may have important implications for the translation of novel therapeutic strategies designed to activate BAT to improve metabolic health.

## Experimental Procedures

### Protocols for In Vivo Studies of BAT Activity

#### Glucocorticoid Regulation of Cold-Induced ^18^FDG Uptake by Human BAT

Six men were recruited to a double-blind, randomized crossover study. Inclusion criteria were as follows: aged 18–35 years, BMI 18.5–25 kg/m^2^, no acute or chronic medical conditions, on no regular medications, alcohol intake ≤21 units per week, no claustrophobia, and normal screening blood tests (full blood count, glucose, kidney, liver, and thyroid function). Participants were randomized to receive three doses of 10 mg prednisolone or placebo 12 hr apart prior to each study visit (at 0800 hr and 2000 hr the day prior to each study visit and at 0800 hr on the morning of the study visit). Volunteers were instructed to avoid alcohol or exercise for 48 hr prior to each visit. Volunteers attended the Clinical Research Facility at the Royal Infirmary of Edinburgh after overnight fast in standard light clothing and wore identical clothing at each visit. Subjects were placed in a room at 23°C–24°C (warm room), and measurements were performed of height, weight, fat mass, and blood pressure, and fasting blood samples were collected for glucose, insulin, ACTH, cortisol, and NEFAs.

An infusion of 6,6-[^2^H]_2_-glucose was commenced at 0.22 μmol/kg/min for 180 min following an initial bolus of 17.6 μmol/kg (Figure 1A). Volunteers then remained at rest in the warm room for 2 hr. Thereafter, subjects were transferred to the Clinical Research Imaging Centre and were placed supine in a room cooled to 17°C (cold room) for 2 hr. Subjects were checked every 15 min for signs or symptoms of shivering. Following 1 hr in the cold room, subjects were given an intravenous injection of 75 MBq ^18^FDG. The PET scan commenced 1 hr following the ^18^FDG injection, after a CT scan for attenuation correction. Subjects were then allowed to return home and attended for a second visit after at least 2 weeks washout. All studies involving human participants were reviewed and approved by the South East Scotland Research Ethics Committee, and informed consent was obtained from all participants.

#### Glucocorticoid Regulation of Cold-Induced Thermogenesis and Heat Production by Human BAT

Nine lean, healthy subjects (four female, five male) were recruited to a double-blind, randomized crossover study. Inclusion criteria were identical to the PET study except for additional inclusion criteria for the female participants: no current pregnancy and alcohol intake ≤14 units per week. Participants were randomized to receive identical dosing as in the PET study, taking three doses of 10 mg prednisolone or placebo 12 hr apart prior to each study visit, and the study protocol was identical to the PET study except for the following differences. Subjects were placed on a bed in a warm room (23°C–24°C) at t = 0 min for the first 2 hr (Figure 1B). At t + 110 min, subjects placed their hands in cold water (15°C) in an attempt to activate their BAT (Symonds et al., 2012). At t + 120 min, volunteers were moved to a room cooled to 16°C (cold room) for a further 2 hr to more robustly activate their BAT. Every 20 min from t = 0 onward, each subject’s peripheral temperature was measured using a temperature probe applied to the dorsum of the hand (YSI 409 series, Henleys Medical Supplies Ltd), while thermal imaging was performed of the neck and upper body region. Energy expenditure was measured each hour using indirect calorimetry. At the end of the 4 hr protocol, subjects were allowed to return home and attended for their second visit either 2 weeks (in males) or 4 weeks (in females, to ensure menstrual cycle in the same phase at each visit) later to allow adequate washout between phases.

#### PET/CT Scanning Protocol and Analysis

All subjects were placed supine in a hybrid PET/CT scanner (Biograph mCT, Siemens Medical Systems). Subjects underwent an initial low-dose CT for attenuation correction (non-enhanced, 120 kV) with tube current modulation applied (20 [in healthy volunteers] or 50 [in patients] mAs quality reference) followed by static PET imaging of the upper body using 10 (volunteers) or 3 (patients) min beds. Images were analyzed using PMOD version 3.409 (PMOD technologies). ^18^FDG uptake by BAT was quantified by measuring the mean standard uptake value (SUV) from all pixels with an SUV of greater than 2-fold background (>2.0), which corresponded to tissues with a radio density on the CT scan with Hounsfield unit (HU) values within the expected range for adipose tissue (from −150 to −30 HU). The total ^18^FDG uptake by BAT was calculated as the mean SUV multiplied by the volume of active BAT.

#### Thermal Imaging

Thermal imaging was performed using a FLIR T650sc infrared camera. The camera was placed 1 m from the subject and the subject’s upper body was photographed at the intervals described in Figure 1B. Identically sized regions of interest were drawn around the right and left supraclavicular regions and the anterior chest as shown in Figure 3A using Research IR version 4 (FLIR). The mean and maximum supraclavicular (left and right) and chest temperatures were recorded from each image, and the mean values from all images taken during each condition (warm, cold water, and cold exposure) are presented in the results. The results using the mean of the mean and the mean of the maximum were similar; as such, only the mean of the mean is presented.

#### Indirect Calorimetry

Energy expenditure was measured for 15 min each hour using a ventilated-hood indirect calorimeter (GEM Nutrition). The first 5 min of data were discarded and the mean value for the final 10 min recorded each hour. Energy expenditure (EE) is presented as the mean of two values obtained during warm and cold exposure. Cold-induced thermogenesis was calculated by subtracting the mean EE in the cold room from the EE in the warm room.

#### Glucose Kinetics

Endogenous and 6,6-[^2^H]_2_-glucose were measured using liquid chromatography-mass spectrometry as previously described (Macfarlane et al., 2014). The mean concentration of D2-glucose at room temperature (from t + 90 to t + 120 min) and during the first hour of cold exposure (from t + 150 to 180 min) was used to calculate the metabolic clearance rate (MCR) of glucose using the equation MCR = D2-glucose infusion rate/ [mean D2-glucose].

#### Biochemical Assays

ACTH, insulin (DRG Instruments), and noradrenaline concentrations (LDN) were measured using commercially available ELISA kits. NEFAs were measured using a colorimetric assay (Wako Diagnostics). Cortisol was measured by liquid chromatography-mass spectrometry as previously described (Stimson et al., 2009).

### In Vitro Studies of BAT Activity

Detailed in the Supplemental Information.

### Statistical Analysis

Data are presented as mean ± SEM. Comparisons between two related groups were examined using the paired t test for normally distributed data, and using the Wilcoxon signed-rank test for data not normally distributed. Comparisons involving three or more groups were analyzed using repeated-measures ANOVA with post hoc testing using Bonferroni correction. Associations were tested using Pearson’s correlation coefficient with Bonferroni correction. Data were tested for normal distribution using the one-sample Kolmogorov-Smirnov test. p < 0.05 was considered significant. Data were analyzed using SPSS version 19.

## Author Contributions

R.H.S. designed the studies in conjunction with L.E.R., M.A., A.M.F., E.J.R.B., N.M.M., and B.R.W. L.E.R., M.A., A.M.F., J.F., M.N., R.N.C., N.M.M., and R.H.S. conducted the experiments and/or analyzed data. R.H.S. wrote the initial draft of the manuscript, and all authors critically reviewed the manuscript.

## Figures and Tables

**Table 1:** Anthropometric and Biochemical Measurements in In Vivo Studies

	PET/CT Study		Thermal Imaging Study	
Prednisolone	Placebo	Prednisolone	Placebo
No. of participants (male/female)	6/0		5/4	
Age (years)	22.1 ± 1.2	22.1 ± 1.2	22.7 ± 1.3	22.7 ± 1.3
Weight (kg)	68.7 ± 3.1	68.6 ± 3.1	65.3 ± 2.9	65.3 ± 2.8
BMI (kg/m^2^)	22.0 ± 0.9	22.0 ± 0.9	21.8 ± 0.6	21.8 ± 0.6
Fat mass (kg)	8.6 ± 0.9	8.4 ± 0.9	12.5 ± 1.2	12.4 ± 1.1
Systolic BP (mmHg)	128 ± 5	123 ± 5	116 ± 3	114 ± 2
Diastolic BP (mmHg)	70 ± 2	69 ± 1	66 ± 2	66 ± 3
Warm environmental temperature (°C)	23.8 ± 0.2	23.5 ± 0.1	23.7 ± 0.3	24.0 ± 0.3
Cold environmental temperature (°C)	17.5 ± 0.2^###^	17.2 ± 0.4^###^	16.5 ± 0.2^###^	16.2 ± 0.1^###^

**Fasting Biochemistry**				

Glucose (mmol/L)	6.0 ± 0.3^∗^	5.3 ± 0.3	5.0 ± 0.1^∗^	4.4 ± 0.1
Insulin (pmol/L)	66 ± 11^∗^	40 ± 7	56 ± 7^∗∗^	28 ± 5
Total cholesterol (mmol/L)	3.8 ± 0.2	3.8 ± 0.2	4.1 ± 0.2	4.1 ± 0.2
Triglycerides (mmol/L)	0.6 ± 0.0	0.7 ± 0.1	0.8 ± 0.1	0.8 ± 0.1
NEFAs (warm room) (μmol/L)	558 ± 82^∗^	362 ± 50	351 ± 53	244 ± 53
NEFAs (cold room) (μmol/L)	515 ± 66	391 ± 34	453 ± 54^∗^	277 ± 71
ACTH (warm room) (ng/L)	ND^∗∗^	47 ± 6	ND^∗∗^	49 ± 8
ACTH (cold room) (ng/L)	ND^∗∗^	22 ± 6^##^	ND^∗∗^	26 ± 3^#^
Noradrenaline (warm room) (ng/L)	305 ± 74	304 ± 82	492 ± 66	612 ± 86
Noradrenaline (cold room) (ng/L)	451 ± 120	575 ± 141	564 ± 102	611 ± 123

Data are mean ± SEM. ND, not detected. ^∗^p < 0.05, ^∗∗^p < 0.01 versus placebo. ^#^p < 0.05, ^##^p < 0.01, ^###^p < 0.001 versus measurements in warm room.

## Supplemental Information

Document S1. Supplemental Experimental Procedures, Figures S1–S5, and Tables S1–S4

Document S2. Article plus Supplemental Information
