# Supplementary material for: Glucocorticoids Acutely Increase Brown Adipose Tissue Activity in Humans, Revealing Species-Specific Differences in UCP-1 Regulation
Source: Cell Metab. 2016 Jul 12;24(1):130–41. doi: 10.1016/j.cmet.2016.06.011 (PMC4949380; doi:10.1016/j.cmet.2016.06.011)
Supplement: Document S1. Supplemental Experimental Procedures, Figures S1–S5, and Tables S1–S4 [file mmc1.pdf]

Cell Metabolism, Volume *24*

Supplemental Information

Glucocorticoids Acutely Increase Brown Adipose Tissue Activity in Humans, Revealing Species-Specific Differences in UCP-1 Regulation

Lynne E. Ramage, Murat Akyol, Alison M. Fletcher, John Forsythe, Mark Nixon, Roderick N. Carter, Edwin J.R. van Beek, Nicholas M. Morton, Brian R. Walker, and Roland H. Stimson

Supplemental Information

Figure S1 (related to Experimental Procedures) Anatomical location of intra-operative collection of human brown adipose tissue

A) CT and B) PET/CT fused axial images of the neck from a healthy subject. Intra-operative samples of brown adipose tissue were obtained from the deep supraclavicular region posterior to the lateral thyroid and adjacent to the longus colli muscle, highlighted by the arrow in panel B identifying the adipose tissue with substantial cold-induced ^{18}fluorodeoxyglucose uptake (blue/yellow areas) in that site. Paired white adipose tissue samples were simultaneously obtained from the superficial adipose tissue in the neck.

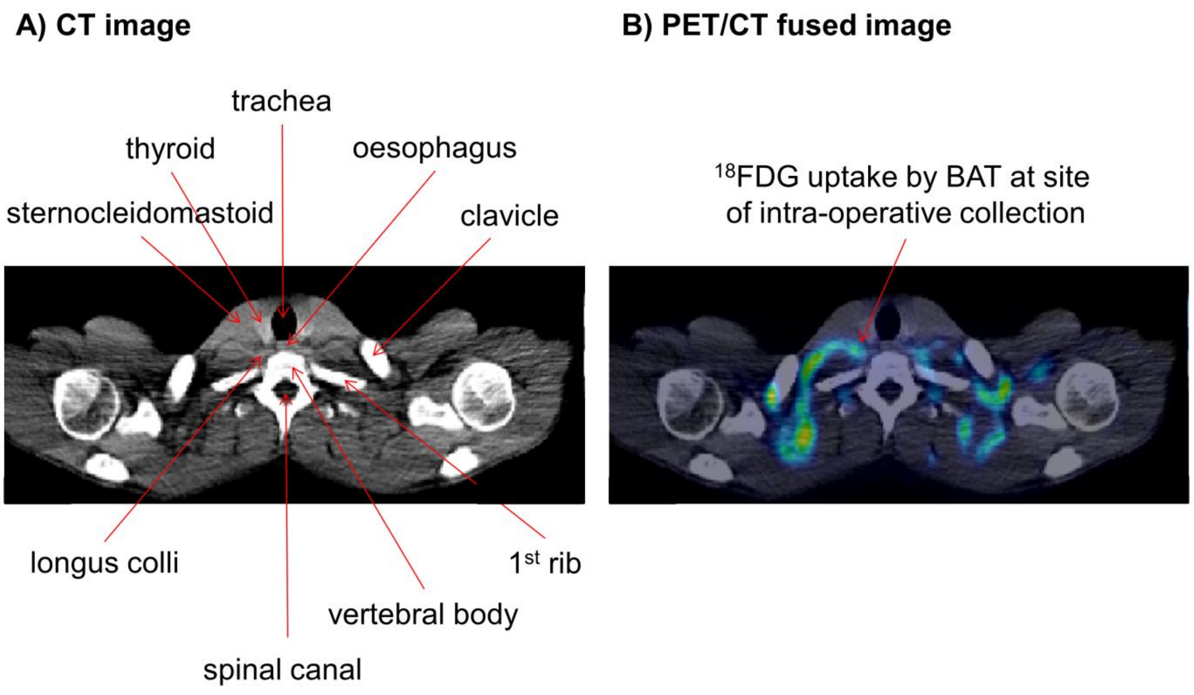

Figure S2 (related to Figure 4) Human BAT expresses markers of both beige and classic BAT

*Data are mean ± SEM (n=11) for mRNA levels from differentiated human pre-adipocytes cultured from the superficial (WAT, yellow columns) and the deeper (BAT, red columns) depots. The brown adipocytes expressed higher levels of the classic BAT markers Ebf3 and ZIC1 than the white adipocytes. In addition, the brown adipocytes had increased levels of the beige markers TMEM26 and Tbx1 but lower levels of HOXC9. The brown adipocytes tended to have lower levels of the WAT marker leptin than the white adipocytes (p=0.07). *P<0.05, **P<0.01 vs WAT.*

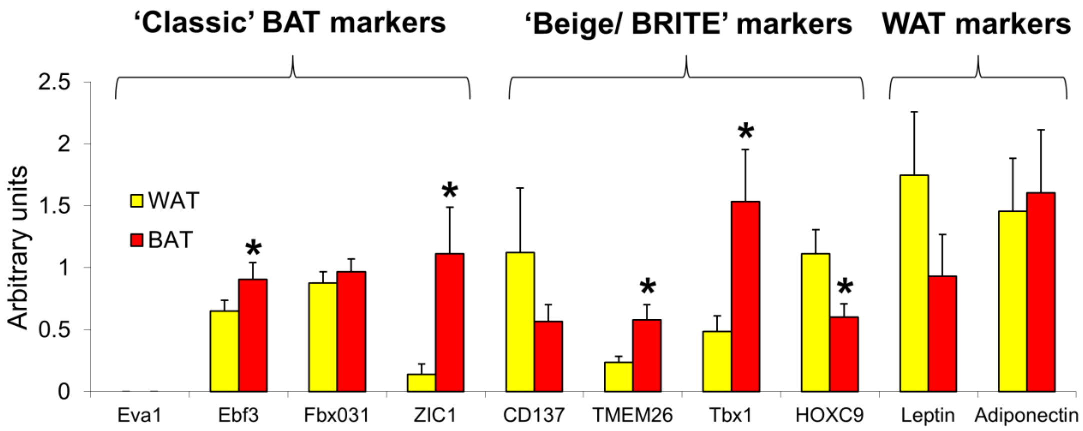

Figure S3 (related to Figure 5) Glucocorticoid regulation of human adipocytes

*A) Data are mean ± SEM for n=6 paired human white adipocytes cultured for 24 hours in either 0 (blue columns), 100 (red columns) or 1000nM (green columns) cortisol, with basal 0nM normalised to 100%. Cortisol did not alter either basal or isoprenaline-stimulated respiration. B-D) mRNA levels (with 0nM normalised to 100%) from paired B,D) white and C) brown adipocytes (n=8) following 24 hours incubation with cortisol 0, 100 and 1000nM. B) Cortisol did not alter UCP-1 transcript levels in the white adipocytes. mRNA levels of β3-adrenoreceptor (β3-AR) were below the limit of detection in the white adipocytes. Cortisol increased PGC-1α in white adipocytes and C,D) increased classic glucocorticoid (GC)-regulated genes such as hormone sensitive lipase (HSL), adipose triglyceride lipase (ATGL) and Per1 in brown and white adipocytes. *P<0.05, **P<0.01 vs 0nM.*

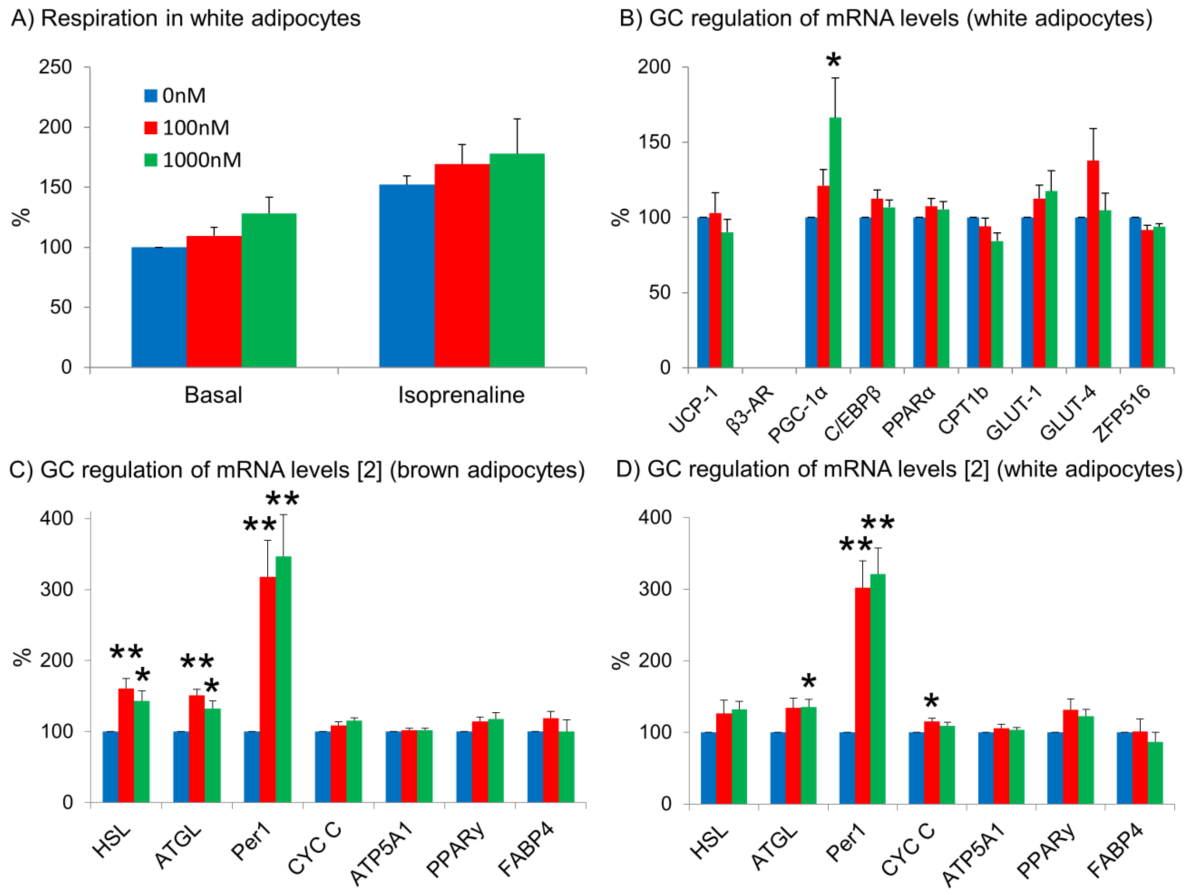

Figure S4 (related to Figure 5) Glucocorticoid regulation of murine adipocytes

*A) Data mean ± SEM for mRNA levels from paired primary murine adipocytes from the epididymal (Epi, n=4), inguinal (Ing, n=6) and interscapular brown adipose tissue (iBAT, n=6) depots following 24 hours incubation with the glucocorticoid (GC) cortisol at 0 (blue columns), 100 (red columns) and 1000 nM (green columns). CL316,243 (1µM) was added to inguinal cells (Ing+CL) for 5 days following differentiation to induce browning. B-F) mRNA levels (with 0nM normalised to 100%) from murine B,D,E) inguinal (n=6) and C,F) interscapular brown adipocytes (n=6) following 24 hours incubation with 0, 100 and 1000nM cortisol. G,H) UCP-1 mRNA levels from paired murine G) unstimulated (n=7) and H) CL316,243 treated (n=7) inguinal adipocytes following 4 and 8 hours incubation with 0, 25 (yellow columns), 50 (orange columns), 100 and 1000nM cortisol. Data were analysed by repeated measures ANOVA with post-hoc Bonferroni correction. *P<0.05, **P<0.01 vs 0nM; $P<0.05 vs 100nM cortisol.*

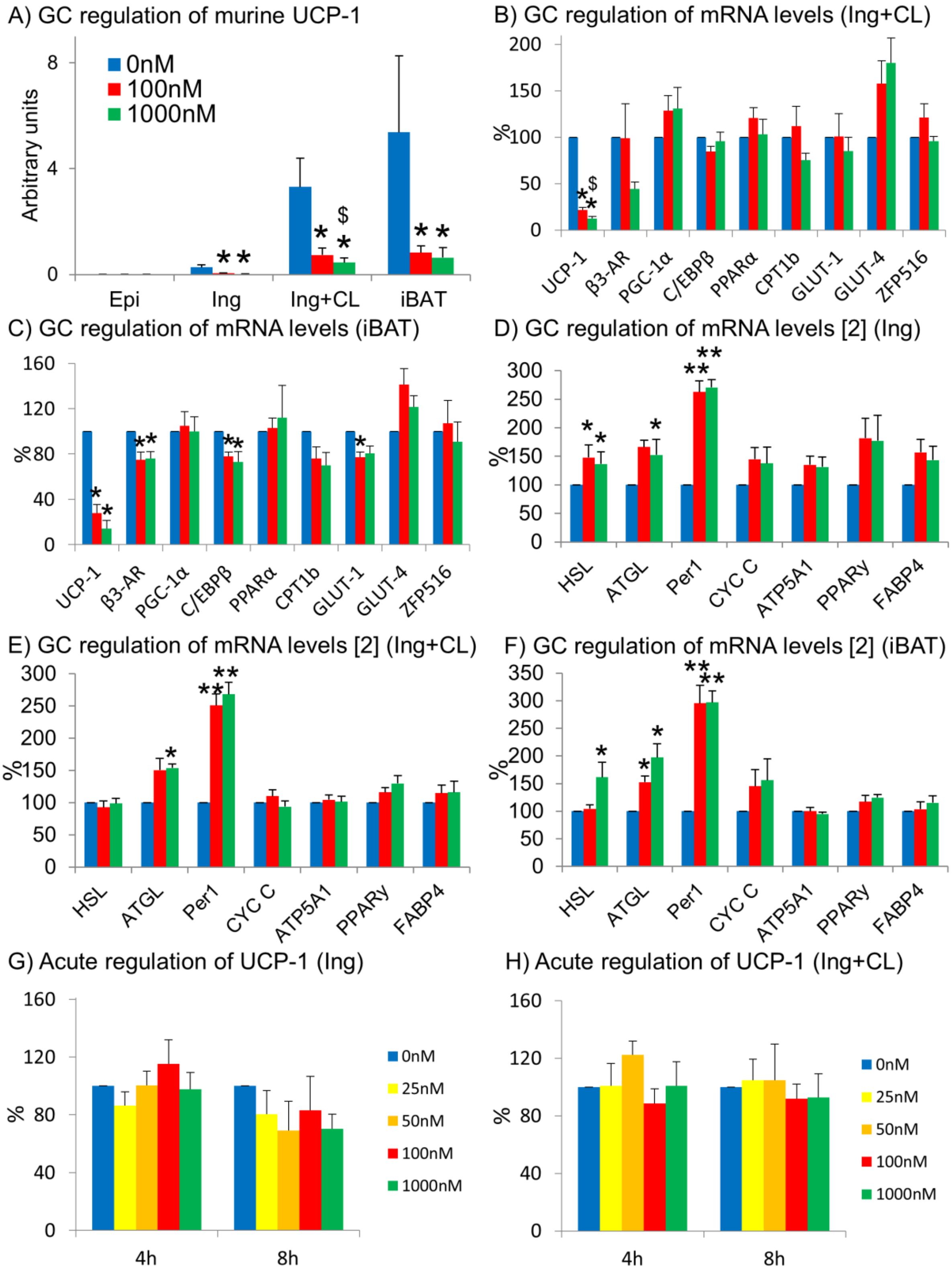
A) GC regulation of murine UCP-1
Arbitrary units
0nM
100nM
1000nM
8
4
0
Epi
Ing
Ing+CL
iBAT
$
B) GC regulation of mRNA levels (Ing+CL)
200
150
%100
50
0
UCP-1
β3-AR
PGC-1α
C/EBPβ
PPARα
CPT1b
GLUT-1
GLUT-4
ZFP516
C) GC regulation of mRNA levels (iBAT)
160
120
%80
40
0
D) GC regulation of mRNA levels [2] (Ing)
300
250
200
%150
100
50
0
HSL
ATGL
Per1
CYC C
ATP5A1
PPARγ
FABP4
E) GC regulation of mRNA levels [2] (Ing+CL)
F) GC regulation of mRNA levels [2] (iBAT)
350
G) Acute regulation of UCP-1 (Ing)
H) Acute regulation of UCP-1 (Ing+CL)
%
0nM
25nM
50nM
100nM
1000nM
4h
8h

Figure S5 (related to Figure 5) Glucocorticoid regulation of respiration

*A,B) Data are mean ± SEM (with basal 0nM normalised to 100%) for paired murine A) inguinal CL316,243 treated (n=7) and B) interscapular brown adipocytes (n=7) incubated for 24 hours with either 0 (blue columns), 100 (red columns) or 1000nM (green columns) cortisol. Cortisol (100nM and 1000nM) decreased isoprenaline-stimulated oxygen consumption in the murine adipocytes. Data were analysed by repeated measures ANOVA with post-hoc Bonferroni testing. *P<0.05 vs 0nM. C,D) Representative respirometry traces from C) human white (dotted lines) and brown (solid lines) and D) murine inguinal 'beige' adipocytes following incubation for 24 hours with either 0 (blue lines), 100 (red lines) or 1000nM (green lines) cortisol. Samples were all analysed in triplicate.*

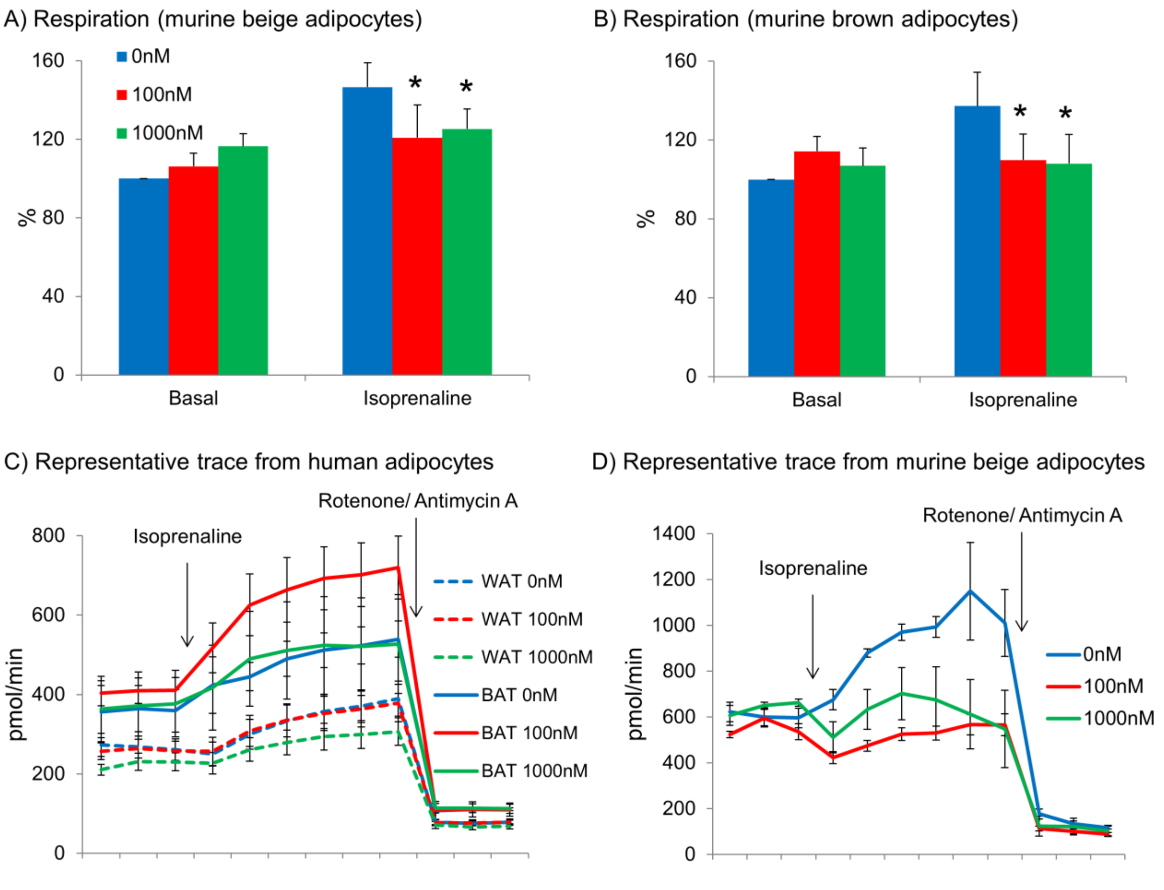

Table S1 (related to Experimental Procedures) Average outdoor environmental temperatures

Data are the mean maximum and minimum outdoor temperatures during the months when the two in vivo human studies were performed. Historical data were obtained from the Met Office Leuchars station which is situated closest to Edinburgh, Scotland, United Kingdom (1).

	**Maximum temperature (˚C)**	**Minimum temperature (˚C)**
**PET/CT study**		
March	13.3	3.7
April	10.1	2.7
May	12.5	5.3
June	14.5	8.6
July	17.1	10.3
August	18.6	11.3
September	16.2	8.0
**Thermal imaging study**		
January	6.4	0.3
February	7.8	0.5
March	9.7	2.1
April	13.3	2.7
May	14.0	5.0

Table S2 (related to Experimental Procedures) Participants for human in vitro experiments

Experiment/ Subject number	Operation	Underlying diagnosis	Age	Sex	BMI (kg/m^2)
**Whole tissue mRNA and histology**					
**1**	Parathyroidectomy	Primary hyperparathyroidism	52	Female	27.0
**2**	Parathyroidectomy	Primary hyperparathyroidism	70	Female	28.3
**3**	Parathyroidectomy	Primary hyperparathyroidism	63	Female	33.2
**4**	Thyroid lobectomy	Papillary carcinoma of thyroid	45	Female	26.5
**5**	Thyroid lobectomy	Hashimoto's thyroiditis	51	Female	30.7
**6**	Parathyroidectomy	Primary hyperparathyroidism	52	Female	47.1
**7**	Thyroid lobectomy	Follicular adenoma of thyroid	48	Female	17.6
**8**	Parathyroidectomy	Primary hyperparathyroidism	54	Male	33.3
**9**	Thyroid lobectomy	Benign hyperplastic thyroid nodule	39	Female	27.7
**Additional whole tissue for correlations**					
**1**	Thyroidectomy	Papillary carcinoma of thyroid	52	Female	39.4
**2**	Parathyroidectomy	Primary hyperparathyroidism	24	Male	27.7
**3**	Thyroid lobectomy	Benign hyperplastic nodule	46	Female	40.1
**4**	Thyroidectomy	Graves' disease	27	Female	30.1
**5**	Thyroid lobectomy	Multinodular goitre	37	Female	38.5
**6**	Thyroidectomy	Graves' disease	48	Female	32.3
**7**	Thyroid lobectomy	Follicular adenoma	27	Female	23.9
**8**	Thyroidectomy	Graves' disease	57	Female	25.8
**9**	Thyroid lobectomy	Multinodular goitre	66	Female	24.5
**10**	Thyroidectomy	Graves' disease	26	Female	20.9
**11**	Thyroidectomy	Graves' disease	36	Female	28.2
**12**	Parathyroidectomy	Primary hyperparathyroidism	62	Female	28.4
**Cultured adipocytes for mRNA and histology**					
**1**	Thyroidectomy	Graves' disease	21	Female	26.7
**2**	Thyroidectomy	Benign hyperplastic nodule	39	Female	27.8
**3**	Thyroid lobectomy	Benign hyperplastic nodule	41	Female	23.1
**4**	Parathyroidectomy	Primary hyperparathyroidism	57	Female	30.8
**5**	Parathyroidectomy	Primary hyperparathyroidism	66	Female	32.4
**6**	Thyroidectomy	Multinodular goitre	49	Male	33.2
**7**	Parathyroidectomy	Primary hyperparathyroidism	67	Female	32.0
**8**	Thyroid lobectomy	Benign hyperplastic nodule	55	Female	29.3
**9**	Parathyroidectomy	Primary hyperparathyroidism	34	Female	31.0
**10**	Parathyroidectomy	Primary hyperparathyroidism	58	Female	44.1
**11**	Parathyroidectomy	Primary hyperparathyroidism	42	Male	23.1

**Respirometry**					
**1**	Thyroid lobectomy	Toxic thyroid nodule	30	Male	20.2
**2**	Thyroid lobectomy	Follicular thyroid carcinoma	35	Male	38.9
**3**	Thyroidectomy	Graves' disease	25	Female	18.9
**4**	Parathyroidectomy	Primary hyperparathyroidism	48	Female	28.0
**5**	Thyroidectomy	Multinodular goitre	41	Female	45.7
**6**	Thyroidectomy	Graves' disease	31	Female	37.8
**24 hour incubation with glucocorticoids**					
**1**	Thyroid lobectomy	Benign thyroid nodule	41	Female	27.5
**2**	Parathyroidectomy	Hyperparathyroidism	59	Female	24.5
**3**	Thyroidectomy	Papillary thyroid carcinoma	52	Female	39.4
**4**	Thyroid lobectomy	Toxic thyroid adenoma	30	Male	20.2
**5**	Thyroidectomy	Toxic multinodular goitre	50	Female	22.3
**6**	Parathyroidectomy	Hyperparathyroidism	39	Female	31.7
**7**	Parathyroidectomy	Hyperparathyroidism	44	Male	37.6
**8**	Thyroid lobectomy	Follicular adenoma of thyroid	43	Male	26.2
**24 hour incubation +/- eplerenone**					
**1**	Thyroid lobectomy	Hyperplastic thyroid nodule	22	Female	28.7
**2**	Thyroidectomy	Graves' disease	42	Female	38.4
**3**	Thyroidectomy	Multinodular goitre	51	Female	36.1
**4**	Thyroid lobectomy	Colloid cyst	30	Female	25.4
**5**	Thyroidectomy	Multinodular goitre	57	Female	31.1
**6**	Parathyroidectomy	Hyperparathyroidism	58	Female	23.8
**7**	Thyroidectomy	Papillary thyroid carcinoma	31	Female	33.8
**48 hour incubation with glucocorticoids**					
**1**	Thyroid lobectomy	Normal thyroid	26	Female	25.4
**2**	Thyroid lobectomy	Multinodular goitre	48	Female	32.9
**3**	Thyroidectomy	Follicular carcinoma of thyroid	50	Female	26.0
**4**	Thyroidectomy	Papillary thyroid carcinoma	23	Female	20.9
**5**	Thyroid lobectomy	Hurthle cell adenoma	32	Female	20.3
**6**	Thyroid lobectomy	Follicular adenoma of thyroid	24	Male	21.6
**7**	Thyroidectomy	Multinodular goitre	22	Male	35.3
**8**	Thyroid lobectomy	Follicular adenoma of thyroid	45	Female	22.5
**9**	Thyroidectomy	Multinodular goitre	48	Female	31.1

Table S3 (related to Figure 6) Patient characteristics for retrospective analysis of PET/CT scans

Data are mean ± SEM. Anthropometric and biochemical data from patients who attended the Royal Infirmary of Edinburgh to undergo PET/CT scanning as part of their diagnostic pathway. All patients received 400MBq of ^{18}FDG 60 minutes prior to undergoing PET/CT scanning of their body. Patients were kept in a room at 20-21˚C prior to and following the ^{18}FDG injection until the PET/CT scan was performed. Data were analysed using the unpaired t test.

	**Controls**	**Glucocorticoid treated**
**Number of patients**	120	129
**Age (years)**	64.5 ± 1.4	63.5 ± 1.4
**Sex (female/ male)**	66/ 54	67/ 62
**Weight (kg)**	70.2 ± 1.3	69.7 ± 1.5
**Body mass index (kg/m^2)**	25.2 ± 0.4	25.0 ± 0.5
**Fasting plasma glucose (mmol/L)**	6.0 ± 0.1	5.9 ± 0.1

Table S4 (related to Experimental Procedures) Primer sequences for qPCR and corresponding probe numbers

All assays were performed using the following primer probe sequences and the Roche probe library, except for β3-adrenoreceptor which was measured using the Taqman assay ID Hs00609046_m1, while hormone sensitive lipase (LIPE) was measured using the SYBR green assay and the forward primer ggaagtgctatcgtctctgg and reverse primer ggcagtcagtggcatctc.

Gene Name	Primer sequences 5' to 3'	Roche UPL Probe number
**Human samples**		
UCP1	F: ctcaccgcagggaaagaa<br>R: ggttgcccaatgaatactgc	25
PRDM16	F: tggctgcttctggactca<br>R: atattatttacaacgtcaccgtcact	63
PPARGC1A<br>(PGC-1α)	F: tgagagggccaagcaaag<br>R: ataaatcacacggcgctctt	13
DIO2<br>(de-iodinase type 2)	F: ggaagagcttcctcctcgat<br>R: tccttctgtactggagacatgc	47
NR3C1<br>(glucocorticoid receptor-α)	F: ttttcttcaaaagagcagtgga<br>R: gcatgctgggcagttttt	11
NR3C2<br>(mineralocorticoid receptor)	F: catcatgaaagttttgctgctact<br>R: tctttgatgtaatttgtcctcatttc	64
HSD11B1<br>(11β-HSD1)	F: caatggaagcattgttgtcg<br>R: ggcagcaaccattggataag	20
HSD11B2<br>(11β-HSD2)	F: gtcaaggtcagcatcatcca<br>R: cactgacccacgtttctcac	71
PPIA<br>(cyclophilin A)	F: atgctggacccaacacaat<br>R: tctttcactttgccaaacacc	48
RNA18S5<br>(18S)	F: cttccacaggaggcctacac<br>R: cgcaaaatatgctggaacttt	46
MPZL2<br>(Eva1)	F: ggccgaaagagctcataaag<br>R: cagagaccttttctcttggttg	35
EBF3	F: gcacaacaattccaaacacg<br>R: gatgcacggagtggcttc	70
FBXO31	F: ccatacggaggactgctga<br>R: gtacatccacccgatgatga	77
ZIC1	F: atccacaaaaggacgcacac<br>R: gtcacagccctcaaactcg	7
CD137	F: cctgaagaccaaggagtgga<br>R: gcaaagctgattccaagagaa	54
TMEM26	F: ttgcaccatgagacccagt<br>R: tgctggtattctgtgatgttcc	64
TBX1	F: gtgccggtggacgataag<br>R: cgagtccgggtggtagtg	17
HOXC9	F: gcagcaagcacaaagagga<br>R: cgtctggtacttggtgtaggg	85
LEP<br>(leptin)	F: ttgtcaccaggatcaatgaca<br>R: gtccaaaccggtgactttct	25
ADIPOQ<br>(adiponectin)	F: ggtgagaagggtgagaaagga<br>R: tttcaccgatgtctcccttag	85
EBF2	F: ccggctctccttatggaat<br>R: aaaatgggaggatggaggat	11
SLC2A1<br>(GLUT-1)	F: ggttgtgccatactcatgacc<br>R: cagataggacatccagggtagc	67
SLC2A4<br>(GLUT-4)	F: ctgtgccatcctgatgactg<br>R: cgtagctcatggctggaact	67
ZNF516<br>(zinc finger protein 516)	F: gcctcggggttctgaagt<br>R: gtacctgtgttagaatggtctctcc	10

CEBPB	F: cgcttacctcggctacca<br>R: acgaggaggacgtggagag	74
PPARA	F: gcactggaactggatgacag<br>R: tttagaaggccaggacgatct	5
CPT1B	F: gagcagcaccccaatcac<br>R: aactccatagccatcatctgct	10
PNPLA2<br>(ATGL)	F: ctccaccaacatccacgag<br>R: ccctgcttgcacatctctc	89
PER1	F: ctcttccacagctccctca<br>R: ctttggatcggcagtggt	87
CYCS<br>(cytochrome C)	F: tgtgccagcgactaaaaaga<br>R: cctcccttttcaacggtgt	48
ATP5A1<br>(F1-ATPase alpha subunit)	F: tgctattggtcaaaagagatcca<br>R: gtagccgacaccacaatgg	61
PPARG	F: tgacaggaaagacaacagacaaat<br>R: gggtgatgtgtttgaacttgatt	7
FABP4	F: ccaccataaagagaaaacgagag<br>R: gtggaagtgacgcctttcat	31
**Murine samples**		
RN18S	F: ctcaacacgggaaacctcac<br>R: cgctccaccaactaagaacg	77
TBP<br>(TATA box binding protein)	F: gggagaatcatggaccagaa<br>R: gatgggaattccaggagtca	97
UCP1	F: ggcctctacgactcagtcca<br>R: taagccggctgagatcttgt	34
ADRB3<br>(β3-adrenoreceptor)	F: cagccagccctgttgaag<br>R: ccttcatagccatcaaacctg	13
PPARGC1A<br>(PGC-1α)	F: gaaagggccaaacagagaga<br>R: gtaaatcacacggcgctctt	29
CEBPB	F: atcgacttcagcccctacct<br>R: tagtcgtcggcgaagagg	55
PPARA	F: ccttccctgtgaactgacg<br>R: ccacagagcgctaagctgt	5
CPT1B	F: caaggaattccaggacaagact<br>R: cttcccaccagtcactcaca	64
SLC2A1<br>(GLUT-1)	F: ggaccctgcacctcattg<br>R: gccacgatgctcagatagg	20
SLC2A4<br>(GLUT-4)	F: gacggacactccatctgttg<br>R: gccacgatggagacatagc	5
ZFP516	F: ttcgccaccctctaccag<br>R: ggtgtccctgttagtgatgtcc	66
LIPE<br>(hormone sensitive lipase)	F: agcgctggaggagtgtttt<br>R: ccgctctccagttgaacc	3
PNPLA2<br>(ATGL)	F: gagcttcgcgtcaccaac<br>R: cacatctctcggaggacca	89
PER1	F: gcttcgtggacttgacacct<br>R: tgctttagatcggcagtggt	71
CYCS<br>(cytochrome C)	F: aacgttcgtggtgttgacc<br>R: ttatgcttgcctcccttttc	104
ATP5A1<br>(F1-ATPase alpha subunit)	F: gctgaggaatgttcaagcaga<br>R: ccaagttcagggacataccc	31
PPARG	F: gaaagacaacggacaaatcacc<br>R: gggggtgatatgtttgaacttg	7
FABP4	F: ggatggaaagtcgaccacaa<br>R: tggaagtcacgcctttcata	31

Experimental procedures

In vitro studies of BAT activity

Adipose tissue collection in humans

Male and female euthyroid subjects were recruited who were due to undergo elective thyroid or parathyroid surgery in the Royal Infirmary of Edinburgh. During their operation, a small amount of adipose tissue was obtained from the central compartment of the neck, superior to the clavicle and deep to the lateral thyroid lobe either adjacent to the longus colli muscle or to the oesophagus (brown adipose tissue) (Figure S1), and more superficially from the subcutaneous neck tissue (white adipose tissue). This tissue was either immediately frozen, fixed, or the stromal vascular fraction isolated and cultured for the respective experiments. Tissue collections were performed by 2 surgeons.

Adipose tissue collection in mice

Male 129/Ola mice were housed at room temperature (20˚C), 6-8 mice per cage. At 8-9 weeks of age, animals were killed and the interscapular brown adipose tissue, inguinal and epididymal white adipose tissue were immediately collected for isolation of the stromal vascular fraction.

Cell culture studies

Upon collection, adipose tissue was cut into small pieces and digested using 0.2% collagenase type 1 in Krebs-Heinseleit buffer at 37˚C for 45 minutes. Following centrifugation at 600g for 10 minutes at room temperature, the pellet was resuspended and passed through a 100μm filter then subjected to centrifugation at 200g for 5 minutes at room temperature. The pellet was re-suspended in DMEM containing 10% FBS and cultured in 6-well plates. Medium was changed every 2-3 days, and cells were passaged once 80% confluence was achieved. Confluent cells were differentiated in the above

medium with the addition of 1nM tri-iodothyronine, 20nM insulin, 500µM IBMX, 500nM dexamethasone and 125µM indomethacin for 7 days. Cells were then cultured for a further 7 days in DMEM medium containing 10% FBS, 1nM tri-iodothyronine and 20nM insulin prior to experiments. Before experiments testing the effect of varying cortisol concentrations in the medium, cells were deprived of steroids by culture in medium with charcoal stripped serum for 24 hours. Thereafter, depending on the experiments cells were cultured in serum-stripped medium with the addition of varying concentrations of either cortisol, dexamethasone, eplerenone (10µM) or RU38486 (10µM) for incubation periods of between 4 and 48 hours. In the experiments using murine adipocytes, following differentiation half of the wells containing adipocytes from the inguinal depot received 5 days incubation with the β3-agonist CL316,243 (1µM) to induce browning; this was completed prior to commencing incubation with the stripped serum. Experiments were performed after 2-3 passages.

Quantitative real time PCR measurements

Whole adipose tissue (100mg) was homogenised in Qiazol reagent using a rotor-stator homogeniser, and cells were lysed in Qiazol. RNA was extracted using the RNeasy Lipid Kit (Qiagen, Crawley, UK) and cDNA was generated using the Qiagen QuantiTect reverse transcription kit. Real time PCR was performed in triplicate using a Roche Lightcycler 480 (Roche Diagnostics Ltd, Burgess Hill, UK), using gene specific primers (Invitrogen Ltd, Paisley, UK) and fluorescent probes from the Roche Universal Probe Library unless otherwise stated. Primer sequences and probe numbers are described in Table S4. Transcript levels are presented as the ratio of the abundance of the gene of interest: mean of abundance of control genes, with PPIA and 18S used as controls in humans while TBP and 18S were used as control genes in mice.

Histology

Whole adipose tissue was fixed in 10% formalin, paraffin embedded and stained with haematoxylin and eosin prior to viewing on a Zeiss Axioskop. For immunohistochemistry, whole tissue was fixed as above while adipocytes were grown on Superfrost Plus slides and fixed using 10% formalin. Sections were incubated with rabbit anti-human UCP-1 antibody (1:8000 dilution, Sigma U6382) as the primary and goat anti-rabbit (1:200 dilution, Vectastain Elite kit, PK6101) as the secondary antibody according to the avidin-biotin complex method as follows: 1) peroxidase blocking with 3% hydrogen peroxide in methanol for 15 minutes; 2) 20% goat serum for 1 hour to reduce non-specific binding; 3) overnight incubation with primary antibody at 4˚C; 4) incubation with secondary antibody for 30 minutes at room temperature; 5) incubation with the Elite ABC reagent for 30 minutes (Vector Labs, Burlingame, CA); 6) incubation with 3,3'-diaminobenzidine for 2 minutes; 7) counterstaining with haematoxylin prior to mounting in Distyrene Plasticizer Xylene.

Respirometry

Paired pre-adipocytes from human WAT and BAT (n=6 subjects) and murine inguinal and interscapular brown depots were differentiated in XF24 V7 (human) or V28 (murine) Cell Culture Microplates (Seahorse Biosciences, Copenhagen, Denmark). White and brown adipocytes were cultured in serum-stripped medium for 24 hours, and then cultured in serum-stripped medium containing 0, 100 or 1000nM cortisol for 24 hours. Cells were then washed and placed in Seahorse XF Assay medium containing 17.5mM glucose and 1mM pyruvate. Oxygen consumption (OCR) and extracellular acidification rates (ECAR) were quantified in triplicate on a Seahorse XFe24 analyser. Measurements were performed of basal and isoprenaline (2μM) stimulated respiration, following subtraction of non-mitochondrial respiration (measured following the addition of rotenone (0.2μM) and antimycin A (2.5μM)). Basal respiration was calculated by taking the mean of 3 cycles (consisting of 2 minutes mixing, 4 minutes waiting, and 2 minutes of measurements) while stimulated

respiration was measured using the results of the 6th cycle following the addition of isoprenaline when the maximum OCR was achieved.

References

1) http://www.metoffice.gov.uk/public/weather/climate-historic/#?tab=climateHistoric
