# Supplementary material for: Glucocorticoids Acutely Increase Brown Adipose Tissue Activity in Humans, Revealing Species-Specific Differences in UCP-1 Regulation
Source: Cell Metab. 2016 Jul 12;24(1):130–41. doi: 10.1016/j.cmet.2016.06.011 (PMC4949380; doi:10.1016/j.cmet.2016.06.011)
Supplement: Document S2. Article plus Supplemental Information [file mmc2.pdf]

Article

Cell Metabolism

Glucocorticoids Acutely Increase Brown Adipose Tissue Activity in Humans, Revealing Species-Specific Differences in UCP-1 Regulation

Graphical Abstract

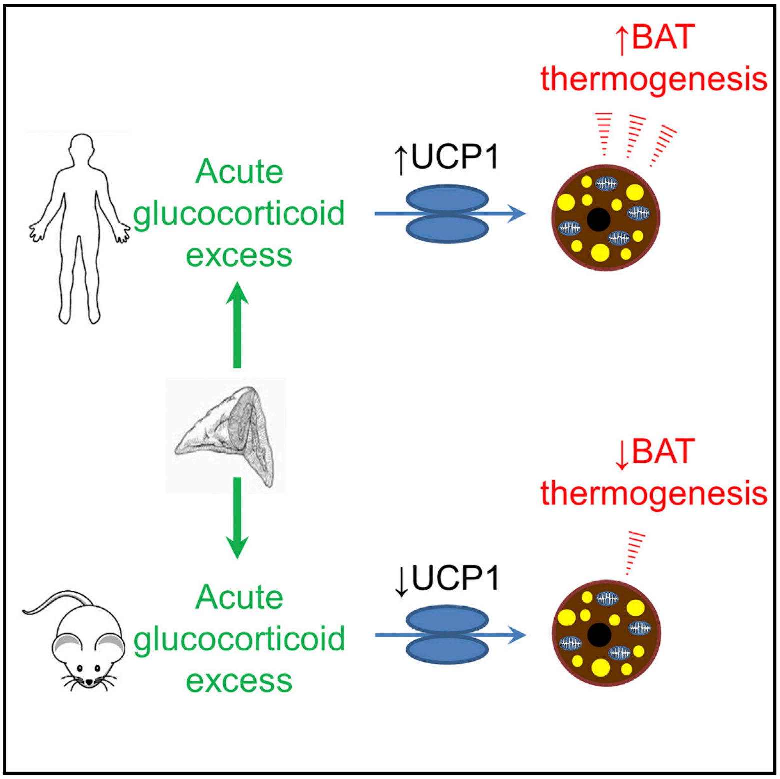

Highlights

- Glucocorticoids acutely increase but chronically suppress BAT activity in humans
- Glucocorticoids increase UCP-1 and respiration in human brown adipocytes
- Glucocorticoids decrease UCP-1 and respiration in murine brown and beige adipocytes
- Species-specific differences exist in the regulation of BAT activation

Authors

Lynne E. Ramage, Murat Akyol, Alison M. Fletcher, ..., Nicholas M. Morton, Brian R. Walker, Roland H. Stimson

Correspondence

roland.stimson@ed.ac.uk

In Brief

The regulation of brown adipose tissue (BAT) in humans is not well understood. Ramage et al. show that glucocorticoids acutely increase BAT in vivo and in vitro in humans through increasing UCP-1. However, glucocorticoids decrease UCP-1 in murine beige/ brown adipocytes, identifying species-specific differences in the regulation of BAT function.

Ramage et al., 2016, Cell Metabolism *24*, 130–141
July 12, 2016 © 2016 The Author(s). Published by Elsevier Inc.
http://dx.doi.org/10.1016/j.cmet.2016.06.011

Cell Metabolism
Article

Glucocorticoids Acutely Increase Brown Adipose Tissue Activity in Humans, Revealing Species-Specific Differences in UCP-1 Regulation

Lynne E. Ramage,[1] Murat Akyol,[2] Alison M. Fletcher,[3] John Forsythe,[2] Mark Nixon,[1] Roderick N. Carter,[1] Edwin J.R. van Beek,[3] Nicholas M. Morton,[1] Brian R. Walker,[1] and Roland H. Stimson[1],*
[1]British Heart Foundation/University Centre for Cardiovascular Science, University of Edinburgh, Edinburgh EH16 4TJ, Scotland, UK
[2]Department of Surgery, Royal Infirmary of Edinburgh, Edinburgh EH16 4SA, Scotland, UK
[3]Clinical Research Imaging Centre, University of Edinburgh, Edinburgh EH16 4TJ, Scotland, UK
*Correspondence: roland.stimson@ed.ac.uk
http://dx.doi.org/10.1016/j.cmet.2016.06.011

SUMMARY

The discovery of brown adipose tissue (BAT) in adult humans presents a new therapeutic target for metabolic disease; however, little is known about the regulation of human BAT. Chronic glucocorticoid excess causes obesity in humans, and glucocorticoids suppress BAT activation in rodents. We tested whether glucocorticoids regulate BAT activity in humans. In vivo, the glucocorticoid prednisolone acutely increased ^{18}fluorodeoxyglucose uptake by BAT (measured using PET/CT) in lean healthy men during mild cold exposure (16°C–17°C). In addition, prednisolone increased supraclavicular skin temperature (measured using infrared thermography) and energy expenditure during cold, but not warm, exposure in lean subjects. In vitro, glucocorticoids increased isoprenaline-stimulated respiration and UCP-1 in human primary brown adipocytes, but substantially decreased isoprenaline-stimulated respiration and UCP-1 in primary murine brown and beige adipocytes. The highly species-specific regulation of BAT function by glucocorticoids may have important implications for the translation of novel treatments to activate BAT to improve metabolic health.

INTRODUCTION

The recent discovery of brown adipose tissue (BAT) in adult humans (Virtanen et al., 2009; van Marken Lichtenbelt et al., 2009; Saito et al., 2009) has revealed the exciting possibility of activating BAT and/or "browning" white adipose tissue (WAT) to enhance energy expenditure and treat obesity, diabetes, and even dyslipidemia (Sidossis and Kajimura, 2015). Numerous factors have been identified that induce browning in rodents, such as FGF21, BMP8b, and natriuretic peptides (Sidossis and Kajimura, 2015). However, very little is known about the function and regulation of human BAT. Findings to date suggest similarities between rodent and human BAT, for example, both are activated by cold exposure (Saito et al., 2009) and sympathetic activity (Cypess et al., 2015) and contribute to cold-induced thermogenesis (Ouellet et al., 2012). Recent work has focused on whether human BAT is more closely related to rodent"classic" brown or inducible "beige" fat (Jespersen et al., 2013; Cypess et al., 2013; Shinoda et al., 2015), with less emphasis on determining if there are substantial differences between species in the regulation of BAT function. Analysis of hormonal regulation of BAT in humans has been largely overlooked to date, in part because of the difficulty in obtaining samples for in vitro studies or quantifying in vivo function of human BAT.

Glucocorticoids are powerful regulators of energy metabolism, and chronic glucocorticoid excess causes obesity, type 2 diabetes mellitus, and dyslipidemia (Macfarlane et al., 2008). In rodents, it has long been known that glucocorticoids suppress UCP-1 and thermogenesis by BAT, while adrenalectomy and the glucocorticoid receptor antagonist RU38486 both increase BAT thermogenesis and UCP-1 levels (Hardwick et al., 1989; Strack et al., 1995; van den Beukel et al., 2014). In addition, glucocorticoids suppress browning of WAT in mice (Kong et al., 2015). While circulating glucocorticoid levels are regulated by the hypothalamic-pituitary-adrenal (HPA) axis, tissue glucocorticoid levels in important metabolic tissues such as liver and adipose tissue are further amplified by the enzyme 11β-hydroxysteroid dehydrogenase type 1 (11β-HSD1) (Stimson et al., 2009). In murine brown adipocytes, overexpression of 11β-HSD1 decreases BAT function, while pharmacological 11β-HSD1 inhibition or knockdown enhances BAT activation (Liu et al., 2013), highlighting that local glucocorticoid excess inhibits BAT activity in rodents. We tested the hypothesis that glucocorticoids suppress BAT activation in humans as in rodents using in vivo and in vitro approaches.

RESULTS

Glucocorticoids Increase Cold-Induced ^{18}FDG Uptake by Human BAT In Vivo

Six healthy men (subject characteristics in Table 1) were recruited to a randomized double-blind, placebo-controlled crossover study (Figure 1A) to determine the effect of glucocorticoids on BAT activity. The synthetic glucocorticoid prednisolone (10 mg every 12 hr) or placebo was administered for three doses prior to each study visit, and positron emission tomography/computed tomography (PET/CT) was used to measure ^{18}fluorodeoxyglucose (^{18}FDG)

 © 2016 The Author(s). Published by Elsevier Inc.
This is an open access article under the CC BY license (http://creativecommons.org/licenses/by/4.0/).

Table 1. Anthropometric and Biochemical Measurements in In Vivo Studies

	PET/CT Study		Thermal Imaging Study	
	Prednisolone	Placebo	Prednisolone	Placebo
No. of participants (male/female)	6/0		5/4	
Age (years)	22.1 ± 1.2	22.1 ± 1.2	22.7 ± 1.3	22.7 ± 1.3
Weight (kg)	68.7 ± 3.1	68.6 ± 3.1	65.3 ± 2.9	65.3 ± 2.8
BMI (kg/m^2)	22.0 ± 0.9	22.0 ± 0.9	21.8 ± 0.6	21.8 ± 0.6
Fat mass (kg)	8.6 ± 0.9	8.4 ± 0.9	12.5 ± 1.2	12.4 ± 1.1
Systolic BP (mmHg)	128 ± 5	123 ± 5	116 ± 3	114 ± 2
Diastolic BP (mmHg)	70 ± 2	69 ± 1	66 ± 2	66 ± 3
Warm environmental temperature (°C)	23.8 ± 0.2	23.5 ± 0.1	23.7 ± 0.3	24.0 ± 0.3
Cold environmental temperature (°C)	17.5 ± 0.2$^{###}$	17.2 ± 0.4$^{###}$	16.5 ± 0.2$^{###}$	16.2 ± 0.1$^{###}$
Fasting Biochemistry				
Glucose (mmol/L)	6.0 ± 0.3*	5.3 ± 0.3	5.0 ± 0.1*	4.4 ± 0.1
Insulin (pmol/L)	66 ± 11*	40 ± 7	56 ± 7**	28 ± 5
Total cholesterol (mmol/L)	3.8 ± 0.2	3.8 ± 0.2	4.1 ± 0.2	4.1 ± 0.2
Triglycerides (mmol/L)	0.6 ± 0.0	0.7 ± 0.1	0.8 ± 0.1	0.8 ± 0.1
NEFAs (warm room) (μmol/L)	558 ± 82*	362 ± 50	351 ± 53	244 ± 53
NEFAs (cold room) (μmol/L)	515 ± 66	391 ± 34	453 ± 54*	277 ± 71
ACTH (warm room) (ng/L)	ND**	47 ± 6	ND**	49 ± 8
ACTH (cold room) (ng/L)	ND**	22 ± 6$^{##}$	ND**	26 ± 3$^{#}$
Noradrenaline (warm room) (ng/L)	305 ± 74	304 ± 82	492 ± 66	612 ± 86
Noradrenaline (cold room) (ng/L)	451 ± 120	575 ± 141	564 ± 102	611 ± 123

Data are mean ± SEM. ND, not detected. *$p < 0.05$, **$p < 0.01$ versus placebo. $^{#}p < 0.05$, $^{##}p < 0.01$, $^{###}p < 0.001$ versus measurements in warm room.

uptake by BAT during 2 hr of mild cold exposure (16°C–17°C) (van Marken Lichtenbelt et al., 2009). Study visits took place between March and September in Edinburgh. Average local environmental temperatures over that period are detailed in Table S1, available online. No subject had any symptoms or signs of shivering during cold exposure. Prednisolone increased fasting plasma glucose, insulin, and non-esterified fatty acids (NEFAs) and decreased adrenocorticotrophic hormone (ACTH) and cortisol concentrations compared with placebo, but did not alter noradrenaline concentrations (Table 1). All six subjects had detectable ^{18}FDG uptake by BAT. Prednisolone increased cold-induced ^{18}FDG uptake by BAT (median total standardized uptake value 254 [interquartile range, IQR 159, 556] versus 164 [98, 209] cm^3 × g/mL, $p = 0.02$) (Figures 2A and 2B). In addition, prednisolone tended to increase the volume of active BAT (71 ± 25 versus 50 ± 25 cm^3, $p = 0.07$).

During the placebo phase, plasma cortisol concentrations fell following normal diurnal variation while subjects were in a warm environment, but did not fall further during cold exposure (Figure 2C). Despite increasing glucose uptake by BAT, prednisolone substantially decreased whole-body 6,6-[^{2}H]$_2$-glucose (D2-glucose) uptake during both warm and cold conditions, while whole-body D2-glucose uptake was unchanged by cooling on either placebo or prednisolone phases (Figure 2D).

Glucocorticoids Increase Energy Expenditure and Supraclavicular Temperature during Cold Exposure

The PET study showed, surprisingly, that glucocorticoids increased cold-induced ^{18}FDG uptake by BAT, suggesting that glucocorticoids may increase BAT thermogenesis in humans, unlike in rodents. To test the effect of prednisolone on cold-induced thermogenesis (using indirect calorimetry) and heat production by BAT (using thermal imaging; Lee et al., 2011), we performed a similar placebo-controlled, randomized crossover study (Figure 1B) using the same dosing structure. Subject characteristics are detailed in Table 1. Study visits took place between January and May in the same research facility. Average local environmental temperatures over that period are detailed in Table S1. No subject had any symptoms or signs of shivering during cold exposure. As in the PET study, prednisolone increased fasting plasma glucose and insulin and suppressed ACTH (Table 1) and cortisol (10 ± 3 versus 417 ± 22 nmol/L) concentrations. Prednisolone did not alter resting energy expenditure in a warm environment, but increased energy expenditure during cold exposure (Figure 3D). Prednisolone substantially increased cold-induced thermogenesis compared with placebo (286 ± 68 versus 125 ± 37 kcal/24 hr, $p = 0.01$).

Prednisolone did not alter skin temperature in the supraclavicular region (BAT) or anterior chest (control) regions in warm conditions (Figures 3B and 3E). In addition, supraclavicular skin temperature did not significantly change following hand immersion in 15°C water for 10 min during either prednisolone or placebo phases (+0.0°C ± 0.1°C and +0.1°C ± 0.1°C, respectively). However, during cold exposure prednisolone increased supraclavicular skin temperature compared with placebo (Figures 3A–3C) but did not alter anterior chest (Figures 3E and 3F) or peripheral (hand) skin temperature (26.1°C ± 0.7°C versus 25.5°C ± 0.7°C).

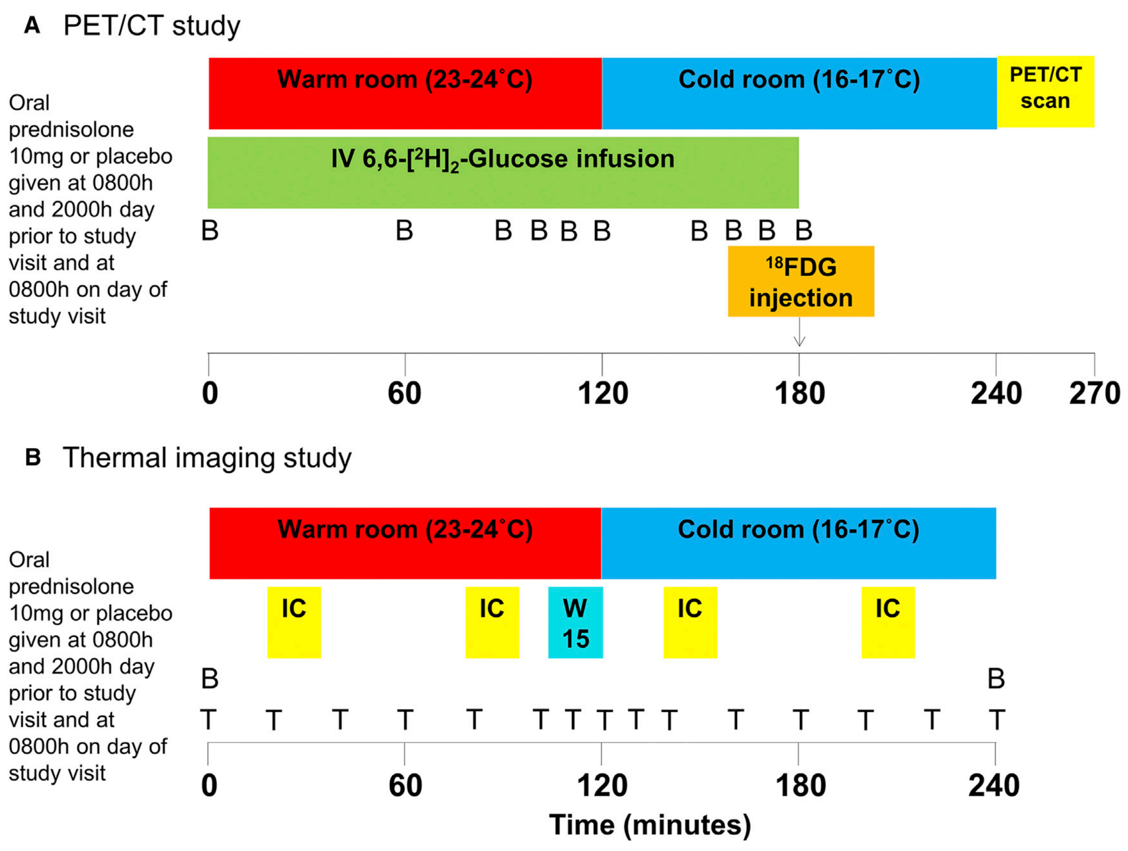

Figure 1. In Vivo Study Visit Protocols

Prednisolone regulation of human BAT activity was measured in vivo in two separate studies using (A) PET/CT and (B) thermal imaging. In both studies, participants spent 2 hr in a warm room, then 2 hr in a cold room to activate BAT. In the PET/CT study, 1 hr into cold exposure, subjects were injected with 75 MBq of 18-fluoro-2-deoxyglucose (^{18}FDG), and a PET/CT scan was performed 1 hr later. Blood (B) samples were taken at the intervals shown. In the thermal imaging study, at the end of the second hour in the warm room, subjects placed both hands in water cooled to 15°C (W15) as a mild stimulant to activate BAT. Thermal imaging was performed at the intervals shown (T). Whole-body energy expenditure was assessed each hour using indirect calorimetry (IC).

Distinct Regulation of Human BAT Activity by Glucocorticoids In Vitro

BAT Biopsy and Culture from Human Deep Supraclavicular Adipose Depots

A significant challenge to investigating BAT regulation in humans has been the shortage of reliable sources of tissue from which to culture brown adipocytes. To investigate the mechanism whereby glucocorticoids enhance human BAT activation, we collected adipose tissue samples from euthyroid patients undergoing elective thyroid or parathyroid surgery. Characteristics of the participants are detailed in Table S2.

Initial work focused on obtaining human BAT without prior PET/CT scanning. Paired samples were obtained from the superficial WAT and adipose tissue depots posterior to the thyroid (Figure S1), the typical location of human BAT (Cypess et al., 2013). The deeper tissue had substantially higher transcript levels of the typical BAT genes UCP-1 (~1,000-fold), β3-adrenoreceptor (β3-AR), PGC-1α, and deiodinase type 2 (DIO2) (Figure 4A). Furthermore, although both depots consisted predominantly of white adipocytes, only tissue from the deeper depot also contained clusters of cells with the typical appearance of brown adipocytes, and immunohistochemistry confirmed only these cells contained UCP-1 (Figures 4B and 4C), proving that BAT could be successfully obtained from humans without the need for prior PET/CT scanning. mRNA levels of the glucocorticoid receptor-α (GRα), mineralocorticoid receptor (MR), cortisol-regenerating enzyme 11β-hydroxysteroid dehydrogenase type 1 (11β-HSD1), and cortisol-inactivating enzyme 11β-HSD2 were similarly expressed in BAT and WAT, indicating that human BAT is likely to be a glucocorticoid-responsive tissue (Figure 4A). To determine whether obesity was associated with dysregulated glucocorticoid signaling in BAT, correlations between body mass index (BMI) and GRα, MR, 11β-HSD1, and 11β-HSD2 mRNA levels were tested in an expanded dataset ($n = 21$). 11β-HSD2 mRNA levels positively correlated with BMI in BAT ($r^2 = 0.31$, $p < 0.05$), but not WAT ($r^2 = 0.00$), while no other significant associations were found.

In a separate patient cohort, we cultured the stromal vascular fraction from the superficial and deeper supraclavicular adipose tissue. Differentiated pre-adipocytes from the deeper depot (hereafter termed brown adipocytes) retained high expression of the typical BAT genes UCP-1, β3-AR, PRDM16, and DIO2 compared with differentiated pre-adipocytes from the superficial depot (termed white adipocytes) (Figure 4D). Furthermore, immunocytochemistry revealed that only the cultured brown adipocytes contained UCP-1 (Figure 4E). The brown adipocytes

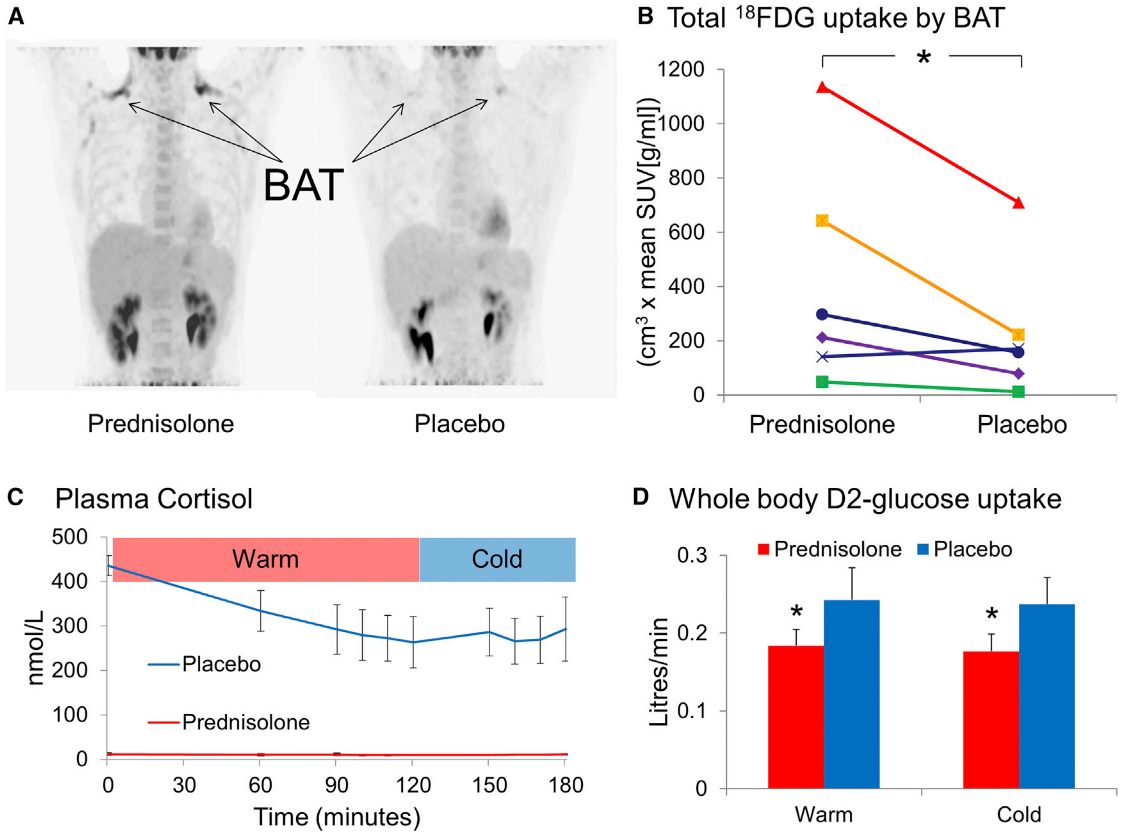

Figure 2. Glucocorticoids Increase Cold-Induced ^{18}FDG Uptake by BAT

(A) Paired PET/CT scans from a subject demonstrating increased ^{18}FDG uptake in the supraclavicular BAT (arrows) during the prednisolone phase (left panel).
(B) Paired total ^{18}FDG uptake by BAT for all six subjects; prednisolone increased uptake by BAT compared with placebo. Data were analyzed using the Wilcoxon signed-rank test.
(C) Plasma cortisol concentrations, shown as mean ± SEM (n = 6), fell following normal diurnal variation in the warm room ($p < 0.05$ using repeated-measures ANOVA) but did not continue to fall during cooling (t + 120 onward) during the placebo phase (blue line), and were appropriately suppressed by prednisolone (red line).
(D) Prednisolone (red columns) decreased whole-body D2-glucose uptake compared with placebo (blue columns); whole-body D2-glucose uptake was not altered by cold exposure. Data were analyzed using the paired t test. *$p < 0.05$, prednisolone versus placebo.

had substantially higher expression of 11β-HSD1 and lower expression of 11β-HSD2, while GRα and MR transcript levels were similar between cell types (Figure 4D).

Recent conflicting reports suggest human BAT is more akin either to rodent "beige" or to interscapular "classic" BAT (Shinoda et al., 2015; Cypess et al., 2013; Jespersen et al., 2013). We measured transcripts of recently published classic brown and beige genes, and determined that human brown adipocytes have high expression of the beige genes TMEM26 and Tbx1, but also high expression of the typical classic brown genes ZIC1 and Ebf3 (Figure S2). In addition, mRNA levels of the recently described brown and beige adipocyte marker Ebf2 (Wang et al., 2014) were higher in the brown than white adipocytes (0.89 ± 0.17 versus 0.62 ± 0.13 a.u., $p < 0.04$). This confirms previous work suggesting the molecular signature partially overlaps (Cypess et al., 2013; Jespersen et al., 2013), although this may vary between different depots of human BAT.

Glucocorticoid Effects on Cultured Human White and Brown Adipocytes

To determine whether glucocorticoids enhance oxygen consumption by brown adipocytes in vitro, human brown and white adipocytes were cultured for 24 hr in 0, 100, or 1,000 nM cortisol. Oxygen consumption was measured on a Seahorse XFe24 analyzer. Cortisol at 100 nM increased basal and isoprenaline-stimulated respiration by brown adipocytes compared with 0 and 1000 nM cortisol (Figure 5A). Cortisol did not alter either basal or stimulated respiration in the white adipocytes (Figure S3A). Glucocorticoids did not alter the extracellular acidification rate in either brown or white adipocytes (data not shown). These results, in addition to confirming the in vivo findings, revealed that glucocorticoids enhance brown adipocyte activity directly.

To determine whether glucocorticoids enhance BAT activity by regulating UCP-1, human primary brown adipocytes were cultured for 24 hr in 0, 100, or 1,000 nM cortisol, as above. As before, UCP-1 levels were substantially increased in the brown adipocytes compared with the white adipocytes (>25-fold; data not shown). The 100 nM cortisol concentration stimulated UCP-1 mRNA levels compared with both 0 and 1,000 nM cortisol (Figure 5C). The effect of glucocorticoids on other mechanisms that could contribute to BAT activation was also examined. In parallel with the increased respiration and UCP-1 mRNA levels,

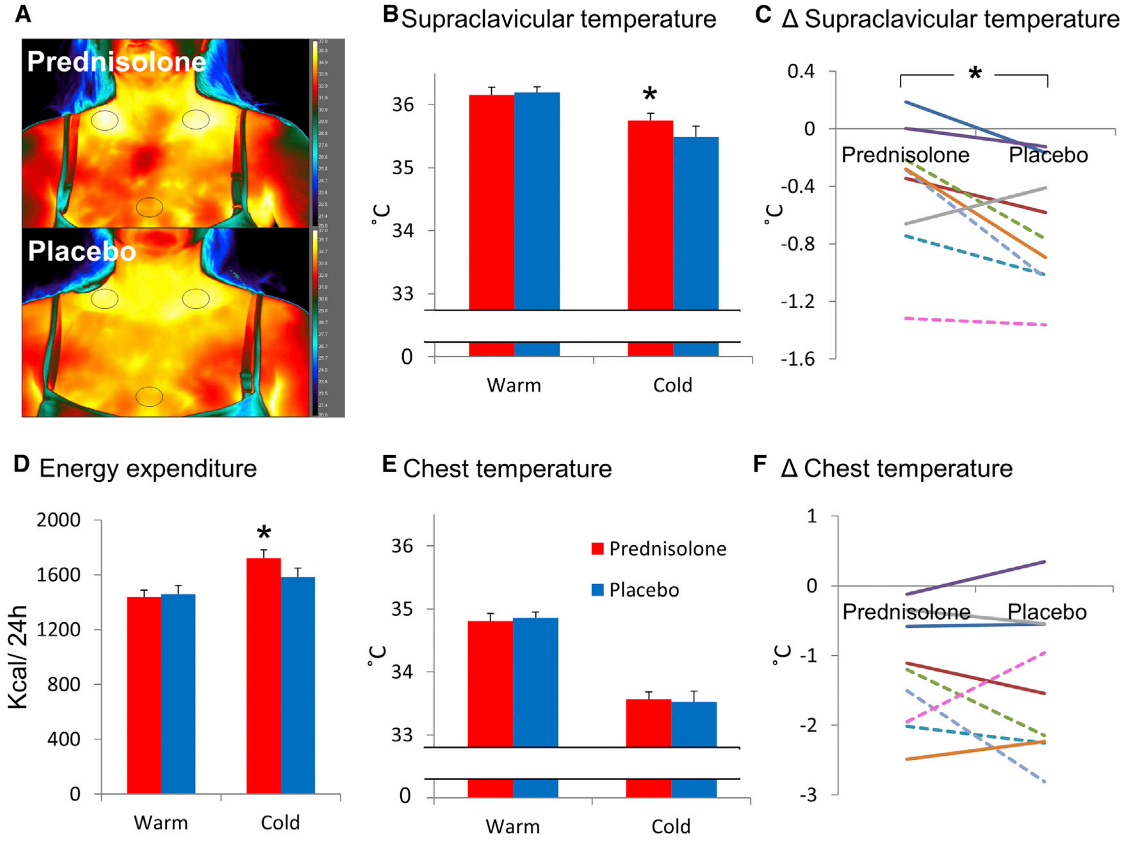

Figure 3. Glucocorticoids Increase Supraclavicular Skin Temperature and Cold-Induced Thermogenesis

(A) Paired thermal images obtained from a subject on prednisolone (top panel) and placebo (lower panel) phases during cold exposure, showing increased skin temperature in the supraclavicular regions of interest (black ovals) on the prednisolone phase.

(B) Prednisolone (red columns) increased supraclavicular skin temperature compared with placebo (blue columns) during cold exposure only; shown as mean ± SEM (n = 9). Data are analyzed by paired t tests.

(C) The reduction in mean supraclavicular skin temperature in male (solid lines) and female (dotted lines) subjects, expressed as the absolute difference between warm and cold environment, induced by cold exposure, was attenuated by prednisolone treatment.

(D) Prednisolone increased energy expenditure during cold, but not warm, exposure.

(E and F) Prednisolone did not alter (E) chest temperature during warm or cold exposure and did not affect (F) the reduction in mean chest temperature induced by cold compared to warm exposure. *$p < 0.05$ versus placebo.

100 nM cortisol increased GLUT-4 mRNA levels in brown adipocytes (Figure 5C) but did not alter β3-AR, C/EBPβ, PPARα, CPT-1b, GLUT-1, or ZFP516 (the recently described upstream regulator of UCP-1; Dempersmier et al., 2015). High-dose cortisol (1,000 nM) increased PGC-1α mRNA levels in both brown and white adipocytes. In white adipocytes, cortisol did not alter mRNA levels of the other above genes (Figure S3B). Glucocorticoids did not alter mRNA levels of the mitochondrial markers cytochrome *c* and F1-ATPase or markers of adipocyte differentiation in brown adipocytes, indicating that glucocorticoids were not simply enhancing mitochondrial number and activity (Figures S3C and S3D). In addition, markers of adipocyte differentiation were similar between white and brown adipocytes (PPARγ 0.80 ± 0.14 [WAT] versus 0.96 ± 0.11 [BAT] a.u., FABP4 1.14 ± 0.32 [WAT] versus 0.87 ± 0.13 [BAT] a.u.).

To determine whether glucocorticoids increase UCP-1 via the glucocorticoid receptor (GR), human primary brown adipocytes were initially incubated with the GR antagonist RU38486. However, RU38486 substantially increased UCP-1 mRNA levels in glucocorticoid-free medium (1.6 ± 0.8 versus 0.4 ± 0.2 a.u., $p < 0.05$), as shown previously in murine brown adipocytes (Rodríguez and Palou, 2004), meaning RU38486 could not be used for this purpose. Therefore, human primary brown adipocytes were incubated for 24 hr with 0, 100, or 1,000 nM cortisol or equivalent doses of dexamethasone (a selective GR agonist ~27-fold more potent than cortisol) in the presence or absence of the mineralocorticoid receptor antagonist eplerenone. Both 100 nM cortisol and 3.75 nM dexamethasone increased UCP-1 mRNA levels by ~40% (Figure 5E), which was not reversed by eplerenone. These results confirmed that high physiological, but not supraphysiological, glucocorticoid concentrations increase UCP-1 and determined that this effect is most likely mediated through GR.

Contrasting Effects of Glucocorticoids in Human and Murine Brown and Beige Adipocytes

These findings that glucocorticoids enhance BAT activity in humans both in vivo and in vitro, most likely by increasing UCP-1, are at odds with consistent previous reports that glucocorticoids

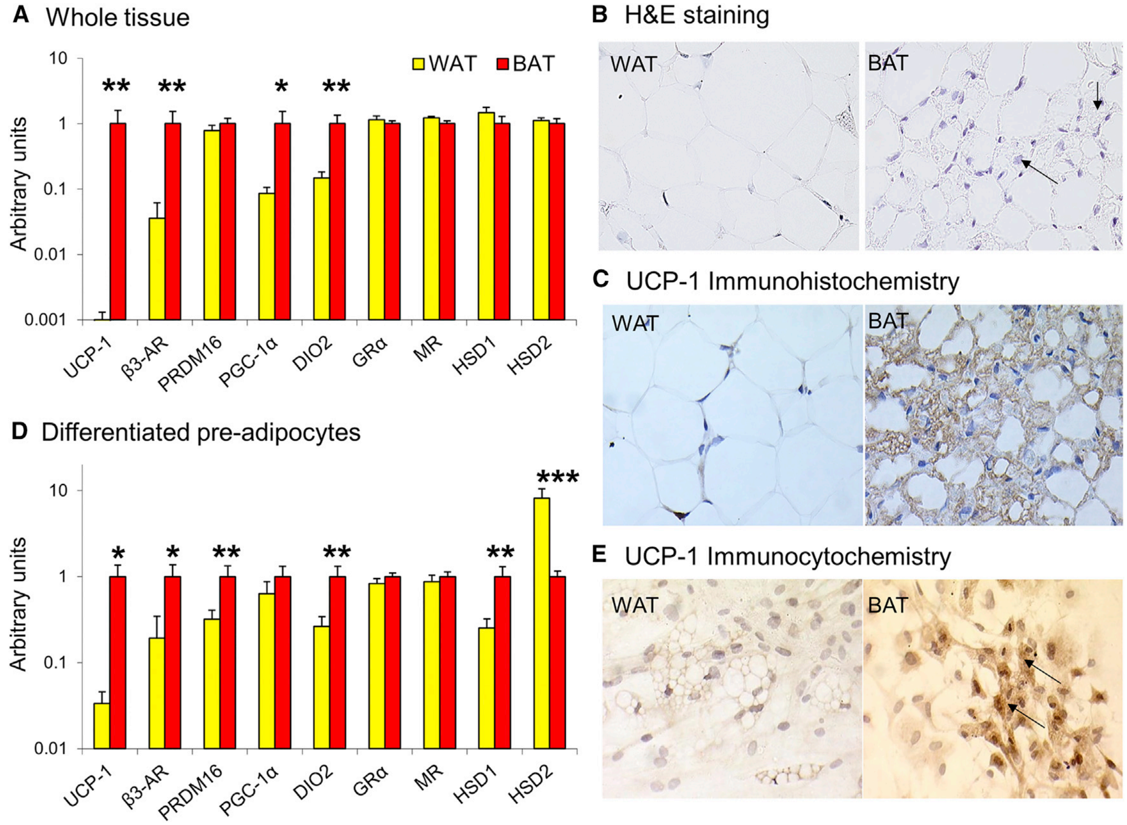

Figure 4. Human BAT Biopsy and Cell Culture

(A) mRNA transcripts are shown as mean ± SEM for n = 9 for whole adipose tissue collected from the superficial (WAT, yellow columns) and deeper (BAT, red columns) neck depots.

(B) Images obtained using 40× magnification show that the superficial depot only contained adipocytes with the typical appearance of white adipocytes, while the deeper depot also contained smaller cells with multilocular lipid droplets, more rounded nuclei, and basophilic cytoplasm (arrows), which are the typical features of brown adipocytes.

(C) Immunohistochemistry (40× magnification) showing that only the brown adipocyte-like cells contained UCP-1 in their cytoplasm (brown staining).

(D) mRNA transcripts are shown as mean ± SEM for n = 11 for differentiated pre-adipocytes cultured from the superficial (WAT) and the deeper (BAT) depots, showing that the BAT cells maintained a similar gene expression pattern to the whole tissue.

(E) Immunohistochemistry (40× magnification) showing that only the cultured cells from the BAT depot contained UCP-1 (brown staining, black arrows) in their cytoplasm. Data were analyzed using paired t tests. $^{*}p < 0.05$, $^{**}p < 0.01$, $^{***}p < 0.001$ versus WAT.

suppress BAT activation in rodents. Most of these studies have used supraphysiological concentrations of glucocorticoids for longer duration than in the human experiments above, and have usually examined only interscapular classic BAT, which may not be analogous with human BAT. To compare glucocorticoid regulation of BAT in humans and mice, we obtained the epididymal and inguinal WAT and interscapular BAT from the 129 mouse strain (which readily develop browning of their inguinal adipose depot; Wu et al., 2012) and cultured and differentiated the stromal vascular fractions as in the human cells. To induce browning of the inguinal adipocytes, following differentiation half of these cells were treated with the β3-adrenoreceptor agonist CL316,243 for 5 days (Figure S4A). Differentiated adipocytes were then incubated with 0, 100, or 1,000 nM cortisol for 24 hr, as above. In marked contrast to the human brown adipocytes, 100 and 1,000 nM cortisol suppressed UCP-1 mRNA levels by ~80%–85% in inguinal (Figure 5D), CL316,243-treated inguinal, and interscapular brown adipocytes (Figure S4A–S4C). UCP-1 levels were extremely low in the epididymal WAT and were unchanged by cortisol. Cortisol increased PGC-1α and GLUT-4 mRNA levels in the inguinal adipocytes (Figure 5D) as in human brown adipocytes and decreased β3-AR, C/EBPβ, and GLUT-1 mRNA levels in the brown adipocytes (Figure S4C). Cortisol similarly induced classic glucocorticoid-regulated genes such as HSL, ATGL, and PER1 in both human and murine white and brown adipocytes (Figures S3C, S3D, and S4D–S4F), highlighting that cortisol was activating GR in both species. To determine whether glucocorticoids also reduced respiration in murine cells, basal and isoprenaline-stimulated oxygen consumption was measured in the murine beige and brown adipocytes incubated with cortisol for 24 hr as above. In contrast to the human brown adipocytes, 100 and 1,000 nM cortisol strongly suppressed isoprenaline-stimulated respiration in both inguinal (Figure 5B), CL316,243-treated inguinal, and brown adipocytes (Figures S5A and S5B).These results strongly suggested that glucocorticoid regulation of UCP-1 was species specific; however, to determine whether lower cortisol concentrations with shorter incubation periods could stimulate UCP-1, we incubated

murine inguinal (unstimulated and CL316,243 treated) and brown adipocytes with either 0, 25, 50, 100, or 1,000 nM cortisol for both 4 and 8 hr. None of the cortisol concentrations increased UCP-1 mRNA levels in either the brown (Figure 5F) or beige adipocytes (Figures S4G and S4H) following 4 hr incubation, and by 8 hr even the lowest cortisol concentration suppressed UCP-1 in the brown adipocytes (Figure 5F).

Chronic Glucocorticoid Excess Suppresses In Vivo BAT Activity in Humans

Chronic glucocorticoid excess causes obesity, so to determine whether this acute stimulatory effect on BAT activity was maintained over the longer term, primary human brown adipocytes were cultured in 0, 100, and 1,000 nM cortisol for 48 hr. The 100 nM cortisol concentration failed to increase UCP-1 following 48 hr incubation, while 1,000 nM cortisol substantially suppressed UCP-1 (Figure 6A). This suggested that the acute stimulatory effect on BAT activation was unlikely to be maintained more chronically. To test this further, we performed a retrospective analysis of all patients who had undergone PET/CT scanning in the Royal Infirmary of Edinburgh over the past 2 years. We identified 129 patients who had been taking oral glucocorticoids for at least 2 weeks at the time of their scan and 120 age-, sex-, BMI-, glucose-, and disease-matched controls with no history of any glucocorticoid use over the preceding year (Table S3). Ten of the 249 patients (4.0%) had detectable ^{18}FDG uptake by BAT; these “BAT-positive” patients were younger (54.4 ± 6.2 versus 64.4 ± 1.0 years, $p < 0.05$) and had lower BMI (20.6 ± 0.7 versus 25.3 ± 0.3 kg/m^2, $p < 0.01$) than the “BAT-negative” patients, in agreement with previous data (Saito et al., 2009; Cypess et al., 2009). Significantly fewer glucocorticoid-treated patients had detectable BAT compared to controls (Figure 6B). The controls had greater BAT volume and total ^{18}FDG uptake by BAT than glucocorticoid-treated patients (Figures 6C and 6D). In contrast to acute glucocorticoid treatment, this shows that chronic glucocorticoid treatment suppresses BAT activation in humans.

DISCUSSION

This work shows that glucocorticoids acutely increase BAT activity in humans. In vivo, the glucocorticoid prednisolone increased ^{18}FDG uptake by BAT, supraclavicular temperature, and cold-induced thermogenesis during cold exposure in healthy volunteers. Furthermore, at physiological concentrations the endogenous glucocorticoid cortisol increased basal and isoprenaline-stimulated respiration in human brown, but not white, adipocytes and increased UCP-1 mRNA levels, an effect most likely mediated through the glucocorticoid receptor. This was in marked contrast to rodent brown and beige adipocytes, where cortisol substantially suppressed isoprenaline-stimulated respiration and UCP-1. Lower dose and shorter cortisol incubations also failed to induce UCP-1 in the murine adipocytes, confirming the species-specific regulation of UCP-1 by glucocorticoids. These findings highlight the challenges of translating rodent findings into humans, particularly with regard to the new factors discovered to activate BAT or induce “browning” in rodents, which are being proposed as possible new therapeutic targets for human obesity (Sidossis and Kajimura, 2015). In addition, these findings highlight the importance of studying human BAT both in vivo and in vitro and suggest, at least in relation to regulation by glucocorticoids, that the difference between species is greater than any differences between beige and classic BAT. These findings also highlight the complex tissue-specific actions of glucocorticoids, decreasing whole-body glucose uptake (by inducing insulin resistance in tissues such as muscle) but increasing glucose uptake by BAT. Whole-body glucose uptake was not increased during cold exposure, signifying that the increased glucose uptake by BAT was insufficient to impact on whole-body glucose utilization at least during the first hour of cold exposure.

Only one previous study has examined regulation of human BAT by glucocorticoids (Barclay et al., 2015), demonstrating only in vitro that the potent glucocorticoid dexamethasone suppressed isoprenaline-stimulated UCP-1 levels and oxygen consumption. However, the glucocorticoid concentrations used were 30- and 300-fold higher in potency than those in the current in vitro experiments. Since we observed that 100 nM, but not 1,000 nM, cortisol stimulated BAT activity, it is possible that even higher glucocorticoid concentrations would have suppressed BAT activity in vitro. Endogenous cortisol levels in adipose tissue are approximately 25 nM (Hughes et al., 2010), so levels of 100 nM in adipose tissue are likely to be physiologically relevant during stress. In addition, 20 mg of prednisolone per day (which is ~4-fold more potent than cortisol), as used in our in vivo studies, would increase glucocorticoid exposure ~4- to 5-fold higher than in normal health (Kraan et al., 1998), which is equivalent to cortisol production during severe stress (Boonen et al., 2013). Therefore, high physiological glucocorticoid levels for 24 hr both in vivo and in vitro induce BAT activation.

It appears that glucocorticoid-induced thermogenesis represents a physiological response in humans. While cortisol concentrations during placebo administration did not rise during cold exposure in this study or others (Ouellet et al., 2012), cortisol did not continue to fall, as would be expected from normal diurnal variation. Severe cold exposure sufficient to induce shivering does increase cortisol secretion (Wilson et al., 1970). We can speculate that during severe cold exposure (or, indeed, in combination with enhanced adrenaline secretion in the fight/flight response), the increased cortisol secretion enhances BAT thermogenesis to protect core body temperature. Furthermore, recent work has determined that there is a circadian rhythm to BAT thermogenesis in humans with elevated BAT function coinciding with peak cortisol secretion early in the morning, so it is plausible that cortisol may in part drive this circadian rhythm (Lee et al., 2016).

The value of infrared thermography to measure BAT activity is debated due to potential confounders such as heat production from other tissues and changes in overlying skin blood flow; however, our results using this technique were similar to those obtained using PET/CT, suggesting that infrared thermography can be used to detect cold-induced BAT activation. Importantly, we minimized potential confounders by ensuring that subjects wore identical clothing and that room temperatures were similar between visits. Furthermore, it is unclear whether infrared thermography data should be presented as absolute supraclavicular skin temperatures or as the temperature gradient between the supraclavicular skin and anterior chest (Jang et al., 2014). However, our results were similar when calculated using the

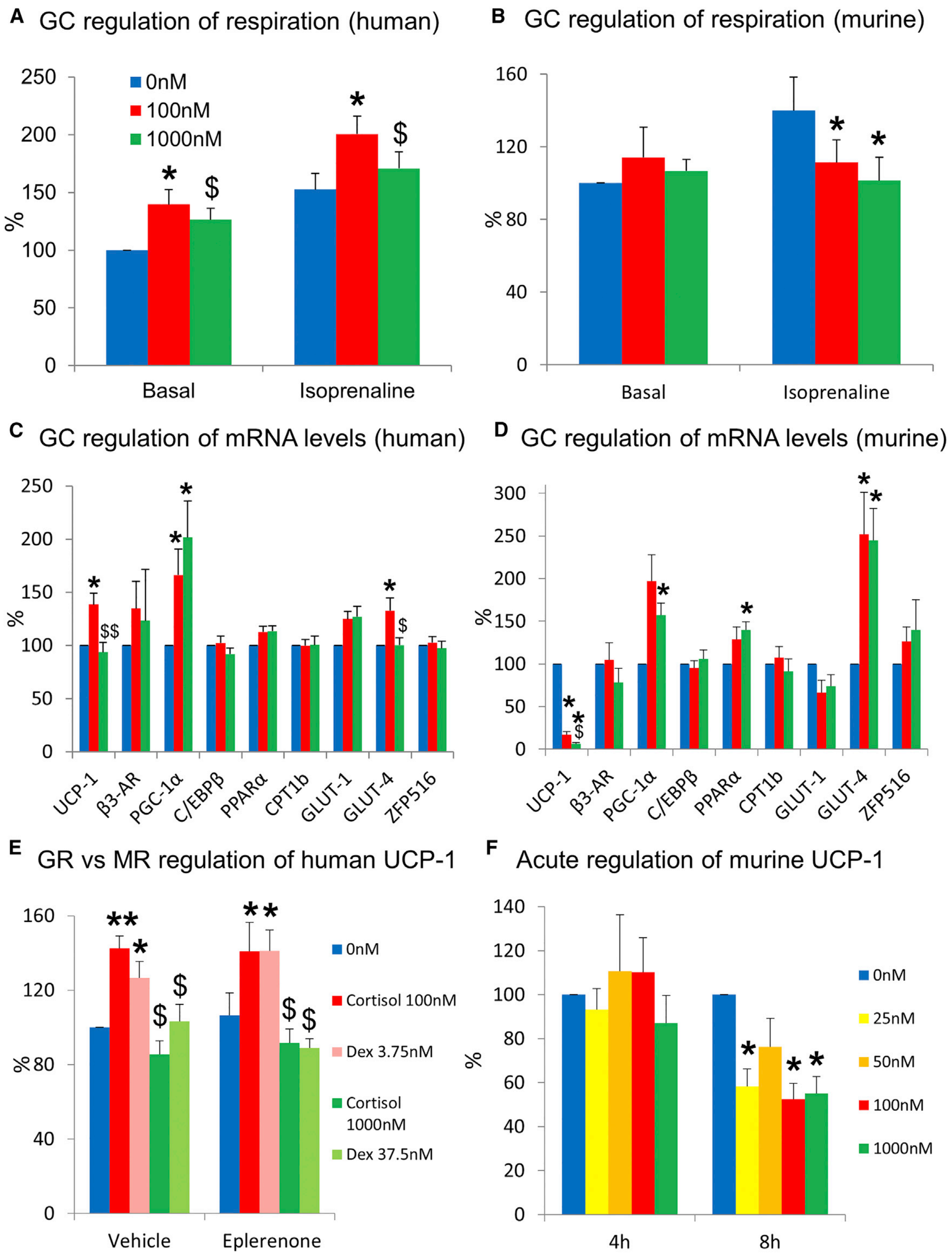

Figure 5. Glucocorticoid Regulation of Human and Murine Brown Adipocytes In Vitro

(A and B) Data are mean ± SEM for paired (A) human brown adipocytes (n = 6) or (B) murine inguinal adipocytes (n = 7) cultured for 24 hr in either 0 (blue columns), 100 (red columns), or 1,000 nM (green columns) cortisol. Cortisol (100 nM) increased basal and isoprenaline-stimulated oxygen consumption compared with 0 and 1,000 nM in the human brown adipocytes, but 100 and 1,000 nM cortisol decreased isoprenaline-stimulated oxygen consumption in the murine adipocytes, with basal 0 nM normalized to 100%.

(C and D) mRNA levels (with 0 nM normalized to 100%) from paired (C) human brown adipocytes (n = 8) or (D) inguinal beige adipocytes (n = 6) following 24 hr incubation with cortisol at 0, 100, and 1,000 nM. Cortisol (100 nM) increased UCP-1 levels in human brown adipocytes but decreased UCP-1 in murine adipocytes.

(legend continued on next page)

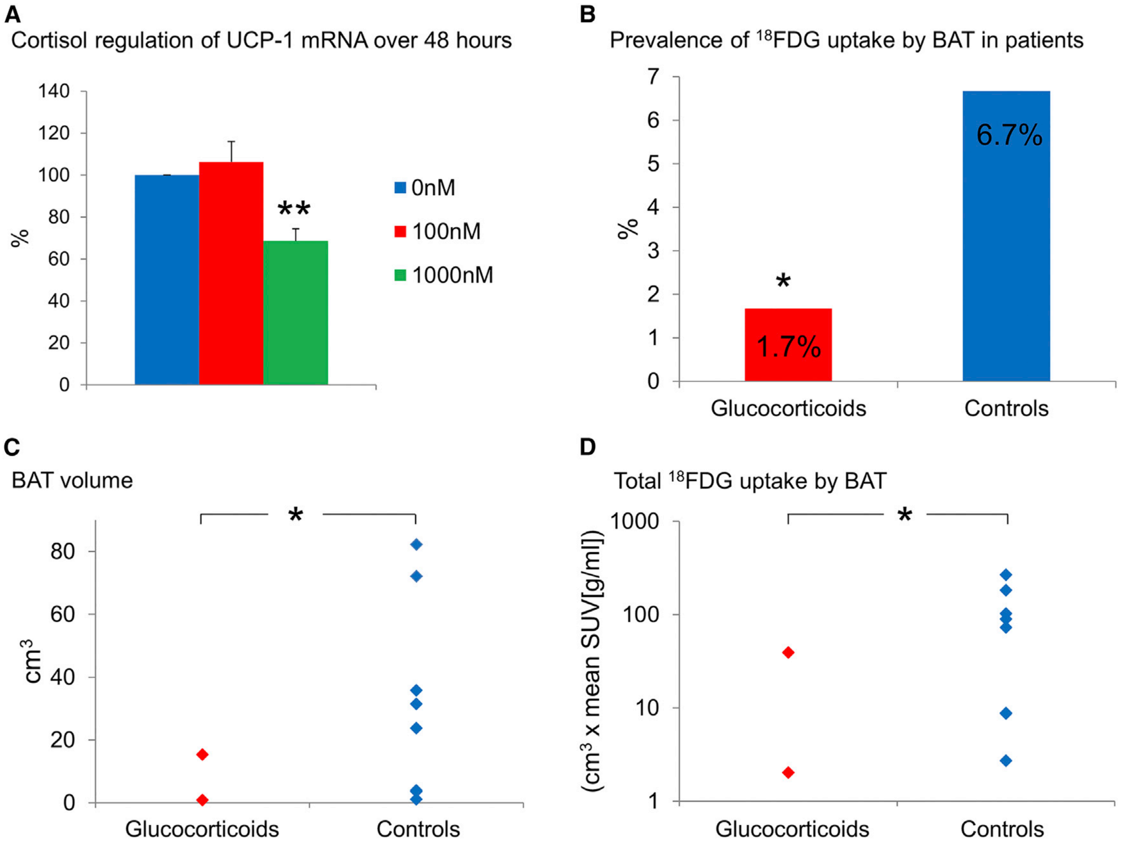

Figure 6. Chronic Glucocorticoid Use Suppresses BAT Activation in Humans

(A) UCP-1 mRNA levels from paired human brown adipocytes (n = 9) following 48 hr incubation with either 0 (blue columns), 100 (red columns), or 1,000 nM (green columns) cortisol with 0 nM normalized to 100%. Data were analyzed by repeated-measures ANOVA with post hoc Bonferroni testing. Data are mean ± SEM.

(B) The prevalence of ^{18}FDG uptake by BAT (PET/CT scanning performed 1 hr following a 400 MBq ^{18}FDG injection at room temperature [20°C–21°C]) in patients prescribed oral glucocorticoids (red column) or in matched controls (blue column). Data were analyzed by chi-square test.

(C and D) (C) The volume of active BAT (^{18}FDG uptake with SUV $\geq$ 2.0) and (D) the total ^{18}FDG uptake by BAT in glucocorticoid treated (red diamonds) and control (blue diamonds) patients with detectable ^{18}FDG uptake by BAT. BAT volume and total ^{18}FDG uptake by BAT were reduced in glucocorticoid-treated patients. Data were analyzed using the Mann-Whitney U test. *$p < 0.05$, **$p < 0.01$ versus control/0 nM.

temperature gradient (data not shown) because the chest temperature did not change between phases.

In our study, the increased levels of UCP-1 were not maintained following 48 hr of glucocorticoid treatment, while our chronic PET/CT data reveal that chronic glucocorticoid excess in fact suppresses BAT activation. While acute elevations of cortisol represent an adaptive stress response, chronic glucocorticoid excess is maladaptive and results in weight gain, insulin resistance, and dyslipidemia (Macfarlane et al., 2008). Therefore, suppression of BAT activation potentially contributes to these adverse metabolic consequences of chronic glucocorticoid treatment. It is also interesting to speculate how our findings relate to the reduced BAT mass/activation observed in obesity (van Marken Lichtenbelt et al., 2009). The majority of studies find peak plasma cortisol levels to be reduced in obesity (Rask et al., 2002; Strain et al., 1982; Phillips et al., 2000), likely due to increased cortisol clearance by the hepatic A-ring reductases (Andrew et al., 1998), which could result in reduced BAT cortisol concentrations, leading to reduced thermogenesis. In addition, we found a positive correlation between the cortisol-inactivating enzyme 11β-HSD2 and BMI in human BAT; however, 11β-HSD2 levels in both WAT and BAT are low, and it is unclear whether this would substantially reduce cortisol concentrations in BAT in vivo. An unexpected finding in this study was the significantly elevated 11β-HSD1 and reduced 11β-HSD2 mRNA levels seen in the brown adipocytes compared with the white adipocytes. These

(E) UCP-1 mRNA levels from paired primary human brown adipocytes (n = 7) following 24 hr incubation with cortisol at 0, 100, and 1,000 nM or equivalent concentrations (3.75 [pale red columns] and 37.5 nM [pale green columns]) of the selective glucocorticoid (GC) receptor (GR) agonist dexamethasone (Dex) ± the mineralocorticoid receptor (MR) antagonist eplerenone (10 μM). Low-dose cortisol and dexamethasone increased UCP-1 mRNA levels, while eplerenone did not alter UCP-1.

(F) UCP-1 mRNA levels from paired interscapular murine brown adipocytes (n = 6) following 4 and 8 hr incubation with 0, 25 (yellow columns), 50 (orange columns), 100, and 1,000 nM cortisol. Low- and high-dose cortisol suppressed UCP-1 following 8 hr incubation. Data were analyzed by repeated-measures ANOVA with post hoc Bonferroni testing. *$p < 0.05$, **$p < 0.01$ versus 0 nM; \$p < 0.05, \$\$p < 0.01 versus 100 nM cortisol.

differences were not observed in the whole tissue, so this may be caused by increased sensitivity in the brown adipocytes to unknown factors in the culture medium, such as macronutrients that can induce 11β-HSD1 (Stimson et al., 2014) or glucocorticoids that can suppress 11β-HSD2 (Lee et al., 2008).

We examined several genes known to be important in BAT function to determine the likely cause of the increased thermogenesis and the species-specific regulation by glucocorticoids. While glucocorticoids increased PGC-1α and GLUT-4 levels in human brown adipocytes, these were also induced in murine inguinal adipocytes and so are not responsible for these differences in BAT function. Mitochondrial markers were not increased, indicating that glucocorticoids likely do not improve general mitochondrial activity. However, the clear differential regulation of the key thermogenic protein UCP-1 between human and murine cells that importantly paralleled the differences in isoprenaline-induced respiration likely explains the clear differences between species, although the reason for this differential regulation is unclear. Well-known glucocorticoid-regulated genes such as HSL, ATGL, and PER1 were induced in both murine and human adipocytes, meaning that glucocorticoids were activating the GR signaling pathway in both species. GR binding in BAT has not been examined to date, so it is unclear if GR binds directly to the UCP-1 promoter region to enhance transcription. It appears unlikely that this effect is mediated through ZFP516, as this was unaltered in both species. In addition, prednisolone increased NEFA concentrations, which may indirectly enhance UCP-1 activation in BAT (Shabalina et al., 2004); however, ATGL and HSL (key genes in lipolysis) were increased in both species, while in vitro only 1,000 nM cortisol increased glycerol release (a marker of lipolysis) in the medium (data not shown) without increasing isoprenaline-stimulated oxygen consumption, so it is unlikely that this mechanism drives the increased BAT activation. Although the reason for the differential regulation of UCP-1 between species is unclear, ACTH (which rises during normal physiological stress) increases BAT activation in rodents (van den Beukel et al., 2014). This may partially counteract the suppressive effect of glucocorticoids, while acute stress enhances BAT thermogenesis in rats (Kataoka et al., 2014).

To conclude, we have shown that glucocorticoids acutely increase BAT activity in humans at least in part by activating UCP-1. This study highlights important species-specific differences in the regulation of BAT activation that may have important implications for the translation of novel therapeutic strategies designed to activate BAT to improve metabolic health.

EXPERIMENTAL PROCEDURES

Protocols for In Vivo Studies of BAT Activity

Glucocorticoid Regulation of Cold-Induced ^{18}FDG Uptake by Human BAT

Six men were recruited to a double-blind, randomized crossover study. Inclusion criteria were as follows: aged 18–35 years, BMI 18.5–25 kg/m^2, no acute or chronic medical conditions, on no regular medications, alcohol intake ≤21 units per week, no claustrophobia, and normal screening blood tests (full blood count, glucose, kidney, liver, and thyroid function). Participants were randomized to receive three doses of 10 mg prednisolone or placebo 12 hr apart prior to each study visit (at 0800 hr and 2000 hr the day prior to each study visit and at 0800 hr on the morning of the study visit). Volunteers were instructed to avoid alcohol or exercise for 48 hr prior to each visit. Volunteers attended the Clinical Research Facility at the Royal Infirmary of Edinburgh after overnight fast in standard light clothing and wore identical clothing at each visit. Subjects were placed in a room at 23°C–24°C (warm room), and measurements were performed of height, weight, fat mass, and blood pressure, and fasting blood samples were collected for glucose, insulin, ACTH, cortisol, and NEFAs.

An infusion of 6,6-[^{2}H]$_2$-glucose was commenced at 0.22 μmol/kg/min for 180 min following an initial bolus of 17.6 μmol/kg (Figure 1A). Volunteers then remained at rest in the warm room for 2 hr. Thereafter, subjects were transferred to the Clinical Research Imaging Centre and were placed supine in a room cooled to 17°C (cold room) for 2 hr. Subjects were checked every 15 min for signs or symptoms of shivering. Following 1 hr in the cold room, subjects were given an intravenous injection of 75 MBq ^{18}FDG. The PET scan commenced 1 hr following the ^{18}FDG injection, after a CT scan for attenuation correction. Subjects were then allowed to return home and attended for a second visit after at least 2 weeks washout. All studies involving human participants were reviewed and approved by the South East Scotland Research Ethics Committee, and informed consent was obtained from all participants.

Glucocorticoid Regulation of Cold-Induced Thermogenesis and Heat Production by Human BAT

Nine lean, healthy subjects (four female, five male) were recruited to a double-blind, randomized crossover study. Inclusion criteria were identical to the PET study except for additional inclusion criteria for the female participants: no current pregnancy and alcohol intake ≤14 units per week. Participants were randomized to receive identical dosing as in the PET study, taking three doses of 10 mg prednisolone or placebo 12 hr apart prior to each study visit, and the study protocol was identical to the PET study except for the following differences. Subjects were placed on a bed in a warm room (23°C–24°C) at t = 0 min for the first 2 hr (Figure 1B). At t + 110 min, subjects placed their hands in cold water (15°C) in an attempt to activate their BAT (Symonds et al., 2012). At t + 120 min, volunteers were moved to a room cooled to 16°C (cold room) for a further 2 hr to more robustly activate their BAT. Every 20 min from t = 0 onward, each subject's peripheral temperature was measured using a temperature probe applied to the dorsum of the hand (YSI 409 series, Henleys Medical Supplies Ltd), while thermal imaging was performed of the neck and upper body region. Energy expenditure was measured each hour using indirect calorimetry. At the end of the 4 hr protocol, subjects were allowed to return home and attended for their second visit either 2 weeks (in males) or 4 weeks (in females, to ensure menstrual cycle in the same phase at each visit) later to allow adequate washout between phases.

PET/CT Scanning Protocol and Analysis

All subjects were placed supine in a hybrid PET/CT scanner (Biograph mCT, Siemens Medical Systems). Subjects underwent an initial low-dose CT for attenuation correction (non-enhanced, 120 kV) with tube current modulation applied (20 [in healthy volunteers] or 50 [in patients] mAs quality reference) followed by static PET imaging of the upper body using 10 (volunteers) or 3 (patients) min beds. Images were analyzed using PMOD version 3.409 (PMOD technologies). ^{18}FDG uptake by BAT was quantified by measuring the mean standard uptake value (SUV) from all pixels with an SUV of greater than 2-fold background (>2.0), which corresponded to tissues with a radio density on the CT scan with Hounsfield unit (HU) values within the expected range for adipose tissue (from −150 to −30 HU). The total ^{18}FDG uptake by BAT was calculated as the mean SUV multiplied by the volume of active BAT.

Thermal Imaging

Thermal imaging was performed using a FLIR T650sc infrared camera. The camera was placed 1 m from the subject and the subject's upper body was photographed at the intervals described in Figure 1B. Identically sized regions of interest were drawn around the right and left supraclavicular regions and the anterior chest as shown in Figure 3A using Research IR version 4 (FLIR). The mean and maximum supraclavicular (left and right) and chest temperatures were recorded from each image, and the mean values from all images taken during each condition (warm, cold water, and cold exposure) are presented in the results. The results using the mean of the mean and the mean of the maximum were similar; as such, only the mean of the mean is presented.

Indirect Calorimetry

Energy expenditure was measured for 15 min each hour using a ventilated-hood indirect calorimeter (GEM Nutrition). The first 5 min of data were discarded and the mean value for the final 10 min recorded each hour. Energy expenditure (EE) is presented as the mean of two values obtained during

warm and cold exposure. Cold-induced thermogenesis was calculated by subtracting the mean EE in the cold room from the EE in the warm room.

Glucose Kinetics

Endogenous and 6,6-[^{2}H]$_2$-glucose were measured using liquid chromatography-mass spectrometry as previously described (Macfarlane et al., 2014). The mean concentration of D2-glucose at room temperature (from t + 90 to t + 120 min) and during the first hour of cold exposure (from t + 150 to 180 min) was used to calculate the metabolic clearance rate (MCR) of glucose using the equation MCR = D2-glucose infusion rate/ [mean D2-glucose].

Biochemical Assays

ACTH, insulin (DRG Instruments), and noradrenaline concentrations (LDN) were measured using commercially available ELISA kits. NEFAs were measured using a colorimetric assay (Wako Diagnostics). Cortisol was measured by liquid chromatography-mass spectrometry as previously described (Stimson et al., 2009).

In Vitro Studies of BAT Activity

Detailed in the Supplemental Information.

Statistical Analysis

Data are presented as mean ± SEM. Comparisons between two related groups were examined using the paired t test for normally distributed data, and using the Wilcoxon signed-rank test for data not normally distributed. Comparisons involving three or more groups were analyzed using repeated-measures ANOVA with post hoc testing using Bonferroni correction. Associations were tested using Pearson's correlation coefficient with Bonferroni correction. Data were tested for normal distribution using the one-sample Kolmogorov-Smirnov test. $p < 0.05$ was considered significant. Data were analyzed using SPSS version 19.

SUPPLEMENTAL INFORMATION

Supplemental Information includes Supplemental Experimental Procedures, five figures, and four tables and can be found with this article online at http://dx.doi.org/10.1016/j.cmet.2016.06.011.

AUTHOR CONTRIBUTIONS

R.H.S. designed the studies in conjunction with L.E.R., M.A., A.M.F., E.J.R.B., N.M.M., and B.R.W. L.E.R., M.A., A.M.F., J.F., M.N., R.N.C., N.M.M., and R.H.S. conducted the experiments and/or analyzed data. R.H.S. wrote the initial draft of the manuscript, and all authors critically reviewed the manuscript.

ACKNOWLEDGMENTS

This work was supported by grants from the Medical Research Council (R.H.S., MR/K010271/1), Wellcome Trust, and the Society for Endocrinology. B.R.W. is supported by grants from the Wellcome Trust and the British Heart Foundation. R.N.C. and N.M.M. were supported by a New Investigator Award from the Wellcome Trust (N.M.M., 100981/Z/13/Z). The authors acknowledge the financial support of NHS Research Scotland (NRS), through the Edinburgh Clinical Research Facility and, in particular, Jo-Anne Robertson and Lucy Marshall, and the British Heart Foundation Centre of Research Excellence. We also thank Karen French, Maria-Lena Gregoriades, Natalie Homer, Sanjay-kumar Kothiya, Hamish Richardson, and Gemma Roberts for their technical assistance.

Received: December 11, 2015
Revised: May 6, 2016
Accepted: June 15, 2016
Published: July 12, 2016

REFERENCES

Andrew, R., Phillips, D.I.W., and Walker, B.R. (1998). Obesity and gender influence cortisol secretion and metabolism in man. J. Clin. Endocrinol. Metab. *83*, 1806–1809.

Barclay, J.L., Agada, H., Jang, C., Ward, M., Wetzig, N., and Ho, K.K. (2015). Effects of glucocorticoids on human brown adipocytes. J. Endocrinol. *224*, 139–147.

Boonen, E., Vervenne, H., Meersseman, P., Andrew, R., Mortier, L., Declercq, P.E., Vanwijngaerden, Y.M., Spriet, I., Wouters, P.J., Vander Perre, S., et al. (2013). Reduced cortisol metabolism during critical illness. N. Engl. J. Med. *368*, 1477–1488.

Cypess, A.M., Lehman, S., Williams, G., Tal, I., Rodman, D., Goldfine, A.B., Kuo, F.C., Palmer, E.L., Tseng, Y.H., Doria, A., et al. (2009). Identification and importance of brown adipose tissue in adult humans. N. Engl. J. Med. *360*, 1509–1517.

Cypess, A.M., White, A.P., Vernochet, C., Schulz, T.J., Xue, R., Sass, C.A., Huang, T.L., Roberts-Toler, C., Weiner, L.S., Sze, C., et al. (2013). Anatomical localization, gene expression profiling and functional characterization of adult human neck brown fat. Nat. Med. *19*, 635–639.

Cypess, A.M., Weiner, L.S., Roberts-Toler, C., Franquet Elía, E., Kessler, S.H., Kahn, P.A., English, J., Chatman, K., Trauger, S.A., Doria, A., and Kolodny, G.M. (2015). Activation of human brown adipose tissue by a β3-adrenergic receptor agonist. Cell Metab. *21*, 33–38.

Dempersmier, J., Sambeat, A., Gulyaeva, O., Paul, S.M., Hudak, C.S., Raposo, H.F., Kwan, H.Y., Kang, C., Wong, R.H., and Sul, H.S. (2015). Cold-inducible Zfp516 activates UCP1 transcription to promote browning of white fat and development of brown fat. Mol. Cell *57*, 235–246.

Hardwick, A.J., Linton, E.A., and Rothwell, N.J. (1989). Thermogenic effects of the antiglucocorticoid RU-486 in the rat: involvement of corticotropin-releasing factor and sympathetic activation of brown adipose tissue. Endocrinology *124*, 1684–1688.

Hughes, K.A., Reynolds, R.M., Andrew, R., Critchley, H.O., and Walker, B.R. (2010). Glucocorticoids turn over slowly in human adipose tissue in vivo. J. Clin. Endocrinol. Metab. *95*, 4696–4702.

Jang, C., Jalapu, S., Thuzar, M., Law, P.W., Jeavons, S., Barclay, J.L., and Ho, K.K. (2014). Infrared thermography in the detection of brown adipose tissue in humans. Physiol. Rep. *2*, e12167.

Jespersen, N.Z., Larsen, T.J., Peijs, L., Daugaard, S., Homøe, P., Loft, A., de Jong, J., Mathur, N., Cannon, B., Nedergaard, J., et al. (2013). A classical brown adipose tissue mRNA signature partly overlaps with brite in the supraclavicular region of adult humans. Cell Metab. *17*, 798–805.

Kataoka, N., Hioki, H., Kaneko, T., and Nakamura, K. (2014). Psychological stress activates a dorsomedial hypothalamus-medullary raphe circuit driving brown adipose tissue thermogenesis and hyperthermia. Cell Metab. *20*, 346–358.

Kong, X., Yu, J., Bi, J., Qi, H., Di, W., Wu, L., Wang, L., Zha, J., Lv, S., Zhang, F., et al. (2015). Glucocorticoids transcriptionally regulate miR-27b expression promoting body fat accumulation via suppressing the browning of white adipose tissue. Diabetes *64*, 393–404.

Kraan, G.P., Dullaart, R.P., Pratt, J.J., Wolthers, B.G., Drayer, N.M., and De Bruin, R. (1998). The daily cortisol production reinvestigated in healthy men. The serum and urinary cortisol production rates are not significantly different. J. Clin. Endocrinol. Metab. *83*, 1247–1252.

Lee, M.J., Fried, S.K., Mundt, S.S., Wang, Y., Sullivan, S., Stefanni, A., Daugherty, B.L., and Hermanowski-Vosatka, A. (2008). Depot-specific regulation of the conversion of cortisone to cortisol in human adipose tissue. Obesity (Silver Spring) *16*, 1178–1185.

Lee, P., Ho, K.K., Lee, P., Greenfield, J.R., Ho, K.K., and Greenfield, J.R. (2011). Hot fat in a cool man: infrared thermography and brown adipose tissue. Diabetes Obes. Metab. *13*, 92–93.

Lee, P., Bova, R., Schofield, L., Bryant, W., Dieckmann, W., Slattery, A., Govendir, M.A., Emmett, L., and Greenfield, J.R. (2016). Brown adipose tissue exhibits a glucose-responsive thermogenic biorhythm in humans. Cell Metab. *23*, 602–609.

Liu, J., Kong, X., Wang, L., Qi, H., Di, W., Zhang, X., Wu, L., Chen, X., Yu, J., Zha, J., et al. (2013). Essential roles of 11β-HSD1 in regulating brown adipocyte function. J. Mol. Endocrinol. *50*, 103–113.

Macfarlane, D.P., Forbes, S., and Walker, B.R. (2008). Glucocorticoids and fatty acid metabolism in humans: fuelling fat redistribution in the metabolic syndrome. J. Endocrinol. *197*, 189–204.

Macfarlane, D.P., Raubenheimer, P.J., Preston, T., Gray, C.D., Bastin, M.E., Marshall, I., Iredale, J.P., Andrew, R., and Walker, B.R. (2014). Effects of acute glucocorticoid blockade on metabolic dysfunction in patients with type 2 diabetes with and without fatty liver. Am. J. Physiol. Gastrointest. Liver Physiol. *307*, G760–G768.

Ouellet, V., Labbé, S.M., Blondin, D.P., Phoenix, S., Guérin, B., Haman, F., Turcotte, E.E., Richard, D., and Carpentier, A.C. (2012). Brown adipose tissue oxidative metabolism contributes to energy expenditure during acute cold exposure in humans. J. Clin. Invest. *122*, 545–552.

Phillips, D.I.W., Walker, B.R., Reynolds, R.M., Flanagan, D.E.H., Wood, P.J., Osmond, C., Barker, D.J.P., and Whorwood, C.B. (2000). Low birth weight predicts elevated plasma cortisol concentrations in adults from 3 populations. Hypertension *35*, 1301–1306.

Rask, E., Walker, B.R., Söderberg, S., Livingstone, D.E., Eliasson, M., Johnson, O., Andrew, R., and Olsson, T. (2002). Tissue-specific changes in peripheral cortisol metabolism in obese women: increased adipose 11beta-hydroxysteroid dehydrogenase type 1 activity. J. Clin. Endocrinol. Metab. *87*, 3330–3336.

Rodríguez, A.M., and Palou, A. (2004). The steroid RU486 induces UCP1 expression in brown adipocytes. Pflugers Arch. *449*, 170–174.

Saito, M., Okamatsu-Ogura, Y., Matsushita, M., Watanabe, K., Yoneshiro, T., Nio-Kobayashi, J., Iwanaga, T., Miyagawa, M., Kameya, T., Nakada, K., et al. (2009). High incidence of metabolically active brown adipose tissue in healthy adult humans: effects of cold exposure and adiposity. Diabetes *58*, 1526–1531.

Shabalina, I.G., Jacobsson, A., Cannon, B., and Nedergaard, J. (2004). Native UCP1 displays simple competitive kinetics between the regulators purine nucleotides and fatty acids. J. Biol. Chem. *279*, 38236–38248.

Shinoda, K., Luijten, I.H., Hasegawa, Y., Hong, H., Sonne, S.B., Kim, M., Xue, R., Chondronikola, M., Cypess, A.M., Tseng, Y.H., et al. (2015). Genetic and functional characterization of clonally derived adult human brown adipocytes. Nat. Med. *21*, 389–394.

Sidossis, L., and Kajimura, S. (2015). Brown and beige fat in humans: thermogenic adipocytes that control energy and glucose homeostasis. J. Clin. Invest. *125*, 478–486.

Stimson, R.H., Andersson, J., Andrew, R., Redhead, D.N., Karpe, F., Hayes, P.C., Olsson, T., and Walker, B.R. (2009). Cortisol release from adipose tissue by 11beta-hydroxysteroid dehydrogenase type 1 in humans. Diabetes *58*, 46–53.

Stimson, R.H., Mohd-Shukri, N.A., Bolton, J.L., Andrew, R., Reynolds, R.M., and Walker, B.R. (2014). The postprandial rise in plasma cortisol in men is mediated by macronutrient-specific stimulation of adrenal and extra-adrenal cortisol production. J. Clin. Endocrinol. Metab. *99*, 160–168.

Strack, A.M., Bradbury, M.J., and Dallman, M.F. (1995). Corticosterone decreases nonshivering thermogenesis and increases lipid storage in brown adipose tissue. Am. J. Physiol. *268*, R183–R191.

Strain, G.W., Zumoff, B., Kream, J., Strain, J.J., Levin, J., and Fukushima, D. (1982). Sex difference in the influence of obesity on the 24 hr mean plasma concentration of cortisol. Metabolism *31*, 209–212.

Symonds, M.E., Henderson, K., Elvidge, L., Bosman, C., Sharkey, D., Perkins, A.C., and Budge, H. (2012). Thermal imaging to assess age-related changes of skin temperature within the supraclavicular region co-locating with brown adipose tissue in healthy children. J. Pediatr. *161*, 892–898.

van den Beukel, J.C., Grefhorst, A., Quarta, C., Steenbergen, J., Mastroberardino, P.G., Lombès, M., Delhanty, P.J., Mazza, R., Pagotto, U., van der Lely, A.J., and Themmen, A.P. (2014). Direct activating effects of adrenocorticotropic hormone (ACTH) on brown adipose tissue are attenuated by corticosterone. FASEB J. *28*, 4857–4867.

van Marken Lichtenbelt, W.D., Vanhommerig, J.W., Smulders, N.M., Drossaerts, J.M., Kemerink, G.J., Bouvy, N.D., Schrauwen, P., and Teule, G.J. (2009). Cold-activated brown adipose tissue in healthy men. N. Engl. J. Med. *360*, 1500–1508.

Virtanen, K.A., Lidell, M.E., Orava, J., Heglind, M., Westergren, R., Niemi, T., Taittonen, M., Laine, J., Savisto, N.J., Enerbäck, S., and Nuutila, P. (2009). Functional brown adipose tissue in healthy adults. N. Engl. J. Med. *360*, 1518–1525.

Wang, W., Kissig, M., Rajakumari, S., Huang, L., Lim, H.W., Won, K.J., and Seale, P. (2014). Ebf2 is a selective marker of brown and beige adipogenic precursor cells. Proc. Natl. Acad. Sci. USA *111*, 14466–14471.

Wilson, O., Hedner, P., Laurell, S., Nosslin, B., Rerup, C., and Rosengren, E. (1970). Thyroid and adrenal response to acute cold exposure in man. J. Appl. Physiol. *28*, 543–548.

Wu, J., Boström, P., Sparks, L.M., Ye, L., Choi, J.H., Giang, A.H., Khandekar, M., Virtanen, K.A., Nuutila, P., Schaart, G., et al. (2012). Beige adipocytes are a distinct type of thermogenic fat cell in mouse and human. Cell *150*, 366–376.

Cell Metabolism, Volume *24*

Supplemental Information

Glucocorticoids Acutely Increase Brown Adipose Tissue Activity in Humans, Revealing Species-Specific Differences in UCP-1 Regulation

Lynne E. Ramage, Murat Akyol, Alison M. Fletcher, John Forsythe, Mark Nixon, Roderick N. Carter, Edwin J.R. van Beek, Nicholas M. Morton, Brian R. Walker, and Roland H. Stimson

Supplemental Information

Figure S1 (related to Experimental Procedures) Anatomical location of intra-operative collection of human brown adipose tissue

A) CT and B) PET/CT fused axial images of the neck from a healthy subject. Intra-operative samples of brown adipose tissue were obtained from the deep supraclavicular region posterior to the lateral thyroid and adjacent to the longus colli muscle, highlighted by the arrow in panel B identifying the adipose tissue with substantial cold-induced ^{18}fluorodeoxyglucose uptake (blue/yellow areas) in that site. Paired white adipose tissue samples were simultaneously obtained from the superficial adipose tissue in the neck.

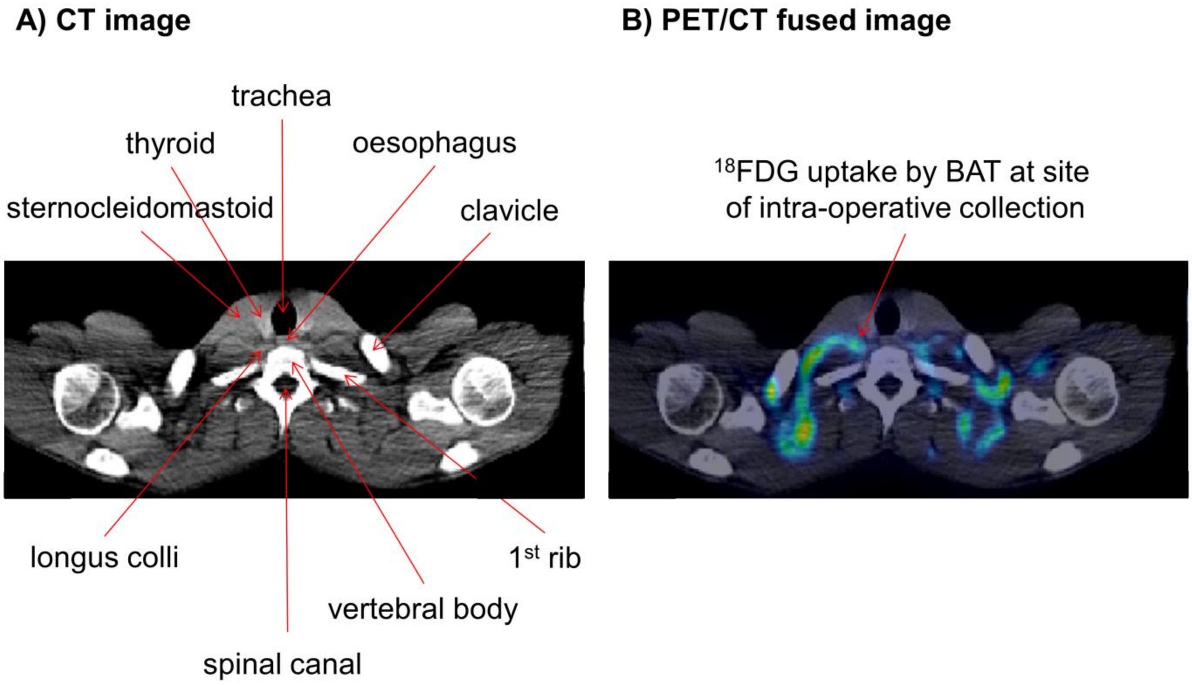

Figure S2 (related to Figure 4) Human BAT expresses markers of both beige and classic BAT

*Data are mean ± SEM (n=11) for mRNA levels from differentiated human pre-adipocytes cultured from the superficial (WAT, yellow columns) and the deeper (BAT, red columns) depots. The brown adipocytes expressed higher levels of the classic BAT markers Ebf3 and ZIC1 than the white adipocytes. In addition, the brown adipocytes had increased levels of the beige markers TMEM26 and Tbx1 but lower levels of HOXC9. The brown adipocytes tended to have lower levels of the WAT marker leptin than the white adipocytes (p=0.07). *P<0.05, **P<0.01 vs WAT.*

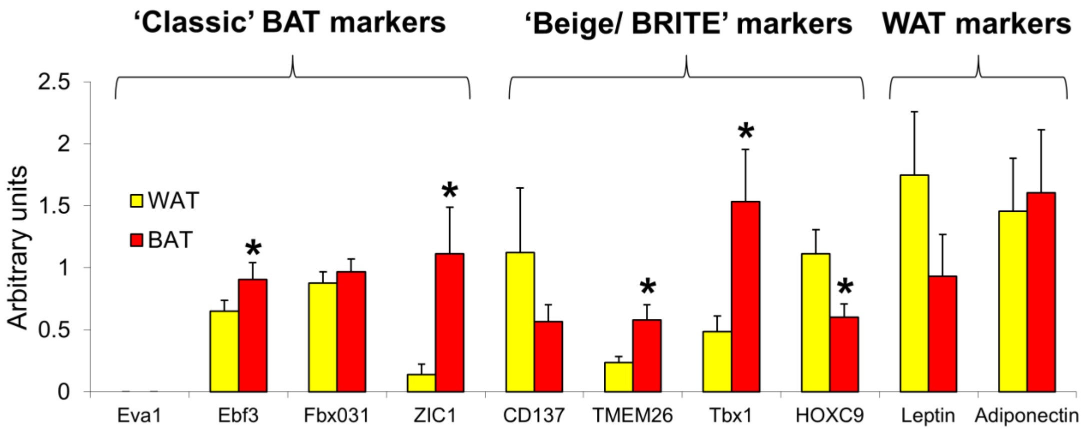

Figure S3 (related to Figure 5) Glucocorticoid regulation of human adipocytes

*A) Data are mean ± SEM for n=6 paired human white adipocytes cultured for 24 hours in either 0 (blue columns), 100 (red columns) or 1000nM (green columns) cortisol, with basal 0nM normalised to 100%. Cortisol did not alter either basal or isoprenaline-stimulated respiration. B-D) mRNA levels (with 0nM normalised to 100%) from paired B,D) white and C) brown adipocytes (n=8) following 24 hours incubation with cortisol 0, 100 and 1000nM. B) Cortisol did not alter UCP-1 transcript levels in the white adipocytes. mRNA levels of β3-adrenoreceptor (β3-AR) were below the limit of detection in the white adipocytes. Cortisol increased PGC-1α in white adipocytes and C,D) increased classic glucocorticoid (GC)-regulated genes such as hormone sensitive lipase (HSL), adipose triglyceride lipase (ATGL) and Per1 in brown and white adipocytes. *P<0.05, **P<0.01 vs 0nM.*

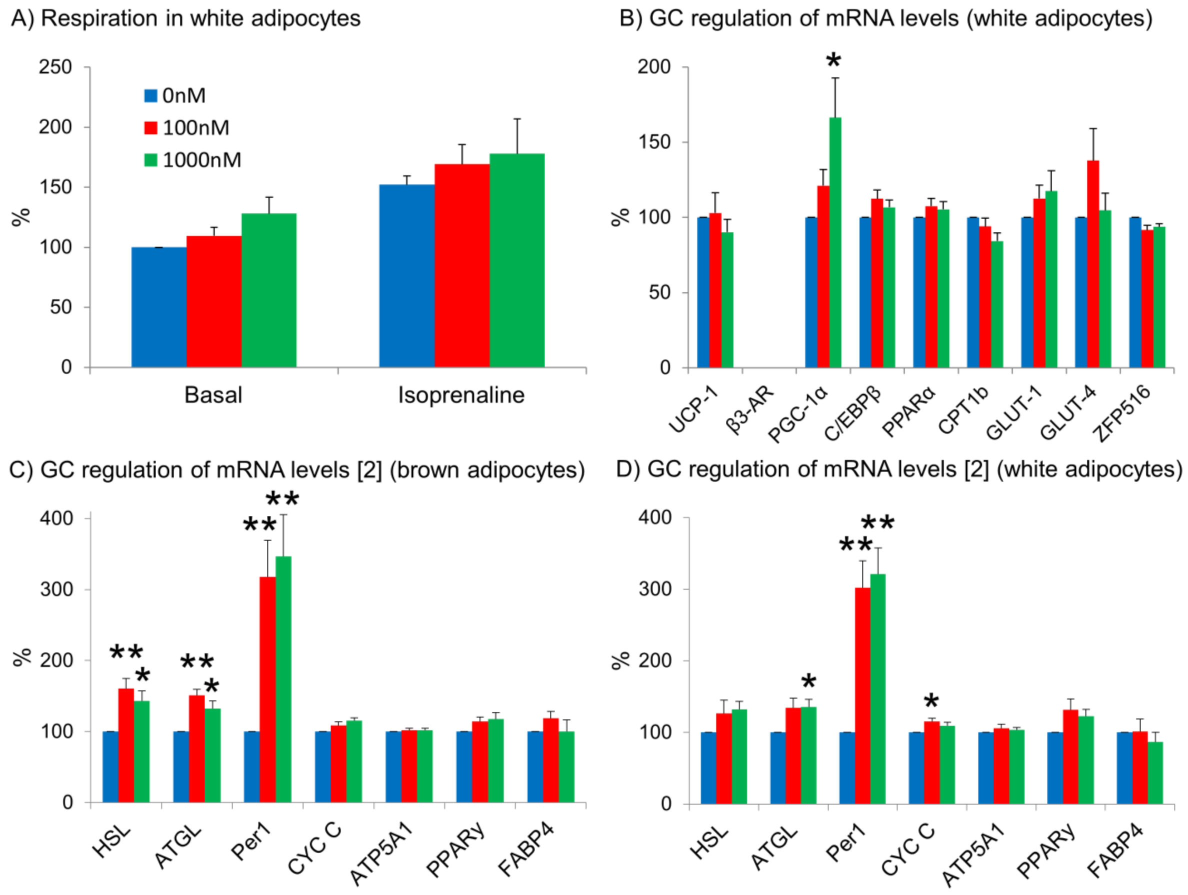

Figure S4 (related to Figure 5) Glucocorticoid regulation of murine adipocytes

*A) Data mean ± SEM for mRNA levels from paired primary murine adipocytes from the epididymal (Epi, n=4), inguinal (Ing, n=6) and interscapular brown adipose tissue (iBAT, n=6) depots following 24 hours incubation with the glucocorticoid (GC) cortisol at 0 (blue columns), 100 (red columns) and 1000 nM (green columns). CL316,243 (1µM) was added to inguinal cells (Ing+CL) for 5 days following differentiation to induce browning. B-F) mRNA levels (with 0nM normalised to 100%) from murine B,D,E) inguinal (n=6) and C,F) interscapular brown adipocytes (n=6) following 24 hours incubation with 0, 100 and 1000nM cortisol. G,H) UCP-1 mRNA levels from paired murine G) unstimulated (n=7) and H) CL316,243 treated (n=7) inguinal adipocytes following 4 and 8 hours incubation with 0, 25 (yellow columns), 50 (orange columns), 100 and 1000nM cortisol. Data were analysed by repeated measures ANOVA with post-hoc Bonferroni correction. *P<0.05, **P<0.01 vs 0nM; $P<0.05 vs 100nM cortisol.*

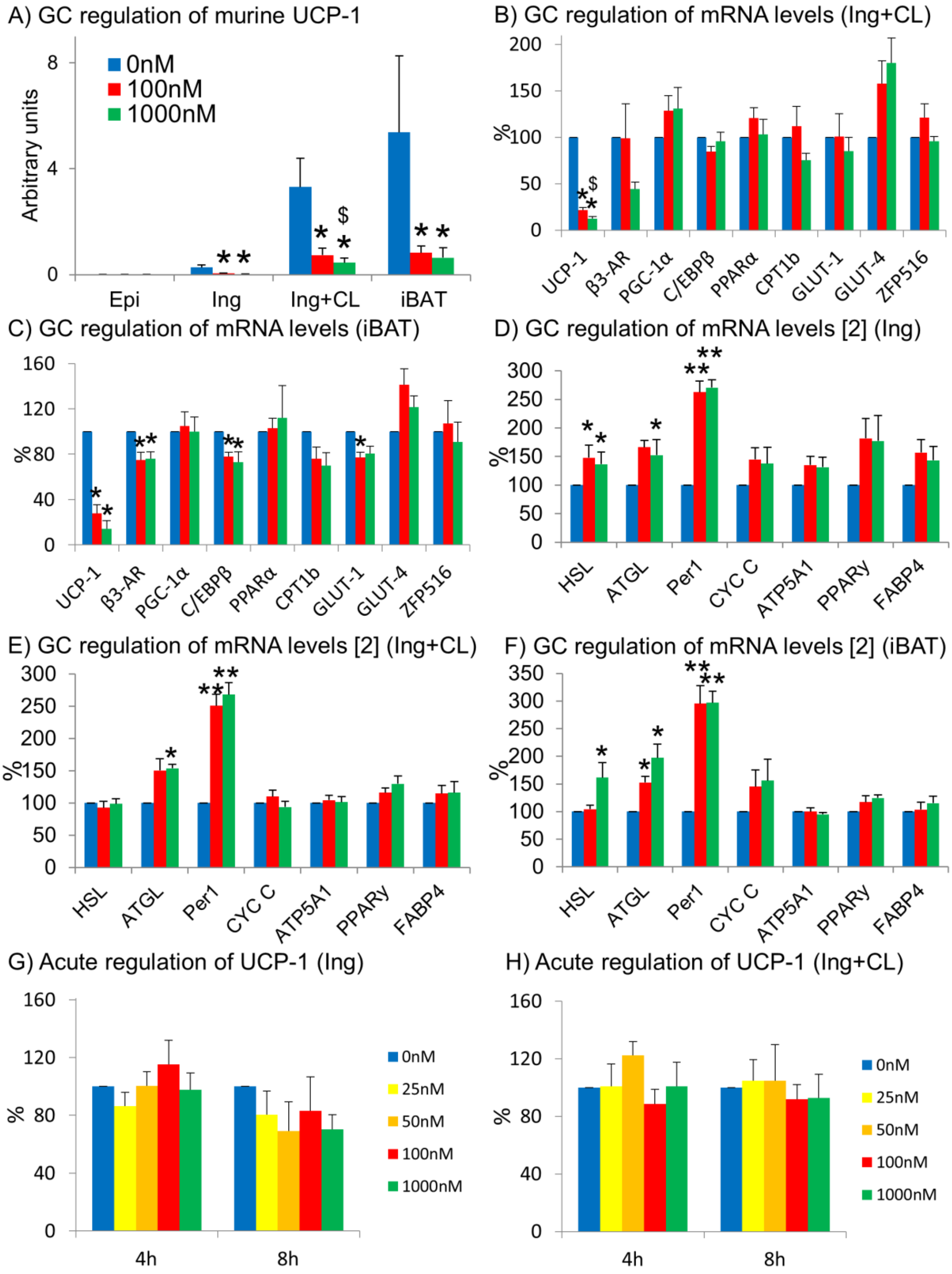

A) GC regulation of murine UCP-1
Arbitrary units
0nM
100nM
1000nM
Epi
Ing
Ing+CL
iBAT
B) GC regulation of mRNA levels (Ing+CL)
%
UCP-1
β3-AR
PGC-1α
C/EBPβ
PPARα
CPT1b
GLUT-1
GLUT-4
ZFP516
C) GC regulation of mRNA levels (iBAT)
%
UCP-1
β3-AR
PGC-1α
C/EBPβ
PPARα
CPT1b
GLUT-1
GLUT-4
ZFP516
D) GC regulation of mRNA levels [2] (Ing)
%
HSL
ATGL
Per1
CYC C
ATP5A1
PPARγ
FABP4
E) GC regulation of mRNA levels [2] (Ing+CL)
%
HSL
ATGL
Per1
CYC C
ATP5A1
PPARγ
FABP4
F) GC regulation of mRNA levels [2] (iBAT)
%
HSL
ATGL
Per1
CYC C
ATP5A1
PPARγ
FABP4
G) Acute regulation of UCP-1 (Ing)
%
0nM
25nM
50nM
100nM
1000nM
4h
8h
H) Acute regulation of UCP-1 (Ing+CL)
%
0nM
25nM
50nM
100nM
1000nM
4h
8h

Figure S5 (related to Figure 5) Glucocorticoid regulation of respiration

*A,B) Data are mean ± SEM (with basal 0nM normalised to 100%) for paired murine A) inguinal CL316,243 treated (n=7) and B) interscapular brown adipocytes (n=7) incubated for 24 hours with either 0 (blue columns), 100 (red columns) or 1000nM (green columns) cortisol. Cortisol (100nM and 1000nM) decreased isoprenaline-stimulated oxygen consumption in the murine adipocytes. Data were analysed by repeated measures ANOVA with post-hoc Bonferroni testing. *P<0.05 vs 0nM. C,D) Representative respirometry traces from C) human white (dotted lines) and brown (solid lines) and D) murine inguinal 'beige' adipocytes following incubation for 24 hours with either 0 (blue lines), 100 (red lines) or 1000nM (green lines) cortisol. Samples were all analysed in triplicate.*

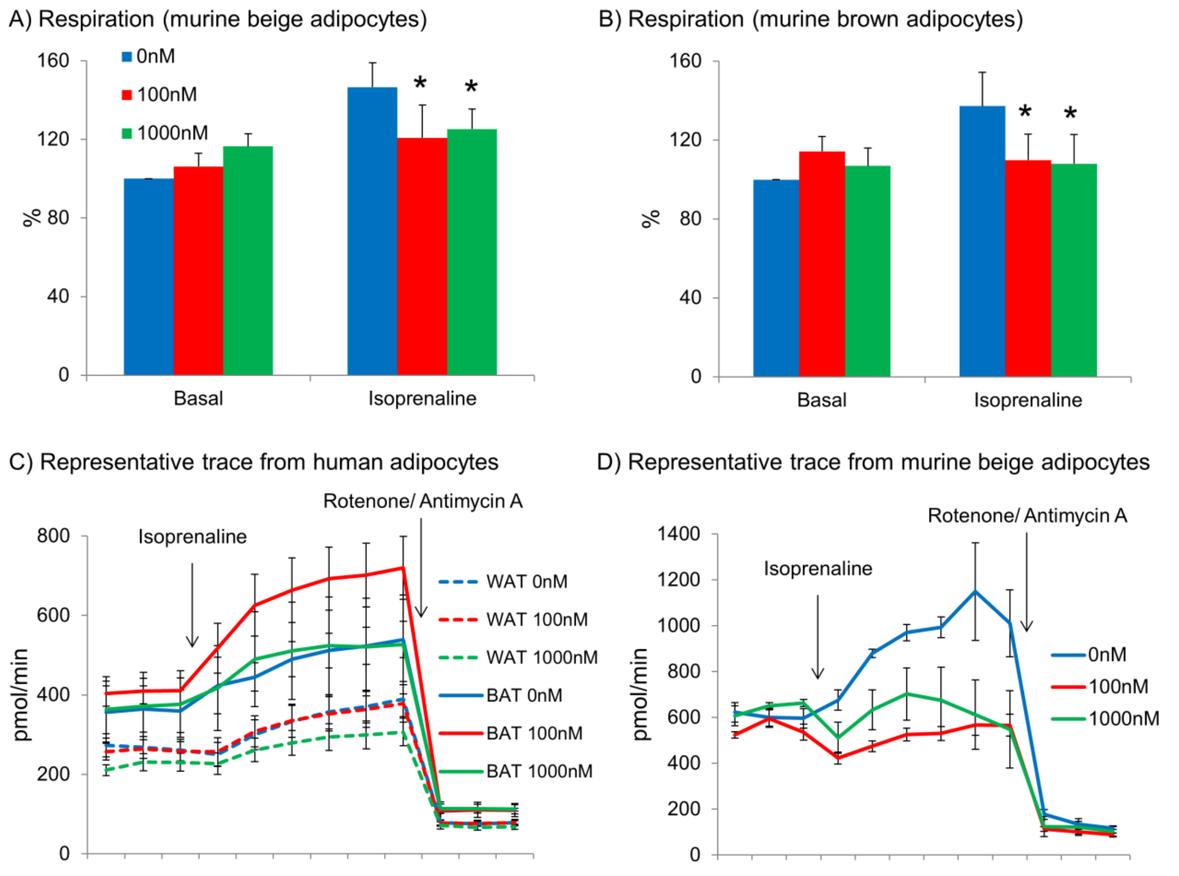

Table S1 (related to Experimental Procedures) Average outdoor environmental temperatures

Data are the mean maximum and minimum outdoor temperatures during the months when the two in vivo human studies were performed. Historical data were obtained from the Met Office Leuchars station which is situated closest to Edinburgh, Scotland, United Kingdom (1).

	**Maximum temperature (˚C)**	**Minimum temperature (˚C)**
**PET/CT study**		
March	13.3	3.7
April	10.1	2.7
May	12.5	5.3
June	14.5	8.6
July	17.1	10.3
August	18.6	11.3
September	16.2	8.0
**Thermal imaging study**		
January	6.4	0.3
February	7.8	0.5
March	9.7	2.1
April	13.3	2.7
May	14.0	5.0

Table S2 (related to Experimental Procedures) Participants for human in vitro experiments

**Experiment/ Subject number**	**Operation**	**Underlying diagnosis**	**Age**	**Sex**	**BMI (kg/m^2)**
**Whole tissue mRNA and histology**					
**1**	Parathyroidectomy	Primary hyperparathyroidism	52	Female	27.0
**2**	Parathyroidectomy	Primary hyperparathyroidism	70	Female	28.3
**3**	Parathyroidectomy	Primary hyperparathyroidism	63	Female	33.2
**4**	Thyroid lobectomy	Papillary carcinoma of thyroid	45	Female	26.5
**5**	Thyroid lobectomy	Hashimoto's thyroiditis	51	Female	30.7
**6**	Parathyroidectomy	Primary hyperparathyroidism	52	Female	47.1
**7**	Thyroid lobectomy	Follicular adenoma of thyroid	48	Female	17.6
**8**	Parathyroidectomy	Primary hyperparathyroidism	54	Male	33.3
**9**	Thyroid lobectomy	Benign hyperplastic thyroid nodule	39	Female	27.7
**Additional whole tissue for correlations**					
**1**	Thyroidectomy	Papillary carcinoma of thyroid	52	Female	39.4
**2**	Parathyroidectomy	Primary hyperparathyroidism	24	Male	27.7
**3**	Thyroid lobectomy	Benign hyperplastic nodule	46	Female	40.1
**4**	Thyroidectomy	Graves' disease	27	Female	30.1
**5**	Thyroid lobectomy	Multinodular goitre	37	Female	38.5
**6**	Thyroidectomy	Graves' disease	48	Female	32.3
**7**	Thyroid lobectomy	Follicular adenoma	27	Female	23.9
**8**	Thyroidectomy	Graves' disease	57	Female	25.8
**9**	Thyroid lobectomy	Multinodular goitre	66	Female	24.5
**10**	Thyroidectomy	Graves' disease	26	Female	20.9
**11**	Thyroidectomy	Graves' disease	36	Female	28.2
**12**	Parathyroidectomy	Primary hyperparathyroidism	62	Female	28.4
**Cultured adipocytes for mRNA and histology**					
**1**	Thyroidectomy	Graves' disease	21	Female	26.7
**2**	Thyroidectomy	Benign hyperplastic nodule	39	Female	27.8
**3**	Thyroid lobectomy	Benign hyperplastic nodule	41	Female	23.1
**4**	Parathyroidectomy	Primary hyperparathyroidism	57	Female	30.8
**5**	Parathyroidectomy	Primary hyperparathyroidism	66	Female	32.4
**6**	Thyroidectomy	Multinodular goitre	49	Male	33.2
**7**	Parathyroidectomy	Primary hyperparathyroidism	67	Female	32.0
**8**	Thyroid lobectomy	Benign hyperplastic nodule	55	Female	29.3
**9**	Parathyroidectomy	Primary hyperparathyroidism	34	Female	31.0
**10**	Parathyroidectomy	Primary hyperparathyroidism	58	Female	44.1
**11**	Parathyroidectomy	Primary hyperparathyroidism	42	Male	23.1

**Respirometry**					
**1**	Thyroid lobectomy	Toxic thyroid nodule	30	Male	20.2
**2**	Thyroid lobectomy	Follicular thyroid carcinoma	35	Male	38.9
**3**	Thyroidectomy	Graves' disease	25	Female	18.9
**4**	Parathyroidectomy	Primary hyperparathyroidism	48	Female	28.0
**5**	Thyroidectomy	Multinodular goitre	41	Female	45.7
**6**	Thyroidectomy	Graves' disease	31	Female	37.8
**24 hour incubation with glucocorticoids**					
**1**	Thyroid lobectomy	Benign thyroid nodule	41	Female	27.5
**2**	Parathyroidectomy	Hyperparathyroidism	59	Female	24.5
**3**	Thyroidectomy	Papillary thyroid carcinoma	52	Female	39.4
**4**	Thyroid lobectomy	Toxic thyroid adenoma	30	Male	20.2
**5**	Thyroidectomy	Toxic multinodular goitre	50	Female	22.3
**6**	Parathyroidectomy	Hyperparathyroidism	39	Female	31.7
**7**	Parathyroidectomy	Hyperparathyroidism	44	Male	37.6
**8**	Thyroid lobectomy	Follicular adenoma of thyroid	43	Male	26.2
**24 hour incubation +/- eplerenone**					
**1**	Thyroid lobectomy	Hyperplastic thyroid nodule	22	Female	28.7
**2**	Thyroidectomy	Graves' disease	42	Female	38.4
**3**	Thyroidectomy	Multinodular goitre	51	Female	36.1
**4**	Thyroid lobectomy	Colloid cyst	30	Female	25.4
**5**	Thyroidectomy	Multinodular goitre	57	Female	31.1
**6**	Parathyroidectomy	Hyperparathyroidism	58	Female	23.8
**7**	Thyroidectomy	Papillary thyroid carcinoma	31	Female	33.8
**48 hour incubation with glucocorticoids**					
**1**	Thyroid lobectomy	Normal thyroid	26	Female	25.4
**2**	Thyroid lobectomy	Multinodular goitre	48	Female	32.9
**3**	Thyroidectomy	Follicular carcinoma of thyroid	50	Female	26.0
**4**	Thyroidectomy	Papillary thyroid carcinoma	23	Female	20.9
**5**	Thyroid lobectomy	Hurthle cell adenoma	32	Female	20.3
**6**	Thyroid lobectomy	Follicular adenoma of thyroid	24	Male	21.6
**7**	Thyroidectomy	Multinodular goitre	22	Male	35.3
**8**	Thyroid lobectomy	Follicular adenoma of thyroid	45	Female	22.5
**9**	Thyroidectomy	Multinodular goitre	48	Female	31.1

Table S3 (related to Figure 6) Patient characteristics for retrospective analysis of PET/CT scans

Data are mean ± SEM. Anthropometric and biochemical data from patients who attended the Royal Infirmary of Edinburgh to undergo PET/CT scanning as part of their diagnostic pathway. All patients received 400MBq of ^{18}FDG 60 minutes prior to undergoing PET/CT scanning of their body. Patients were kept in a room at 20-21˚C prior to and following the ^{18}FDG injection until the PET/CT scan was performed. Data were analysed using the unpaired t test.

	**Controls**	**Glucocorticoid treated**
**Number of patients**	120	129
**Age (years)**	64.5 ± 1.4	63.5 ± 1.4
**Sex (female/ male)**	66/ 54	67/ 62
**Weight (kg)**	70.2 ± 1.3	69.7 ± 1.5
**Body mass index (kg/m^2)**	25.2 ± 0.4	25.0 ± 0.5
**Fasting plasma glucose (mmol/L)**	6.0 ± 0.1	5.9 ± 0.1

Table S4 (related to Experimental Procedures) Primer sequences for qPCR and corresponding probe numbers

All assays were performed using the following primer probe sequences and the Roche probe library, except for β3-adrenoreceptor which was measured using the Taqman assay ID Hs00609046_m1, while hormone sensitive lipase (LIPE) was measured using the SYBR green assay and the forward primer ggaagtgctatcgtctctgg and reverse primer ggcagtcagtggcatctc.

Gene Name	Primer sequences 5' to 3'	Roche UPL Probe number
**Human samples**		
UCP1	F: ctcaccgcagggaaagaa	25
	R: ggttgcccaatgaatactgc	
PRDM16	F: tggctgcttctggactca	63
	R: atattatttacaacgtcaccgtcact	
PPARGC1A (PGC-1α)	F: tgagagggccaagcaaag	13
	R: ataaatcacacggcgctctt	
DIO2 (de-iodinase type 2)	F: ggaagagcttcctcctcgat	47
	R: tccttctgtactggagacatgc	
NR3C1 (glucocorticoid receptor-α)	F: ttttcttcaaaagagcagtgga	11
	R: gcatgctgggcagttttt	
NR3C2 (mineralocorticoid receptor)	F: catcatgaaagttttgctgctact	64
	R: tctttgatgtaatttgtcctcatttc	
HSD11B1 (11β-HSD1)	F: caatggaagcattgttgtcg	20
	R: ggcagcaaccattggataag	
HSD11B2 (11β-HSD2)	F: gtcaaggtcagcatcatcca	71
	R: cactgacccacgtttctcac	
PPIA (cyclophilin A)	F: atgctggacccaacacaat	48
	R: tctttcactttgccaaacacc	
RNA18S5 (18S)	F: cttccacaggaggcctacac	46
	R: cgcaaaatatgctggaacttt	
MPZL2 (Eva1)	F: ggccgaaagagctcataaag	35
	R: cagagaccttttctcttggttg	
EBF3	F: gcacaacaattccaaacacg	70
	R: gatgcacggagtggcttc	
FBXO31	F: ccatacggaggactgctga	77
	R: gtacatccacccgatgatga	
ZIC1	F: atccacaaaaggacgcacac	7
	R: gtcacagccctcaaactcg	
CD137	F: cctgaagaccaaggagtgga	54
	R: gcaaagctgattccaagagaa	
TMEM26	F: ttgcaccatgagacccagt	64
	R: tgctggtattctgtgatgttcc	
TBX1	F: gtgccggtggacgataag	17
	R: cgagtccgggtggtagtg	
HOXC9	F: gcagcaagcacaaagagga	85
	R: cgtctggtacttggtgtaggg	
LEP (leptin)	F: ttgtcaccaggatcaatgaca	25
	R: gtccaaaccggtgactttct	
ADIPOQ (adiponectin)	F: ggtgagaagggtgagaaagga	85
	R: tttcaccgatgtctcccttag	
EBF2	F: ccggctctccttatggaat	11
	R: aaaatgggaggatggaggat	
SLC2A1 (GLUT-1)	F: ggttgtgccatactcatgacc	67
	R: cagataggacatccagggtagc	
SLC2A4 (GLUT-4)	F: ctgtgccatcctgatgactg	67
	R: cgtagctcatggctggaact	
ZNF516 (zinc finger protein 516)	F: gcctcggggttctgaagt	10
	R: gtacctgtgttagaatggtctctcc	

CEBPB	F: cgcttacctcggctacca<br>R: acgaggaggacgtggagag	74
PPARA	F: gcactggaactggatgacag<br>R: tttagaaggccaggacgatct	5
CPT1B	F: gagcagcaccccaatcac<br>R: aactccatagccatcatctgct	10
PNPLA2<br>(ATGL)	F: ctccaccaacatccacgag<br>R: ccctgcttgcacatctctc	89
PER1	F: ctcttccacagctccctca<br>R: ctttggatcggcagtggt	87
CYCS<br>(cytochrome C)	F: tgtgccagcgactaaaaaga<br>R: cctcccttttcaacggtgt	48
ATP5A1<br>(F1-ATPase alpha subunit)	F: tgctattggtcaaaagagatcca<br>R: gtagccgacaccacaatgg	61
PPARG	F: tgacaggaaagacaacagacaaat<br>R: gggtgatgtgtttgaacttgatt	7
FABP4	F: ccaccataaagagaaaacgagag<br>R: gtggaagtgacgcctttcat	31
**Murine samples**		
RN18S	F: ctcaacacgggaaacctcac<br>R: cgctccaccaactaagaacg	77
TBP<br>(TATA box binding protein)	F: gggagaatcatggaccagaa<br>R: gatgggaattccaggagtca	97
UCP1	F: ggcctctacgactcagtcca<br>R: taagccggctgagatcttgt	34
ADRB3<br>(β3-adrenoreceptor)	F: cagccagccctgttgaag<br>R: ccttcatagccatcaaacctg	13
PPARGC1A<br>(PGC-1α)	F: gaaagggccaaacagagaga<br>R: gtaaatcacacggcgctctt	29
CEBPB	F: atcgacttcagcccctacct<br>R: tagtcgtcggcgaagagg	55
PPARA	F: ccttccctgtgaactgacg<br>R: ccacagagcgctaagctgt	5
CPT1B	F: caaggaattccaggacaagact<br>R: cttcccaccagtcactcaca	64
SLC2A1<br>(GLUT-1)	F: ggaccctgcacctcattg<br>R: gccacgatgctcagatagg	20
SLC2A4<br>(GLUT-4)	F: gacggacactccatctgttg<br>R: gccacgatggagacatagc	5
ZFP516	F: ttcgccaccctctaccag<br>R: ggtgtccctgttagtgatgtcc	66
LIPE<br>(hormone sensitive lipase)	F: agcgctggaggagtgtttt<br>R: ccgctctccagttgaacc	3
PNPLA2<br>(ATGL)	F: gagcttcgcgtcaccaac<br>R: cacatctctcggaggacca	89
PER1	F: gcttcgtggacttgacacct<br>R: tgctttagatcggcagtggt	71
CYCS<br>(cytochrome C)	F: aacgttcgtggtgttgacc<br>R: ttatgcttgcctcccttttc	104
ATP5A1<br>(F1-ATPase alpha subunit)	F: gctgaggaatgttcaagcaga<br>R: ccaagttcagggacataccc	31
PPARG	F: gaaagacaacggacaaatcacc<br>R: gggggtgatatgtttgaacttg	7
FABP4	F: ggatggaaagtcgaccacaa<br>R: tggaagtcacgcctttcata	31

Experimental procedures

In vitro studies of BAT activity

Adipose tissue collection in humans

Male and female euthyroid subjects were recruited who were due to undergo elective thyroid or parathyroid surgery in the Royal Infirmary of Edinburgh. During their operation, a small amount of adipose tissue was obtained from the central compartment of the neck, superior to the clavicle and deep to the lateral thyroid lobe either adjacent to the longus colli muscle or to the oesophagus (brown adipose tissue) (Figure S1), and more superficially from the subcutaneous neck tissue (white adipose tissue). This tissue was either immediately frozen, fixed, or the stromal vascular fraction isolated and cultured for the respective experiments. Tissue collections were performed by 2 surgeons.

Adipose tissue collection in mice

Male 129/Ola mice were housed at room temperature (20˚C), 6-8 mice per cage. At 8-9 weeks of age, animals were killed and the interscapular brown adipose tissue, inguinal and epididymal white adipose tissue were immediately collected for isolation of the stromal vascular fraction.

Cell culture studies

Upon collection, adipose tissue was cut into small pieces and digested using 0.2% collagenase type 1 in Krebs-Heinseleit buffer at 37˚C for 45 minutes. Following centrifugation at 600g for 10 minutes at room temperature, the pellet was resuspended and passed through a 100μm filter then subjected to centrifugation at 200g for 5 minutes at room temperature. The pellet was re-suspended in DMEM containing 10% FBS and cultured in 6-well plates. Medium was changed every 2-3 days, and cells were passaged once 80% confluence was achieved. Confluent cells were differentiated in the above

medium with the addition of 1nM tri-iodothyronine, 20nM insulin, 500μM IBMX, 500nM dexamethasone and 125μM indomethacin for 7 days. Cells were then cultured for a further 7 days in DMEM medium containing 10% FBS, 1nM tri-iodothyronine and 20nM insulin prior to experiments. Before experiments testing the effect of varying cortisol concentrations in the medium, cells were deprived of steroids by culture in medium with charcoal stripped serum for 24 hours. Thereafter, depending on the experiments cells were cultured in serum-stripped medium with the addition of varying concentrations of either cortisol, dexamethasone, eplerenone (10μM) or RU38486 (10μM) for incubation periods of between 4 and 48 hours. In the experiments using murine adipocytes, following differentiation half of the wells containing adipocytes from the inguinal depot received 5 days incubation with the β3-agonist CL316,243 (1μM) to induce browning; this was completed prior to commencing incubation with the stripped serum. Experiments were performed after 2-3 passages.

Quantitative real time PCR measurements

Whole adipose tissue (100mg) was homogenised in Qiazol reagent using a rotor-stator homogeniser, and cells were lysed in Qiazol. RNA was extracted using the RNeasy Lipid Kit (Qiagen, Crawley, UK) and cDNA was generated using the Qiagen QuantiTect reverse transcription kit. Real time PCR was performed in triplicate using a Roche Lightcycler 480 (Roche Diagnostics Ltd, Burgess Hill, UK), using gene specific primers (Invitrogen Ltd, Paisley, UK) and fluorescent probes from the Roche Universal Probe Library unless otherwise stated. Primer sequences and probe numbers are described in Table S4. Transcript levels are presented as the ratio of the abundance of the gene of interest: mean of abundance of control genes, with PPIA and 18S used as controls in humans while TBP and 18S were used as control genes in mice.

Histology

Whole adipose tissue was fixed in 10% formalin, paraffin embedded and stained with haematoxylin and eosin prior to viewing on a Zeiss Axioskop. For immunohistochemistry, whole tissue was fixed as above while adipocytes were grown on Superfrost Plus slides and fixed using 10% formalin. Sections were incubated with rabbit anti-human UCP-1 antibody (1:8000 dilution, Sigma U6382) as the primary and goat anti-rabbit (1:200 dilution, Vectastain Elite kit, PK6101) as the secondary antibody according to the avidin-biotin complex method as follows: 1) peroxidase blocking with 3% hydrogen peroxide in methanol for 15 minutes; 2) 20% goat serum for 1 hour to reduce non-specific binding; 3) overnight incubation with primary antibody at 4˚C; 4) incubation with secondary antibody for 30 minutes at room temperature; 5) incubation with the Elite ABC reagent for 30 minutes (Vector Labs, Burlingame, CA); 6) incubation with 3,3'-diaminobenzidine for 2 minutes; 7) counterstaining with haematoxylin prior to mounting in Distyrene Plasticizer Xylene.

Respirometry

Paired pre-adipocytes from human WAT and BAT (n=6 subjects) and murine inguinal and interscapular brown depots were differentiated in XF24 V7 (human) or V28 (murine) Cell Culture Microplates (Seahorse Biosciences, Copenhagen, Denmark). White and brown adipocytes were cultured in serum-stripped medium for 24 hours, and then cultured in serum-stripped medium containing 0, 100 or 1000nM cortisol for 24 hours. Cells were then washed and placed in Seahorse XF Assay medium containing 17.5mM glucose and 1mM pyruvate. Oxygen consumption (OCR) and extracellular acidification rates (ECAR) were quantified in triplicate on a Seahorse XFe24 analyser. Measurements were performed of basal and isoprenaline (2µM) stimulated respiration, following subtraction of non-mitochondrial respiration (measured following the addition of rotenone (0.2µM) and antimycin A (2.5µM)). Basal respiration was calculated by taking the mean of 3 cycles (consisting of 2 minutes mixing, 4 minutes waiting, and 2 minutes of measurements) while stimulated

respiration was measured using the results of the 6th cycle following the addition of isoprenaline when the maximum OCR was achieved.

References

1) http://www.metoffice.gov.uk/public/weather/climate-historic/#?tab=climateHistoric
